# Investigating Alkylated Prodigiosenes and Their Cu(II)‐Dependent Biological Activity: Interactions with DNA, Antimicrobial and Photoinduced Anticancer Activity

**DOI:** 10.1002/cmdc.202100702

**Published:** 2021-12-22

**Authors:** Sebastian Doniz Kettenmann, Matthew White, Julien Colard‐Thomas, Matilda Kraft, Andrea T. Feßler, Karin Danz, Gerhard Wieland, Sylvia Wagner, Stefan Schwarz, Arno Wiehe, Nora Kulak

**Affiliations:** ^1^ Institut für Chemie und Biochemie Freie Universität Berlin Fabeckstr. 34/36 14195 Berlin Germany; ^2^ Department of Chemistry Molecular Sciences Research Hub Imperial College London 80 Wood Lane London W12 0BZ UK; ^3^ Département de chimie École normale supérieure 24 rue Lhomond 75005 Paris France; ^4^ Faculté de médecine Université de Montpellier 2 rue de l'École de médecine 34000 Montpellier France; ^5^ Institut für Chemie Otto-von-Guericke-Universität Magdeburg Universitätsplatz 2 39016 Magdeburg Germany; ^6^ Institut für Mikrobiologie und Tierseuchen Freie Universität Berlin Robert-von-Ostertag-Str. 7 14163 Berlin Germany; ^7^ Fraunhofer-Institut für Biomedizinische Technik IBMT Joseph-von-Fraunhofer-Weg 1 66280 Sulzbach Germany; ^8^ biolitec research GmbH Otto-Schott-Str. 15 07745 Jena Germany; ^9^ Institut für Chemie und Biochemie Freie Universität Berlin Arnimallee 22 14195 Berlin Germany

**Keywords:** prodigiosenes, Cu(II) complexes, DNA cleavage, cytotoxicity, antimicrobial agent

## Abstract

Prodigiosenes are a family of red pigments with versatile biological activity. Their tripyrrolic core structure has been modified many times in order to manipulate the spectrum of activity. We have been looking systematically at prodigiosenes substituted at the **C** ring with alkyl chains of different lengths, in order to assess the relevance of this substituent in a context that has not been investigated before for these derivatives: Cu(II) complexation, DNA binding, self‐activated DNA cleavage, photoinduced cytotoxicity and antimicrobial activity. Our results indicate that the hydrophobic substituent has a clear influence on the different aspects of their biological activity. The cytotoxicity study of the Cu(II) complexes of these prodigiosenes shows that they exhibit a strong cytotoxic effect towards the tested tumor cell lines. The Cu(II) complex of a prodigiosene lacking any alkyl chain excelled in its photoinduced anticancer activity, thus demonstrating the potential of prodigiosenes and their metal complexes for an application in photodynamic therapy (PDT). Two derivatives along with their Cu(II) complexes showed also antimicrobial activity against *Staphylococcus aureus* strains.

## Graphical abstract

In memory of Professor Klaus Hafner.**Prodigiosenes, synthetic analogs** of the natural product prodigiosin, exhibit self‐activated DNA cleavage in the presence of Cu(II). In unprecedented Cu(II)‐dependent cytotoxicity studies, we investigated the role of different alkyl chains at the **C** ring. Cytotoxicity was selective towards cancer cells and could be increased by irradiation. Some prodigiosenes also showed high antibacterial activity.

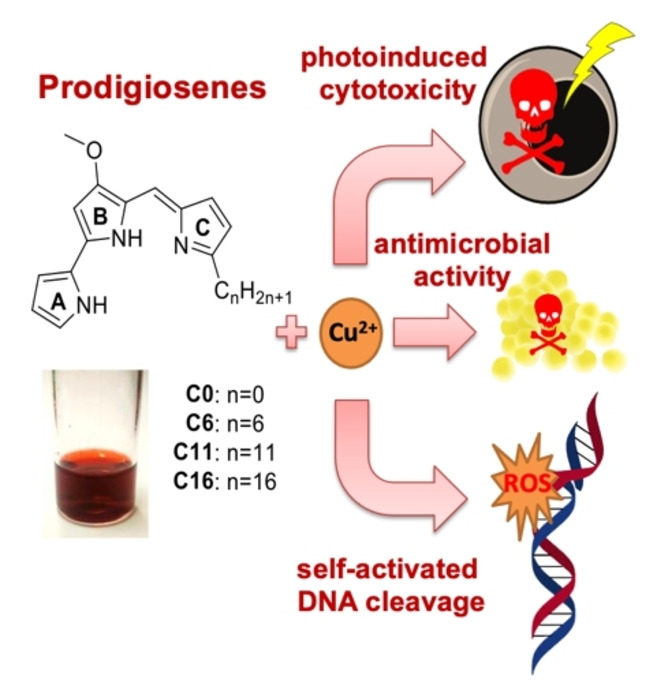

## Introduction

**Figure 1:**
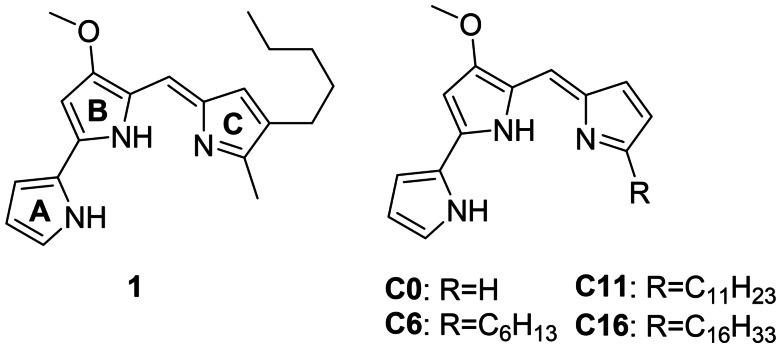
Structure of natural prodigiosin **1** (with **A**, **B**, **C** rings as referred to in the text) and the derivatives studied in this work: α‐unsubstituted (**C0**), hexyl‐ (**C6**), undecyl‐ (**C11**), and hexadecylprodigiosin (**C16**).

Prodigiosenes, often also termed prodiginines, are a family of tripyrrolic compounds based on secondary metabolites isolated mainly from bacteria of the genera *Serratia* and *Streptomyces*.[^1^, ^2^] Several of these natural red pigments, including the first isolated and structurally characterized prodigiosin **1** (Figure 1), have widely been the subject of studies for their promising and relevant biomedical applications, based on their antimalarial, immunosuppressive, anticancer, and antibacterial properties.[^2^, ^3^, ^4^, ^5^, ^6^, ^7^, ^8^]

Undecylprodigiosin **C11** (Figure 1) is a further example of the naturally obtainable prodigiosenes, which was isolated and structurally characterized by different laboratories in the 1960s.[^9^, ^10^, ^11^] In a similar fashion to prodigiosin **1**, multiple biological properties of **C11** have already been documented. D'Alessio and co‐workers evaluated the cytotoxicity and immunosuppressive activity of **C11** during extensive screening for a derivative with an improved selectivity index (i. e. *in vitro* toxicity/immunosuppressive activity ratio).^[12]^ Reynolds *et al*. assessed the antimalarial activity of **C11** in comparison with several other natural and synthetic prodigiosenes.^[13]^ **C11**, among other prodigiosenes, has been recognized to be able to induce apoptosis in several cancer cell lines.[^14^, ^15^, ^16^, ^17^, ^18^]

These prominent attributes have urged the elucidation of the mechanisms behind the biological properties of this compound class, yielding multiple studies that present possible modes of action. It has become evident that the various biological activities of prodigiosenes do not result from one single mechanism, but rather involve multiple cellular targets.[^2^, ^6^] These include the ability of prodigiosenes to transport H^+^/Cl^−^ through membranes[^19^, ^20^, ^21^, ^22^] and to act as dual topoisomerase inhibitors.^[23]^ In addition, the cytotoxic properties have been partially attributed to the Cu(II)‐mediated DNA cleaving properties of prodigiosenes, which ultimately lead to cell apoptosis.[^22^, ^24^, ^25^, ^26^, ^27^, ^28^, ^29^]

In many cases, however, the conclusions about the biological function and mechanisms of action are drawn by analogy with prodigiosin **1** or similar derivatives, without detailed studies that corroborate these assumptions. Several studies concentrate on the screening of multiple derivatives, without further rationalization as to if and how particular substituents could be responsible for certain modes of action. In‐depth investigation of structure‐activity relationships, specially focused on the implications that certain moieties have on different mechanistic pathways, is therefore crucial for future development of prodigiosenes with improved and selective pharmacological activity.

A large part of the cytotoxicity of prodigiosenes is commonly attributed to the structural integrity of the pyrrolylpyrromethene skeleton as represented by derivative **C0** (Figure 1).^[12]^ The importance of the methoxy group (**B** ring) for the *in vitro* cytotoxicity of prodigiosin **1** was established early on by Boger *et al*.^[30]^ and was later corroborated by D'Alessio and co‐workers.[^12^, ^31^] Moreover, the replacement of the pyrrolic **A** or **B** rings with a non‐nitrogen containing heterocyclic moiety also leads to the loss of cytotoxicity, which is generally attributed to a decrease of the Cu(II)‐coordinative ability.[^6^, ^24^, ^27^]

The substitution pattern of the prodigiosene scaffold is possibly the least investigated aspect regarding the implications on the structure‐activity relationship. The aliphatic substitution pattern at the **C** ring is so far widely regarded as having little to no influence on the biological activity,[^12^, ^22^, ^24^, ^32^] even though it has been shown in other cases that such long hydrophobic substituents can have a relevant influence on a compound's cellular uptake and mechanism of action.[^33^, ^34^, ^35^] This is especially the case, when the hydrophobic alkyl chain is connected to a hydrophilic moiety, resulting in an amphiphile. In the case of alkylated prodigiosenes, such amphiphlic character could, for instance, arise from (de)protonation or metal complexation, yielding an ionic amphiphile or a metalloamphiphile, respectively. Previous studies not only showed that alkyl moieties are involved in the binding process of amphiphiles to DNA/RNA^[36]^ and protein subdomains (e. g. HSA or BSA)^[37]^ through hydrophobic interactions; it has also been reported that self‐assembly of metalloamphiphiles can lead to superior biological activity due to multivalency (close proximity between active sites) and cooperative phenomena.[^35^, ^38^, ^39^] In addition to the effects of self‐assembly, hydrophobic moieties can facilitate the interaction with biological membranes, resulting in substrate anchoring^[40]^ or even disruption of the cell membrane that leads to cytotoxic effects.[^41^, ^42^, ^43^]

Considering the relevance such hydrophobic substituents can have in a biological environment and the fact that a closer study regarding this aspect for alkylated prodigiosenes is unprecedented, we decided to perform an extensive and substantial study of the prodigiosenes **C0**, **C6**, **C11**, and **C16** (Figure 1). Despite the biomedical potential mentioned above, **C11** remains one of the less studied members of the natural prodigiosene family.^[2]^ To the best of our knowledge the Cu(II)‐mediated DNA cleavage by **C6**, **C11**, and **C16** has not been described before.^[13]^ The α‐unsubstituted prodigiosin **C0** has only briefly been mentioned in comparative studies regarding the Cu(II)‐mediated DNA cleavage and H^+^/Cl^−^ transport capabilities of prodigiosene derivatives.[^22^, ^24^]

This work includes revisited synthetic routes for prodigiosenes **C0**, **C6**, **C11** and **C16**, their structural characterization, and the investigation of their Cu(II)‐complexation capabilities. Furthermore we performed multiple experiments for the elucidation of the mechanisms behind their DNA binding and cleaving activity, and finally assessed the implication of these results on the photoinduced cytotoxic and antimicrobial activity of these compounds in presence and absence of Cu(II).

## Results and Discussion

### Synthesis of prodigiosenes

The early rise in interest for **1**, **C11**, and other prodigiosenes resulted in the increasing investigation of synthetic methods to easily access natural and synthetic derivatives.^[5]^ One of the prominently established strategies was published by D'Alessio and Rossi, who described the preparation of undecylprodigiosin **C11** through a straightforward five‐step synthesis.^[44]^ We followed this approach with some modifications (Scheme S‐1), specifically regarding the Suzuki‐Miyaura cross coupling step, to also synthesize the structurally similar prodigiosene derivatives **C0**, **C6**, and **C16**. The synthesis of the latter compounds has only been reported through other approaches.^[13]^ We are thus presenting here the isolation of new dipyrrin and dipyrrinone precursors which so far have not been described in the literature (S‐2). To ensure a suitable grade of purity of the final compounds for the biological studies, the alkylated prodigiosenes **C6**, **C11**, and **C16** were isolated by column chromatography over basic aluminum oxide, precipitated as the hydrochloric salts from a concentrated solution in ethanol, and subsequently purified through HPLC. Protonation of **C0**, on the other hand, resulted in the gradual decomposition of the compound over time. For this reason, compound **C0** was isolated and stored as a free base. All prodigiosenes were fully characterized through ^1^H NMR, ^13^C NMR, ESI‐MS, and analytical HPLC measurements (S‐3).

### Cu(II) complexation

Even though the Cu(II)‐mediated DNA cleavage by prodigiosenes has widely been reported in the literature, the synthesis and isolation of the Cu(II) complexes remains elusive. To the best of our knowledge, the only intact Cu(II)‐prodigiosene 1 : 1 complexes have been obtained by Tomat *et al*. by introducing a coordinating ester functionality at the prodigiosene scaffold, thus generating a tetradentate ligand.^[45]^

In contrast, other prodigiosenes coordinate to Cu(II) as tridentate ligands. Manderville *et al*. first characterized a distorted square‐planar Cu(II) complex of the natural prodigiosin **1** by generating the complex under basic conditions (pH≈10). The molecular structure of the complex in the solid state revealed the oxidation of the ligand after complexation, a process that can be corroborated by mass spectrometry.^[29]^ These results are directly correlated with the intrinsic redox activity of the Cu(II)‐prodigiosene system.

The observed oxidized products possibly stem from the formation of a ligand‐based π‐radical cation, a species that might be involved in the self‐activated, Cu(II)‐mediated DNA cleavage activity of prodigiosenes.^[24]^ For the application in biological systems it is, however, important to characterize the complexation process under physiological conditions (pH 7.4), in order to be able to attribute the biological activity to the Cu(II)‐prodigiosene complexes.^[27]^

**Figure 2:**
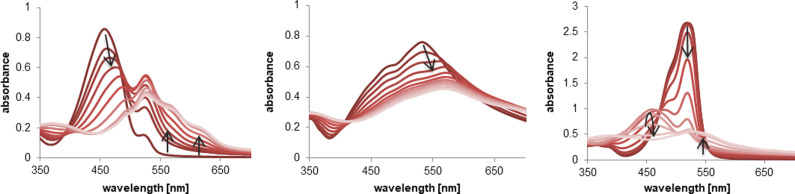
UV/VIS titration experiments of hexylprodigiosin **C6** with 0.1 eq. aliquots of Cu(OAc)_2_ performed in MeOH at pH 10 (left), in MOPS buffer at pH 7.4 (middle), and in acetonitrile without buffer (right).

As expected,^[24]^ the isolation of the Cu(II) complexes after addition of copper(II) acetate to prodigiosenes **C0**, **C6**, **C11**, and **C16** was not successful. Nevertheless, we were able to confirm the Cu(II) complexation in solution through UV/VIS spectroscopy (Figure 2 for **C6**, S‐3.5 for **C0**, **C11**, and **C16**) and mass spectrometry (S‐3.6).

When examining the formation of the Cu(II) complexes for **C6** through UV/VIS titration experiments in MeOH around pH 10^[29]^ and in water at pH 7.4, the characteristic bathochromic shift of the absorption band was observed when transitioning from the free ligand to the 1 : 1 Cu(II) complex **CuC6** (Figure 2, left and middle). However, a gradual change in the absorption maximum and the lack of an isosbestic point is indicative of a transition state. This process is more prominent when performing the complexation in pure acetonitrile, wherein an absorption band at a lower wavelength (for **CuC6** at 458 nm) emerges and disappears during the titration experiment (Figure 2, right). A similar behavior has been reported before by Thompson *et al*. and is attributed to the formation of the 1 : 2 Cu(II):prodigiosene complex that transitions to the 1 : 1 complex after surpassing 0.6 eq. of the Cu(II) salt.^[32]^

In fact, when analyzing the complex solution in acetonitrile through ESI mass spectrometry, both, the ML and ML_2_ complexes, could clearly be observed (Figure S‐3.6.3). The *m/z*‐signals obtained under these conditions belong to the reduced Cu(I) species, which is a common result when performing ESI‐MS measurements of Cu(II) complexes in acetonitrile.[^46^, ^47^] ESI‐MS of **CuC6** in methanol (Figure S‐3.6.4), on the other hand, shows a mixture of two oxidized species: one with an attached methoxy group and one as azafulvenic derivative (Scheme S‐3.6). The formation of these oxidized species is, of course, favored under the ionizing conditions of the ESI‐MS measurement. These findings are in accordance to the aforementioned results described by Manderville and co‐workers for prodigiosin **1**.^[29]^

Interestingly enough, mass spectrometric analysis of the Cu(II) complexation by the α‐unsubstituted prodigiosene **C0** in methanol did not show the generation of the oxidized derivatives as found for the other derivatives, and only a complex product mixture with higher mass to charge ratios was observed (Figure S‐3.6.2). In this case, multiple signals can be associated with ML_2_ or M_2_L_2_ complexes. The lack of a ML complex species indicates that the radical cation generated under the ionizing conditions is more reactive in the case of the α‐unsubstituted prodigiosene than of the alkylated derivatives, leading to degradation of the ligand in a similar way to the polymerization of pyrroles by Cu(II) salts as reported in the literature.[^48^, ^49^]

Given that in general the formation of a 1 : 1 complex is favored at equimolar concentrations of the Cu(II) salt in aqueous buffered solution, as shown by the results mentioned above, it is safe to assume that this is the relevant active species during the subsequent biological studies. Following the example of previous studies presented in the literature,[^22^, ^24^, ^26^, ^27^, ^28^] the complexes in all further experiments were generated *in situ* through addition of equimolar quantities of copper(II) acetate.

### DNA binding studies

The interaction of prodigiosenes **C0**, **C6**, **C11**, and **C16** with calf thymus DNA (CT‐DNA) in presence of Cu(II) was studied through circular dichroism (CD) and fluorescence spectroscopy as well as DNA‐melting analysis. Since the interactions of the free ligands with DNA are minor, only the respective results of **C0** and **C11**, as a representative of the alkylated derivatives, are given as comparison.

**Figure 3:**
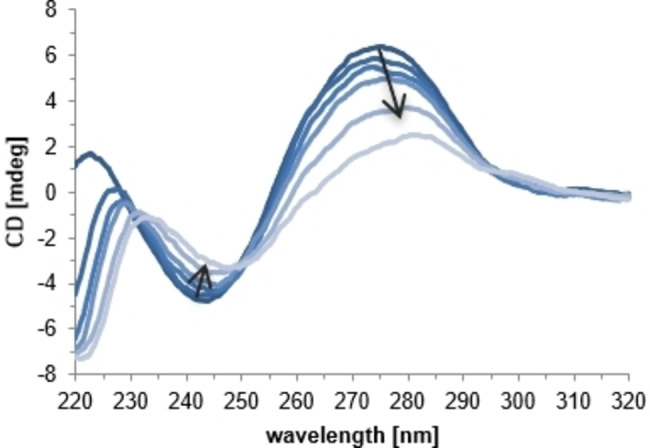
CD spectrum of CT‐DNA (100 μM) in MOPS buffer (10 mM, pH 7.4) with increasing concentrations of *in situ* formed **CuC6** (0, 5, 10, 15, 30, 50 μM).

When examining CT‐DNA *via* CD spectroscopy it exhibits a negative band at 245 nm, attributed to the helicity of the B form, and a positive band at 275 nm, commonly ascribed to the stacking of the DNA nucleobases.^[50]^ A change in the intensity of the negative or the positive band upon addition of the investigated compound is an indication of its interaction to DNA *via* groove binding or intercalation, respectively.[^51^, ^52^, ^53^] The only ligand showing a notable influence on the DNA structure was the non‐alkylated derivative **C0**, interacting predominantly through intercalation (Figure S‐4.1). Coherently, the degree of DNA interaction of the studied prodigiosenes in presence of Cu(II) decreased with increasing chain length of the alkyl substituent in the order **CuC0**>**CuC6**>**CuC11**≈**CuC16** (Figure 3 for **CuC6**, S‐4 for the other complexes). Although all four complexes show a preferable intercalative DNA binding mode, a decrease of their intercalative capabilities in the aforementioned order is associated with an increase in their interaction through groove binding. The sterically demanding alkyl substituent clearly hinders an intercalation of the aromatic prodigiosene core into the DNA and lowers the binding affinity of both, the free ligands and the copper complexes.

**Figure 4:**
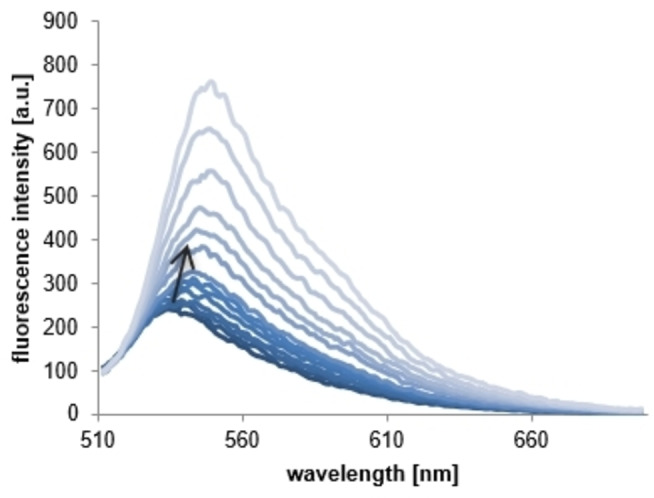
Fluorescence emission spectral titration (*λ*_ex_=500 nm) of prodigiosene **C0** (40 μM) in MOPS buffer (10 mM, pH 7.4) with increasing concentrations of CT‐DNA (0, 2, 4, 6, 8, 10, 12, 14, 20, 25, 30, 40, 50, 60 μM).

When exciting **C0** at 500 nm, a fluorescence maximum at 537 nm can be observed (Figure 4). A similar behavior has previously been reported for the natural prodigiosin **1** by Han *et al*.^[54]^ This fluorescence behavior by prodigiosene **C0** provides the possibility of observing the interaction with DNA through variation of the fluorescence maximum during the titration of CT‐DNA. Similar to the behavior observed for the well‐known intercalator ethidium bromide (EB),^[55]^ the fluorescence maximum of **C0** increased distinctly with the rising CT‐DNA concentration, a property that we attribute to the transition into a hydrophobic environment, when **C0** intercalates into the DNA (Figure 4).

Although both prodigiosenes **C0** and **C11** emit weak fluorescence in buffered solution (pH 7.4) at room temperature (Figure S‐7), this behavior is remarkably stronger for the α‐unsubstituted derivative **C0** than for undecylprodigiosin **C11**, an alkylated example. The ligand fluorescence of the prodigiosenes was completely quenched by addition of copper(II) acetate, as expected from the complexation of the paramagnetic Cu(II) center (Figure S‐7).^[56]^

EB displacement experiments were performed in order to additionally determine the binding affinity of the Cu(II) complexes towards CT‐DNA (S‐6). Although quenching of the EB fluorescence by the paramagnetic Cu(II) ion is feasible,^[56]^ this effect is negligible (measurement not shown here) in comparison to the loss in fluorescence intensity through actual EB displacement from the DNA binding sites. It is to be noted specifically in the case of the ligand **C0** that, even though the fluorescence maxima between the **C0**‐DNA complex (550 nm) and the EB‐DNA complex (600 nm) differ from each other, an overlap at higher ligand concentrations is feasible. The observed hypsochromic shift of the fluorescence maximum at higher **C0** concentrations is a sign of the generation of a mixed EB‐DNA‐**C0** complex (Figure S‐6.1).^[54]^ For this reason only concentrations that did not deviate from the linearity of the Stern‐Volmer‐Plot (up to 30 μM) were used for the calculation of the apparent binding constant *K*_app_ of **C0**.^[57]^

The binding constants were calculated from the Stern‐Volmer relationship (S‐6, Table 1). The calculated constants further confirm the results of the CD and fluorescence experiments: the binding affinity of the *in situ* formed Cu(II) complexes is superior to the one of the ligands alone, and the DNA binding capability decreases with increasing substituent size in the order **CuC0**>**CuC6**≫**CuC11**≈**CuC16**>**C0**>**C11**. The range of the apparent binding constants *K*_app_ moreover indicates that **CuC0** and **CuC6** predominantly bind to DNA by an intercalative mode, since the values are comparable to the binding constant of the competing intercalator EB (10^6^–10^7^ M^−1^).[^54^, ^57^, ^58^] **CuC11** and **CuC16**, on the other hand, show considerably lower binding constants, suggesting that groove binding and/or electrostatic interactions have a higher contribution to the DNA binding process of these complexes.

**Table 1:** Stern‐Volmer constants *K*_SV_ and binding constants *K*_app_ for the binding process to CT‐DNA by the prodigiosenes **C0** and **C11** and the copper complexes **CuC0**, **CuC6**, **CuC11**, and **CuC16**.

compound	*K*_SV_ [M^−1^]	*K*_app_ [M^−1^]
**C0**	1.19×10^4^	1.55×10^5^
**C11**	1.01×10^4^	1.31×10^5^
**CuC0**	1.16×10^5^	1.50×10^6^
**CuC6**	9.41×10^4^	1.22×10^6^
**CuC11**	4.41×10^4^	5.73×10^5^
**CuC16**	4.42×10^4^	5.75×10^5^

The influence of the *in situ* formed complexes on the thermal stability of CT‐DNA was examined *via* CT‐DNA melting experiments (Table 2). An increase in the melting temperature of DNA is attributed to a higher stability caused by interaction of the substrate with the double helix.^[59]^ Only a minimal stabilization was observed in presence of Cu(OAc)_2_ or the ligands alone, while the *in situ* formed complexes showed a more prominent influence on the thermal stability of DNA (Figure S‐5). Furthermore an increase in double‐stranded DNA (dsDNA) stability was observed, when increasing the alkyl chain length in the order **CuC0**<**CuC6**<**CuC11**<**CuC16**. At first glance these results stand in contrast to the previous experiments, since generally an increasing intercalation capability should lead to a higher thermal stabilization of the dsDNA towards denaturation.^[59]^ However, in this case the thermal stabilization has to be attributed to the aliphatic substituent. The hydrophobic moiety stabilizes the dsDNA in a significant way, compensating the partial loss of intercalating ability that has been exhibited in the previous experiments.

**Table 2:** Thermal denaturation temperature *T*_m_ of CT‐DNA and the temperature difference Δ*T*_m_ in absence and presence of 2.5 μM Cu(OAc)_2_ and compounds **C0**, **C11**, **CuC0**, **CuC6**, **CuC11**, and **CuC16**.

compound	*T*_m_ [°C]	Δ*T*_m_ [°C]
CT‐DNA (Ref)	70.8	–
Cu(OAc)_2_	71.2	0.4
**C0**	71.7	0.9
**C11**	71.5	0.7
**CuC0**	75.2	4.3
**CuC6**	76.8	6.0
**CuC11**	78.1	7.3
**CuC16**	78.8	8.0

Although the exact reason behind this observation is not clear, the ability of amphiphilic compounds to stabilize the dsDNA during denaturation or renaturation experiments has been reported in the literature before.[^60^, ^61^, ^62^, ^63^]

The results of the DNA melting experiments do not give additional insight into the DNA binding mode, but the increase in melting temperature for all Cu complexes confirms the strong interaction between the metalloprodigiosenes and DNA. This increase in binding affinity for the Cu(II) complexes compared to the free ligands can be explained, on the one hand, by a stronger electrostatic attraction of the cationic complex towards the anionic phosphate backbone of DNA. On the other hand, the coordination to a metal center forces the prodigiosene core structure into a quasi‐planar conformation,^[29]^ facilitating the intercalation into the DNA.

### DNA cleavage

The Cu(II)‐mediated DNA cleavage of prodigiosenes **C0**, **C6**, **C11**, and **C16** was investigated by the means of agarose gel electrophoresis, using supercoiled plasmid DNA pBR322. Physiological conditions during 1 h incubation at 37 °C were maintained at pH 7.4 through addition of 10 mM MOPS buffer and 100 mM NaCl to emulate the pH and ionic strength of a cellular environment.^[22]^ The complexes were freshly synthesized *in situ* for each experiment through equimolar addition of Cu(OAc)_2_ to the incubation solution.

**Figure 5:**
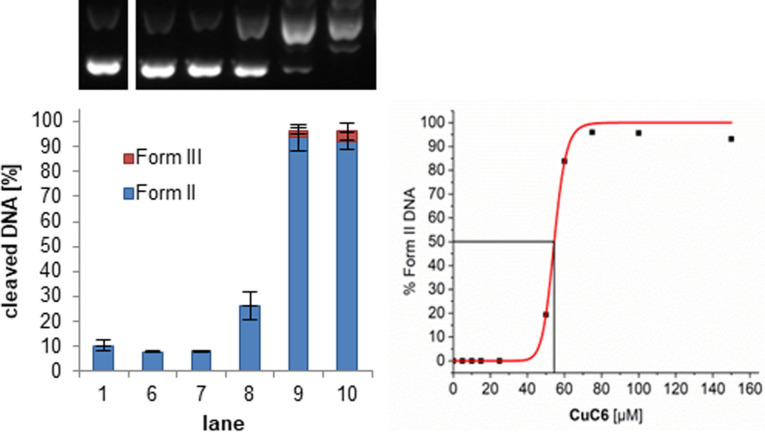
(Left) Agarose gel and visualization of the cleavage of plasmid DNA pBR322 (0.2 μg) with increasing concentrations of *in situ* formed complex **CuC6** in MOPS buffer (10 mM, pH 7.4, 100 mM NaCl) after incubation for 1 h at 37 °C. Lane 1: DNA reference, lane 2–5: omitted for clarity (see S‐8), lane 6: **CuC6** (15 μM), lane 7: **CuC6** (25 μM), lane 8: **CuC6** (50 μM), lane 9: **CuC6** (75 μM), lane 10: **CuC6** (100 μM). (right) Graphical visualization of the calculated EC_50_ value for **CuC6**.

All four complexes cleave DNA without the need of an external reducing agent (S‐8). The ligands and Cu(OAc)_2_ alone do not show any cleaving activity under the studied conditions (S‐8), which confirms that the observed cleavage stems from the complexes formed *in situ*. Through study of the concentration‐dependent DNA cleavage (Figure 5 for **CuC6**), EC_50_ values were determined (Table 3). These values represent the complex concentration needed to cleave 50 % of the initial form I DNA to its form II under the previously mentioned incubation conditions. Although all derivatives have similar EC_50_ values, a trend can be identified for the alkylated derivatives, in which the cleavage efficiency clearly decreases in the order **CuC6**>**CuC11**>**CuC16**.

**Table 3:** EC_50_ values for the cleavage of plasmid DNA pBR322 by the *in situ* formed complexes **CuC0**, **CuC6**, **CuC11**, and **CuC16** after 1 h incubation at 37 °C.

compound	EC_50_ [μM]
**CuC0**	67.3±0.2
**CuC6**	54.4±0.5
**CuC11**	59.9±0.1
**CuC16**	65.0±0.1

This is in accordance with our studies regarding the DNA binding affinity, suggesting that the decrease in cleavage activity in the aforementioned order is not correlated with a lower capability of creating DNA damaging ROS, but rather connected to the decline in the interaction with DNA due to the steric hindering of the alkyl substituent. The complex **CuC0** deviates from this tendency, exhibiting the highest EC_50_ value of all four compounds. However, **CuC0** is the only case in which an aggregation of DNA in the pockets of the agarose gel (Figure S‐8.1) has an impact on the cleavage activity. **CuC6** shows small indications of such a DNA aggregation at higher concentrations, however without notably affecting the DNA cleavage process.

In the case of **CuC0**, this effect is responsible for the inferior cleavage activity and could be correlated to the dimerization and polymerization process that the ligand is able to undergo, as previously acknowledged during the complex characterization (*vide supra*). A dimeric or polymeric species, especially at the higher concentrations (≥50 μM) in which DNA aggregation is observed, could be able to intercalate in multiple DNA strands or strand sections, leading to the formation of the observed DNA aggregates. On the other hand, it is also feasible that a ligand‐localized π‐radical could directly react with DNA segments, leading to interstrand crosslinking with the same aggregation effect.

Addition of an external reducing agent, i. e. ascorbic acid, which is present in cellular environment,^[64]^ greatly increased the cleavage efficiency of all complexes (Figure S‐8.5). This was expected, due to the fact that this additive promotes the redox cycle that generates Cu(I) and drives the generation of ROS. In order to further investigate the cleavage mechanism of the Cu‐prodigiosene species, ROS quenching assays were performed (Figure S‐8.6 for **CuC0**, Figure S‐8.7 for **CuC11**). These inhibition experiments have so far only been performed for prodigiosin **1**,^[27]^ and structurally comparable dipyrrolic^[48]^ or tetrapyrrolic^[28]^ compounds. In the case of prodigiosin **1** singlet oxygen and hydrogen peroxide were determined as the main ROS involved in the Cu(II) mediated DNA cleavage.

The scavengers used in the present work were DMSO^1^ (for hydroxyl radicals, ⋅OH),^[65]^ NaN_3_ (for singlet oxygen, ^1^O_2_),^[66]^ pyruvate (for hydrogen peroxide, H_2_O_2_),^[67]^ and superoxide dismutase (SOD for superoxide radical anions, O_2_^.−^).[^68^, ^69^] The fact that SOD's quenching towards superoxide generates hydrogen peroxide makes it difficult to fully ascertain the involvement of the superoxide species. One additional lane containing SOD and the peroxide scavenger pyruvate was used as a control to verify the presence of the superoxide anion radical.[^69^, ^70^] The results ensured that DMSO does not have a relevant influence on the cleavage activity, thus proving that hydroxyl radicals are not involved as ROS in the DNA cleavage process. The quenching assays (Figure S‐8.6 for **CuC0**, Figure S‐8.7 for **CuC11**) reveal that the predominant ROS in the case of the **CuC0** complex is singlet oxygen, quenched by NaN_3_, with a small amount of hydrogen peroxide, quenched by pyruvate. It is to be noted that the observed DNA aggregation by **CuC0** is not inhibited by the presence of any of the scavengers.

Complex **CuC11**, on the other hand, shows a much larger amount of peroxide formation involved in the cleaving process, as well as a considerable generation of superoxide radicals, demonstrated by the quenching through SOD. This is an unexpected result, as it shows that the alkyl substituent, which should not have an influence on the redox properties of the Cu(II)‐prodigiosene system, indeed has an impact on the ROS formation during the DNA cleavage process. There are two possible explanations for this observation: either the formation of peroxide and superoxide is only enabled when the complex is not tightly interacting with DNA through intercalation, as it is the case for **CuC11**, or the radical degradation of **CuC0** that ultimately leads to DNA aggregation hinders the redox process involved in the generation of these two ROS.

Although these scavenger experiments do not fully rule out the direct involvement of a ligand‐based π‐radical during the DNA damage, they clearly display the generation of ROS, predominantly singlet oxygen, as a central mechanism responsible for the DNA strand scission by Cu(II)‐prodigiosenes.

### Cytotoxicity

The cytotoxic properties of prodigiosin **1** and its derivatives have widely been reported in the literature.[^2^, ^3^, ^7^] Although *in vitro* Cu(II) complexation followed by the cleavage of cellular DNA is regarded as one of the main mechanisms, through which prodigiosenes exert their cytotoxicity,[^22^, ^24^, ^25^, ^26^, ^27^, ^28^, ^29^] we found a lack of studies dealing with the actual cytotoxic activity of the Cu(II) complexes of prodigiosenes. Furthermore, only few studies are known to report the photosensitizing ability of prodigiosenes and their potential for application in photodynamic therapy (PDT). This field of research has been more widely dominated by tetrapyrrolic (i. e. porphyrins, chlorins and phthalocyanines)[^71^, ^72^, ^73^] and dipyrrolic compounds (i. e. BODIPYs^[74]^). Roth first reported in 1967 that the visible light irradiation of bacterial *Sarcina lutea* cells in presence of prodigiosin **1** caused apoptosis.^[75]^ Almost 40 years later, Manderville *et al*. studied the anticancer activity of **1** and other synthetic prodigiosenes against HL‐60 cancer cells in the dark and under light irradiation.[^76^, ^77^] They postulated that photooxidation of prodigiosin to a π‐radical cation, similar to the mechanism behind the Cu(II)‐mediated ROS generation, would reductively transform oxygen into superoxide, subsequently leading to cell damage. Thompson *et al*. further studied the light‐induced cytotoxic activity of prodigiosenes with and without estrogen receptor targeting moieties.^[78]^ They used 9,10‐dimethylantracene (DMA) as a singlet oxygen scavenger, therefore confirming a type II photooxidation mechanism that involves the formation of singlet oxygen. Photoinduced toxicity of the Cu(II) complexes has not been studied before, probably in large part because of the expected high dark cytotoxicity of these compounds.

**Figure 6:**
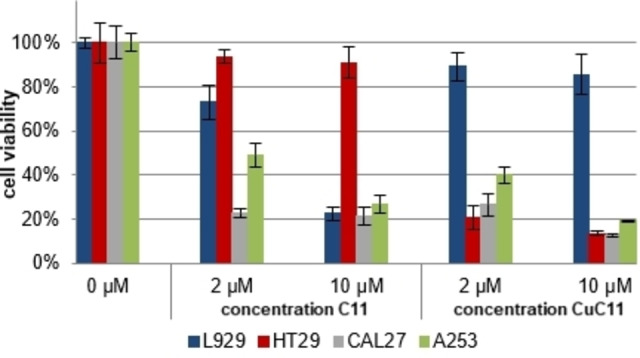
Cell testing of **C11** and *in situ* formed **CuC11** against L929, HT29, CAL27, and A253 cell lines (no irradiation).

**Figure 7:**
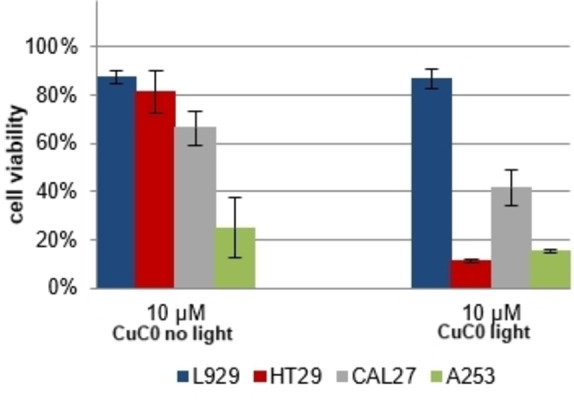
Cell testing of *in situ* formed **CuC0** against L929, HT29, CAL27, and A253 cell lines without (left) and with (right) white light irradiation.

We studied the cytotoxicity of compounds **C0**, **C6**, **C11**, **C16**, and their *in situ* formed Cu(II) complexes in presence and absence of light against the non‐tumor mouse fibroblast cell line L929 and three tumor cell lines: human epidermoid carcinoma cell line A253, squamous carcinoma cell line CAL27, and colorectal adenocarcinoma cell line HT29 (Figures 6, 7, see also S‐9). Cells were incubated for 24 h with 0, 2, and 10 μM concentrations of the prodigiosene derivatives in absence and presence of equimolar amounts of copper acetate. Thereupon the medium was changed prior to irradiation with a white light source to ensure that only compound that had been taken up by the cell contributes to the effect. After a further 24 h incubation the cell viability of the cells was determined *via* the XTT test^[79]^ (see S‐1).

A control experiment showed that copper acetate alone exhibits no significant toxicity in the presence or absence of light (Figure S‐9.1). All alkylated ligands presented a notable cytotoxicity in the dark at 2 and 10 μM concentrations, while the α‐unsubstituted **C0** had a comparably low cytotoxic effect at the same concentrations (S‐9). HT29 tumor cells mostly maintained their viability in the presence of the prodigiosenes, with **C16** showing the highest toxicity of all four ligands at 10 μM concentration (50 % viability). This higher resistance of HT29 tumor cells has also been found with respect to treatment with chemotherapeutic agents.[^80^, ^81^]

The dark toxicity of all four prodigiosenes against the tested tumor cell lines notably increased in the presence of Cu(II) (Figure 6 for **C11** and **CuC11**, S‐9 for full overview), as was to be expected from the generation of cell damaging ROS by the Cu(II) complexes (*vide supra*). Specifically, the HT29 cell line, which showed a comparably low susceptibility against the ligands alone, exhibited a clearly lower viability in presence of the Cu(II) complexes.

Remarkably, the situation is different for the non‐tumor cell line L929, which showed higher viability values when incubated with the *in situ* prepared Cu(II) complexes (73–87 % viability at 10 μM concentration) than in the presence of the ligands alone (22–77 % viability at 10 μM concentration). **CuC11** (Figure 6) is the most prominent example, showing overall high toxicity towards the tumor cells already at 2 μM concentration (21–40 % viability), while only marginally affecting the non‐tumor fibroblast cells (89 % viability).

We attribute this selectivity on the one hand to an increased cell internalization of the Cu(II) complexes by the tumor cells, and a favored ROS generation due to elevated intracellular levels of the reductant glutathione as reported before e. g. for lung cancer cells *vs*. lung fibroblasts.^[82]^ On the other hand the increased cytotoxicity of the free ligands towards the healthy fibroblast cell line lies on a possible toxic effect based on the chelation of essential metal cations[^83^, ^84^] or on the transport of H^+^/Cl^−^ through the cell membrane,[^19^, ^20^, ^21^, ^22^] which regardless of the cell type disrupts the cell's homeostasis. These two mechanisms are significantly hindered for the complexes because of the already present coordination to Cu(II).

Irradiation with visible light led to moderately increased cytotoxicity, although more defined in the case of the complexes than for the ligands (*cf*. S‐9 for full overview). In similar fashion to the prodigiosenes previously studied in the literature,[^76^, ^77^, ^78^] the irradiation of **C6**, **C11**, and **C16** with light probably induces the formation of singlet oxygen *via* the generation of a π‐radical cation of the prodigiosene, which in turn also favors the ROS formation in the case of the redox‐active Cu(II) complexes. Although no clear trend between different lengths of the alkyl substituent is observed in the dark or after irradiation, the inferior cytotoxicity of **C0** and its complex **CuC0** compared to the alkylated analogues shows an undeniable effect of the hydrophobic substituent on the cytotoxic properties of this family of compounds.

It is feasible that the lipophilic alkyl substituent plays an important role during the compounds’ cell internalization process.[^85^, ^86^] Moreover, this hydrophobic moiety can also lead to additional modes of action, as is the disruption of cell membrane structure,[^41^, ^42^, ^43^] which increase the overall cytotoxicity. Nevertheless, regarding a possible application in PDT, any increase in cytotoxicity that is not based on the same mechanism behind the phototoxicity is counterproductive. It is for this reason that complex **CuC0** (Figure 7), which has a more limited cytotoxicity in the dark, is more suitable for a potential application in PDT, since this complex exhibits a clear photoinduced enhancement of its cytotoxic effect on all tested tumor cell lines, while still showing comparably low toxicity towards the non‐tumor L929 cell line.

**Figure 8:**
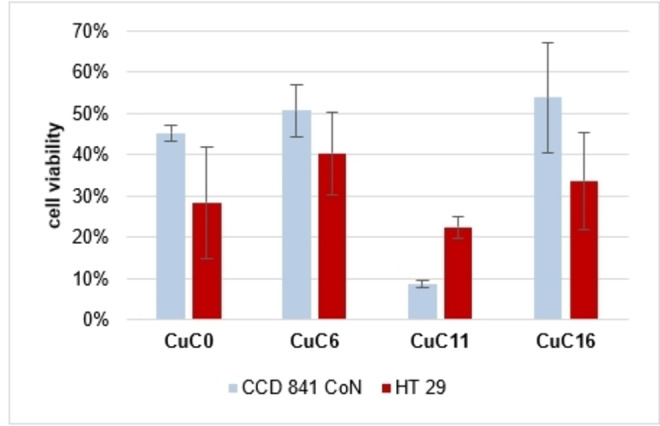
Cell testing of *in situ* formed copper complexes of 2 μM **C0, C6, C11** and **C16** against HT29 and CCD 841 CoN cell lines with white light irradiation[NK1] .

Since L929 cells are from murine mesenchymal origin, cytotoxicity tests were additionally carried out also with CCD 841 CoN cells, a non‐tumor cell line from human epithelial origin (colon), i. e. of the same origin as the tumor cell line HT29. CCD 841 CoN cells were, in general, more susceptible than the HT29 cells to the ligands and complexes in the absence and presence of light (S‐9). Interestingly, however, with the exception of **CuC11**, CCD 841 CoN cells showed higher viability at a concentration of 2 μM of the complexes than HT29 cells under irradiation (Figure 8). Among the complexes, again **CuC0** showed the most distinct discrimination between healthy and cancer cells.

### ROS production in cells

CellROX® Orange was used to detect ROS in live cells (HT29 and CCD) by flow cytometry. For both cell lines, ROS activity was highest without light for **C11** followed by **C6** and their respective complexes, whereas all other compounds showed neglectable ROS generation with and without light (S‐10).

These results stand in contrast to the observations for:

1) the ROS‐based DNA cleavage activity, where Cu(II) indeed plays a decisive role (Note: For the trends among the different prodigiosenes, DNA binding has to be considered, *vide supra*);

2) the cytotoxic activity, where:

‐ Cu(II) also had a positive impact;

‐ depending on the conditions (concentration/light) healthy cells, L929 and CCD 841 CoN, were less affected than cancer cells like HT29;

‐ irradiation with light increased the activity.

It has to be considered that ROS testing by flow cytometry was done with live cells (only 1 h incubation), whereas cytotoxicity studies required much longer incubation times with the compounds (24+24 h). Nevertheless, it is not possible to conclude that ROS‐based DNA cleavage (as observed outside of cells) or ROS generation alone is responsible for cell death. Other factors might also be triggering cell death. Accordingly, it has been shown for natural prodigiosin before, that intracellular ROS formation is not solely responsible for inducing apoptosis, but also cell cycle arrest.^[87]^

### Antimicrobial susceptibility testing

The antimicrobial activity of prodigiosenes has rarely been studied before.[^88^, ^89^] Here, **C0**, **C6** and **C16** as well as their copper complexes were investigated by determination of minimal inhibitory concentrations (MICs).[^90^, ^91^] The MIC indicates the lowest concentration of a substance that prevents visible growth. As such the MIC is an indicator of the antimicrobial efficacy of a substance; the lower the MIC, the more efficient is a substance in inhibiting the growth of or killing a bacterium. Six reference strains belonging to four bacterial species (Gram‐positive: *Staphylococcus aureus* and *Enterococcus hirae*; Gram‐negative: *Escherichia coli* and *Pseudomonas aeruginosa*) were used.

The two *S. aureus* reference strains showed the lowest MICs for **C0** and **CuC0** as well as **C6** and **CuC6** of all tested reference strains (Table 4). MICs ranging between 0.5 and 2 mg/L suggest an activity of these substances against staphylococci. The *E. hirae* and *P. aeruginosa* reference strains still grew in high concentrations of all tested substances, whereas intermediate to high MICs were observed for the two *E. coli* reference strains. The finding that prodigiosenes are more active against *S. aureus* than *E. coli* is in accordance with the published literature.[^92^, ^93^]

**Table 4:** Minimal inhibitory concentrations of the reference strains.

Bacterial strain ID	Minimal inhibitory concentrations [mg/L]					
	**C0**	**CuC0**	**C6**	**CuC6**	**C16**	**CuC16**
*S. aureus* ATCC**®** 29213	0.5	1	1	2	64	64
*S. aureus* ATCC**®** 6538	0.5	0.5	1	1	≥256	≥256
*E. hirae* ATCC**®** 10541	≥8	≥8	≥256	≥256	≥256	128
*E.coli* ATCC**®** 25922	≥256	≥256	64	64	≥256	≥256
*E. coli* ATCC**®** 10536	≥256	≥256	16	32	128	64
*P. aeruginosa* ATCC**®** 15442	≥256	≥256	≥256	≥256	≥256	≥256

**Figure 9:**
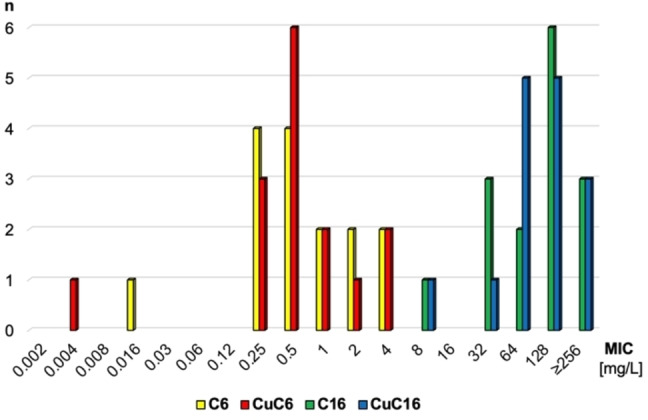
MIC distribution of the staphylococcal field isolates for **C6**, **CuC6**, **C16** and **CuC16** (n=number of isolates).

Since the lowest MICs were observed for the two *S. aureus* reference strains, the MIC values of 15 staphylococcal field isolates (Table S‐11) were determined (Figure 9). The MICs of **C6** and **CuC6** ranged from 0.004 to 4 mg/L. In comparison, the MICs for **C16** and **CuC16** were much higher with MICs of 8 to ≥256 mg/L.

Staphylococcal field isolates exhibited lower MICs for **C6** and **CuC6** compared with **C16** and **CuC16**, which is in accordance with the results seen for the two *S. aureus* reference strains. Interestingly, one *Staphylococcus pseudintermedius* isolate from a dog had remarkably lower MICs (0.016 mg/Lfor **C6** and 0.004 mg/L for **CuC6**) compared with the remaining isolates tested. The results suggest that the presence of Cu(II) does not play a decisive role for the antimicrobial activity. The prodigiosene ligands alone were similarly effective (or ineffective depending on the strain). ROS formation by the prodigiosenes themselves might be responsible for the antibacterial activity.^[89]^

For the *S. aureus* and *S. pseudintermedius* strains, the inhibitory effects decreased with increasing length of the alkyl chain at the prodigiosene, whereas for the other strains no clear trend could be observed. Such strain‐dependent behaviour regarding alkyl chain lengths has been observed before in the literature for other compounds.^[94]^

## Conclusion

Within this study we investigated the Cu(II) complexation and biological activity of prodigiosin derivatives bearing hexadecyl, undecyl, hexyl or no substituent at the α‐position of the **C** ring. One of the main motivations was to assess the impact of a hydrophobic moiety on the biological activity of this compound family. It is important to point out that none of the experiments indicate an aggregation or the formation of micelles by the ligands or the *in situ* formed complexes at any of the used concentrations, as could be expected in the case of amphiphilic compounds.^[34]^ However, the alkyl substituent does show an undisputable effect on the compounds’ DNA binding, DNA cleavage as well as cytotoxic and antimicrobial properties.

The investigation of the DNA binding affinity of the ligands and complexes showed that an increase in the length of the aliphatic substituent caused a decrease in their DNA binding constant and a loss of the intercalative ability in favor of groove binding. In the case of the three alkylated derivatives these results correlated with the self‐activated DNA cleaving properties of the *in situ* formed Cu(II) complexes. The complex **CuC0**, missing the hydrophobic substituent, led to DNA aggregation at higher concentrations, affecting its DNA cleaving activity. This property has not been reported in the literature before and is possibly related to the Cu(II)‐mediated formation of a less stable π‐radical cation (in comparison to the alkylated derivatives), which leads to substrate polymerization.

The role of the hydrophobic alkyl substituent on the biological activity of the prodigiosenes was evidenced by the comparably low dark cytotoxicity of **C0** and **CuC0**. A general increase in cytotoxicity through Cu(II) complexation is in accordance with the results obtained from the DNA cleavage experiments. Whereas an increase in ROS production could be made responsible for enhanced DNA cleavage, in cells, ROS production was not that much affected by Cu(II) addition. This indicates that other mechanisms might also be involved in the cytotoxic activity.

The unprecedented cytotoxicity study of *in situ* formed Cu(II) complexes of the prodigiosenes furthermore revealed high toxicity towards three different tumor cell lines. The pronounced dark cytotoxicity of the alkylated derivatives restrains their potential to be effective as photosensitizers in PDT. The complex **CuC0**, however, not only retains a comparably low cytotoxicity towards the (non‐tumor) L929 murine fibroblast and CCD 841 CoN human epithelial cells, but also exhibits notable photoinduced toxicity against all the tested tumor cell lines. These results strongly emphasize the potential that prodigiosenes and their Cu(II) complexes hold to play a significant role in cancer treatment and PDT.

Furthermore, **CuC0** and **CuC6** as well as the ligands alone showed antibacterial activity against *S. aureus* strains. Further studies are needed to investigate the potential of the prodigiosenes as antimicrobial agents (also in the context of PDT) in human and/or veterinary medicine or as biocides.

## Experimental section

Experimental details can be found in the Supporting Information.

## Conflict of interest

The authors declare no conflict of interest.

1

## Supporting information

As a service to our authors and readers, this journal provides supporting information supplied by the authors. Such materials are peer reviewed and may be re‐organized for online delivery, but are not copy‐edited or typeset. Technical support issues arising from supporting information (other than missing files) should be addressed to the authors.

Supporting Information

## Data Availability

The data that support the findings of this study are available from the corresponding author upon reasonable request.

## Footnotes

n1 Due to low solubility, all ligands were dissolved in DMSO, with a final concentration in the incubation mixture lower than 0.4 % (56 mM). The scavenger experiments were performed using a considerably higher concentration (400 mM).)
