# Supplementary material for: Investigating Alkylated Prodigiosenes and Their Cu(II)‐Dependent Biological Activity: Interactions with DNA, Antimicrobial and Photoinduced Anticancer Activity
Source: ChemMedChem. 2021 Dec 22;17(3):e202100702. doi: 10.1002/cmdc.202100702 (PMC9306646; doi:10.1002/cmdc.202100702)
Supplement: Supplementary file 1 — Supporting Information [file CMDC-17-0-s001.pdf]

ChemMedChem

Supporting Information

Investigating Alkylated Prodigiosenes and Their Cu(II)-Dependent Biological Activity: Interactions with DNA, Antimicrobial and Photoinduced Anticancer Activity

Sebastian Doniz Kettenmann, Matthew White, Julien Colard-Thomas, Matilda Kraft, Andrea T. Feßler, Karin Danz, Gerhard Wieland, Sylvia Wagner, Stefan Schwarz, Arno Wiehe, and Nora Kulak*

S-1 Materials and general methods
S-2 Synthesis of ligands
S-3 Characterization of precursors, ligands and complexes
S-3.1 Spectra of precursors
S-3.2 NMR spectra of prodigiosenes
S-3.3 ESI mass spectra of prodigiosenes
S-3.4 HPLC data of prodigiosenes
S-3.5 UV/VIS titration experiments of complexes
S-3.6 ESI mass spectra of complexes
S-4 CD spectra
S-5 DNA melting curves
S-6 Ethidium bromide displacement studies
S-7 Fluorescence titration experiments
S-8 DNA cleavage
S-9 Cytotoxicity
S-10 ROS assay in cells
S-11 Antimicrobial susceptibility testing
References

S-1 Material and general methods

All reagents were purchased from commercial sources and were used without further purification unless otherwise stated. When noted so, the used solvents were degassed by the "freeze-pump-thaw" technique. ^{1}H and ^{13}C NMR spectra were recorded at room temperature on a JeolECX 400 (400 MHz), a JeolECZ 400 (400 MHz) or a Bruker AVANCE III 700 (700 MHz) spectrometer. The chemical shifts (δ) are given in parts per million (ppm) and coupling constants (*J*) are reported in Hz. Tetramethylsilane (δ [1H] = 0.00 ppm) was used as an external standard. For the internal standards the solvent signal of $CDCl_3$ (δ[1H] = 7.26 ppm, [13C] = 77.16 ppm) or CD_3OD (δ[1H] = 3.31 ppm, [13C] = 49.00 ppm) were used. Electrospray ionization mass spectrometry (ESI-MS) was performed on an Agilent 6210 ESI-TOF (flow rate 10 µL/min) using methanol or acetonitrile, as specified. UV–VIS measurements were carried out using 1 cm path length Hellma cuvettes with 0.5 or 1 mL sample volume on a Cary 100 Bio UV–VIS spectrophotometer (Agilent). Fluorescence emission spectra were collected using 1 cm path length Hellma fluorescence cuvettes with 1 mL sample volume on a Cary Eclipse fluorescence spectrophotometer (Varian). pH during titration experiments was adjusted using 10 mM MOPS buffer (with 100 mM NaCl, pH 7.4) or 10 mM potassium *tert*-butoxide/acetic acid (pH 10)[1], as noted on the respective measurement. Flash chromatography was performed on silica gel (40-63 µm) or basic aluminum oxide (activity grade I, 50–200 µm) as specified in the respective procedure. RP-HPLC (Chromaster 5000, VWR, Hitatchi) was carried out over a C18 column (semi-preparative: 10 µm, 10 x 250 mm, Merck; analytical: 5 µm, 3 x 250 mm, Merck) with a mobile phase consisting of deionized water and methanol (1 mM HCl, ratio specified in the respective procedure). Stock solutions of the purified ligands for the use in the biological studies were prepared in DMSO. Final maximal concentrations of DMSO are specified under the respective experiment procedure. Reference measurements were performed with maximal DMSO concentrations to ensure no influence on the experiment outcome.

UV/VIS melting curves:

CT-DNA (50 µM) melting curves were measured in buffered solution (10 mM MOPS, 0.05% DMSO, pH 7.4) in presence of the ligands or *in situ* formed Cu(II) complexes (2.5 µM) using a Cary 100 Bio UV/Vis spectrophotometer at 260 nm (heating rate 0.5 °C/min). Melting curves were normalized and used for the calculation of the melting temperatures.

Ethidium bromide (EB) displacement studies:

The fluorescence emission spectra of intercalated EB were recorded using a Cary Eclipse fluorescence spectrophotometer (Varian). A solution of CT-DNA (20 µM) and EB (1.3 µM) in buffered aqueous solution (10 mM MOPS, 100 mM NaCl, pH 7.4) was treated with increasing concentrations of the ligands in presence or absence of equimolar concentration of copper acetate. The fluorescence spectra were collected after each addition between 540 and 730 nm using an excitation wavelength of 518 nm (photomultiplier voltage: 1000 V). Final DMSO concentrations remained below 0.4%.

Circular dichroism (CD) spectroscopy:

CD spectra of CT-DNA (100 μM) in buffered aqueous solution (10 mM MOPS, 100 mM NaCl, pH 7.4) were collected on a Jasco J-810 spectrometer in the range of 220 to 320 nm with a measuring velocity of 100 nm/min, and a data point interval of 0.1 nm. Increasing concentrations of the ligands or the *in situ* formed Cu(II) complexes were used. Final DMSO concentrations remained below 0.4%.

DNA cleavage studies:

Plasmid DNA pBR322 (0.025 μg μL^{-1}) was incubated with the prodigiosenes in presence and absence of equimolar concentrations of copper acetate (5–150 μM) for 1 h at 37 °C in MOPS buffer (10 mM, 100 mM NaCl, pH 7.4). The final reaction volume was adjusted to 8 μL by adding deionized water with a final DMSO concentration below 0.8%. After incubation 1.5 μL of loading buffer containing 3.7 mM bromophenol blue and 1.2 M saccharose was added to the incubation solution and loaded onto an agarose gel (1% in 0.5X Tris-borate-EDTA (TBE) buffer) containing EB (0.2 μg mL^{-1}). Electrophoresis was carried out with an electrophoresis unit (Carl Roth; power supply: consort EV243) for 2 h at 40 V. DNA bands were visualized by fluorescence imaging of intercalated EB using a Bio-Rad GelDoc EZ Imager. Data analysis was performed with Bio-Rad's Image Lab Software (Version 3.0). Due to the decreased affinity of ethidium bromide to supercoiled DNA a correction factor of 1.22 was used.[2] In order to ensure reproducibility, all DNA cleavage experiments were performed in triplicate, and the standard deviation was used to calculate the error bars.

Detection of reactive oxygen species (ROS) by quenching of DNA cleavage:

The general DNA cleaving procedure as described before was applied, using a fixed concentration of the *in situ* formed Cu(II) complexes of 70 μM. The DMSO concentration before further addition of DMSO as scavenger was 0.4% (56 mM). Subsequently one of the following ROS scavengers was added to the incubation solution: DMSO (additional 400 mM), NaN_3 (10 mM), pyruvate (2.5 mM) or superoxide dismutase (625 U/mL). One lane containing both, pyruvate and SOD, was also included.

***In situ* formation of the copper(II) complexes for the cell culture experiments:**

For the *in situ* synthesis of the copper complexes a stock solution (2 mM) of the respective prodigiosene in DMSO was mixed with an equimolar amount of the copper(II) acetate solution in deionized water and diluted with the medium used in the cell culture experiments to the desired final concentrations (2 μM or 10 μM).

Cell viability assays:

Human epidermoid carcinoma A253 cells, human oral adenosquamous carcinoma CAL27 cells, colorectal adenocarcinoma HT29 cells, and mouse fibroblast L929 cells were grown in Dulbecco's modified eagle medium (DMEM) with 10% heat inactivated FCS, 1% penicillin (10,000 IU) and streptomycin (10,000 μg

mL^{-1}). DMEM (without phenol red) with 10% FCS was used for dilution to reach 2 or 10 µM concentration of the respective compound to be tested. In microplates 2 × 10^4 cells per well were seeded in fresh medium (DMEM without phenol red) containing 10% FCS with 2 µM or 10 µM of the compound and incubated for 24 h. After exchange of medium (to remove any compound not taken up by the cells), the photosensitization was performed at RT with a white light source (Zeiss) for 40 s at a dose rate of approx. 50 J cm^{-2}. After 24 h recovery, the cell viability of the samples was assessed using the XTT assay[3] and the absorbance was measured with a Tecan Infinite 200 microplate reader, at a wavelength of 490 nm. A wavelength of 690 nm was used to measure the reference absorbance.

For a comparison between cancer cells and healthy cells, both of human epithelial origin in the colon, HT29 cells were grown in DMEM with 10 % FCS, 1% penicillin (10,000 IU) and streptomycin (10,000 µg mL^{-1}), and CCD 841 CoN cells (ATCC CRL-1790) were grown in MEM with 10 % FCS and 1% penicillin (10,000 IU) and streptomycin (10,000 µg mL^{-1}). 2 × 10^4 HT29 cells or 8.5 × 10^3 CCD 841 CoN cells were seeded in black microwell plates (greiner bio-one). Compounds to be tested were diluted to 2 µM or 10 µM in the respective culture media, added to the plates 24 h after seeding and incubated for 24 h. Medium was exchanged at the end of the incubation time to remove any compound not taken up by the cells and photosensitization was performed at RT with a white light LED source (19 mW) for 43 min at a dose rate of approx. 50 J cm^{-2}. After 24 h recovery the cell viability was assessed using the XTT assay.

ROS assay in cells:

1.1875 × 10^5 HT29 cells per well or 5.05 × 10^4 CCD 841 CoN cells per well were seeded in 24 well plates. Compounds to be tested were diluted to 10 µM in the respective culture media, added to the plates 24 h after seeding and incubated for 1 h. Medium was exchanged at the end of the incubation time to remove any compound not taken up by the cells and photosensitization was performed immediately at RT with a white light LED source (19 mW) at a dose rate of approx. 50 J cm^{-2}. Directly after the photosensitization, 5 µM CellROX® Orange was added to the cells and incubated for 30 min. Cells were washed twice with PBS, removed from the plates, collected in round bottom tubes, resuspended in 0.5 mL BD FACSFlow™ and fluorescence measured with BD FACSCalibur™. Results were analyzed using the FlowJo software.

Antimicrobial Susceptibility testing (AST):

The antimicrobial susceptibility of **C0**, **C6** and **C16** as well as their copper complexes **CuC0**, **CuC6** and **CuC16** was investigated according to the standards of the Clinical and Laboratory Standards Institute.[4,5] For the antimicrobial susceptibility testing two-fold dilution series of the prodigiosenes were prepared in 50 µL cation-adjusted Mueller-Hinton broth (CAMHB) in the double concentration of the test concentration required in the rows 1 to 10 of the microtiter plates. Row 11 was used as growth control and was filled with 50 µL of CAMHB and row 12 was used as sterility control and filled with 100 µL of CAMHB. For the first investigation of the antimicrobial activity six reference strains commonly used for AST and biocide efficacy

testing were used, including three Gram-negative (*Escherichia coli* ATCC® 25922, *E. coli* ATCC® 10536, *Pseudomonas aeruginosa* ATCC® 15442) and three Gram-positive (*Staphylococcus aureus* ATCC® 29213 and *S. aureus* ATCC® 6538, *Enterococcus hirae* ATCC® 10541) isolates. For the testing of *Enterococcus hirae* ATCC® 10541 2 % lysed horse blood was added to the CAMHB medium. In addition, 15 additional staphylococcal field Isolates (Table S-11) were tested for their susceptibility against **C6** and **CuC6** as well as **C16** and **CuC16**.

S-2 Synthesis of ligands

Ligands were synthesized based on procedures adapted from the literature[6] (Scheme S-1). Modifications and peculiarities of the syntheses are described in the following. The mono-alkylated pyrroles **3**–**5** were prepared from 1*H*-pyrrole **2** and the corresponding alkyl bromide, as it is described in the literature procedure.[7] The carboxaldehydes **6**–**9** were thereupon easily accessible *via* a Vilsmeier-Haack-formylation. It is to be noted that, especially in the case of the hexadecyl derivative **9**, the treatment with saturated Na_2CO_3 solution during workup was insufficient to completely hydrolyze the intermediately generated dimethyl iminium salt **9b**. The air stable iminium salt could, however, be further hydrolyzed to the carboxaldehyde **9** through treatment with saturated NaOAc solution overnight. The condensation reaction between the carboxaldehydes **6**–**9** and 4-methoxy-3-pyrrolin-2-one under basic conditions yielded the dipyrrinones **10**–**13**. Further treatment of **10**-**13** with trifluoromethanesulfonic anhydride gave the triflates **14**–**17**, which can be used in the reaction with 1-*N*-Boc-pyrrole-2-boronic acid **18** under Suzuki-Miyaura cross-coupling conditions.

Our attempts to synthesize and isolate the boronic acid **18** following the procedure established in the literature,[8] and subsequently using it in the cross-coupling reaction only afforded the desired product in low yields (29%). We attribute the low yield in part to the instability of the pyrrole boronic acid **18**, which is known to be prone to protodeboronation,[9–12] but also to the low thermal stability of the triflates **14**–**17** under the reaction conditions (90 °C). The thermal decomposition of **14**–**17** into a complex product mixture can be observed when exposing the triflates to the coupling conditions in absence of the boronic acid. The use of the MIDA-boronate, an air-stable precursor of the boronic acid as described by Burke et al.,[8] did not improve the overall yield of the coupling step (14–24%). In our experience, an improvement of the yield for the cross-coupling reaction was only achieved by using a larger excess of the boronic acid (8–10 eq.) and when performing the synthesis of the boronate and the ensuing cross–coupling in a one-pot fashion at room temperature, without isolation of the sensitive boronic acid. Final Boc-deprotection occurs simultaneously during purification of the desired prodigiosene due to basic conditions. The different reaction conditions used for the cross-coupling step are described in the following section. This section also includes the NMR spectroscopic characterization of precursors **9b**, **10**, **11**, **13**, **14**, **15**, and **17**, which as such are not described in the literature.

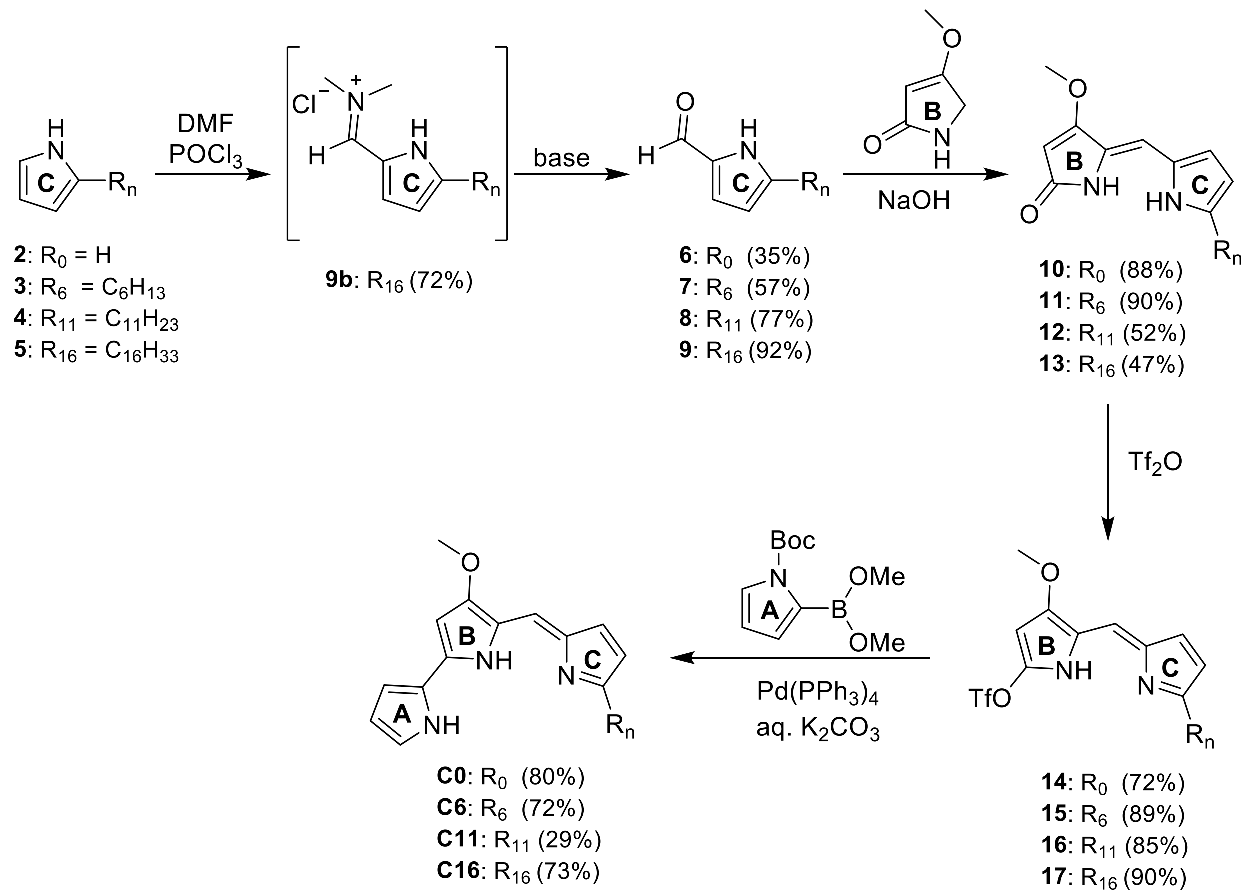

Scheme S-1. Synthesis pathway used for the preparation of prodigiosenes **C0**, **C6**, **C11**, and **C16**.

***N*-((5-Hexadecyl-1*H*-pyrrol-2-yl)methylene)-*N*-methylmethaneiminium chloride (9b)**

Phosphoryl chloride (34.8 mL, 372 mmol) was added to DMF (28.6 mL, 372 mmol) at 0 °C under argon atmosphere. After 30 min a solution of 2-hexadecylpyrrole **5** (17.78 g, 61.0 mmol) in DMF (50 mL) was added to the resulting white solid while maintaining the mixture at 0 °C. The mixture was stirred for 15 h at r.t., and subsequently treated slowly with saturated sodium bicarbonate solution (200 mL) at 0 °C. After extraction with DCM (4 x 100 mL), the combined organic layers were washed with brine (2 x 100 mL), dried over sodium sulfate, and concentrated under reduced pressure to yield **9b** as a white solid (16.9 g, 72%).

^{1}H NMR ($CDCl_3$, 400 MHz): δ = 0.75 (t, *J* = 6.9 Hz, 3H), 1.05-1.28 (m, 26H), 1.62 (p, *J* = 7.6 Hz, 2H), 2.70 (t, *J* = 7.7 Hz, 2H), 3.42 (s, 3H), 3.55 (s, 3H), 6.29 (dd, *J* = 4.4, 1.9 Hz, 1H), 7.05 (dd, *J* = 4.4, 1.9 Hz, 1H), 9.03 (s, 1H), 13.84 (s, 1H) ppm.

^{13}C NMR ($CDCl_3$, 100 MHz): δ = 14.02, 22.57, 28.05, 28.43, 29.16, 29.19, 29.24, 29.43, 29.51, 29.54, 29.57, 29.57, 29.58, 29.59, 31.80, 42.00, 49.18, 115.6, 121.8, 124.6, 151.9, 152.4 ppm.

HRMS (ESI): calcd. for $[M-Cl]^+$ 347.3421, found 347.3428.

5-Hexadecylpyrrole-2-carboxaldehyde (9)

The intermediate product **9b** (1.00 g, 2.61 mmol) was dissolved in methanol (20 mL), treated with 5 mL saturated sodium acetate solution and stirred over night at r.t. After extraction with DCM (3 x 30 mL), the combined organic layers were washed with brine (50 mL), dried over sodium sulfate, and concentrated under reduced pressure to yield **9** as a beige solid (852 mg, 92%).

^{1}H NMR ($CDCl_3$, 400 MHz): δ = 0.87 (t, *J* = 6.9 Hz, 3H), 1.20-1.38 (m, 26H), 1.64 (p, *J* = 7.4 Hz, 2H), 2.63 (t, *J* = 7.8 Hz, 2H), 6.07 (dd, *J* = 3.4, 2.9 Hz, 1H), 6.88 (dd, *J* = 3.8, 2.6 Hz, 1H), 9.00 (s, 1H), 9.38 (s, 1H) ppm. These data are consistent with the ones reported in the literature.[13]

4-Methoxy-5-(1*H*-pyrrol-2-ylmethylene)-1,5-dihydropyrrol-2-one (10)

Compound **10** was synthesized following the procedures for **11** as described in the literature.[6]

^{1}H NMR ($CDCl_3$, 400 MHz): δ = 3.91 (s, 3H), 5.20 (s, 1H), 6.28 (ddd, *J* = 4.7, 2.7, 2.1 Hz, 1H) 6.38 (s, 1H) 6.46 (m, 1H), 7.06 (ddd, *J* = 2.7, 1.4, 0.7 Hz, 1H), 10.35 (s, 1H), 10.43 (s, 1H). These data are consistent with the ones for the structurally similar undecyl analog **12** reported in the literature.[6]

4-Methoxy-5-(5-hexyl-1*H*-pyrrol-2-yl-methylene)-1,5-dihydro-pyrrol-2-one (11)

Compound **11** was synthesized following the procedures for **12** as described in the literature.[6]

^{1}H NMR ($CDCl_3$, 400 MHz): δ = 0.89 (t, *J* = 7.0 Hz, 3H), 1.27-1.44 (m, 6H), 1.72 (p, *J* = 7.5 Hz, 2H), 2.73 (t, *J* = 7.8 Hz, 2H), 3.89 (s, 3H), 5.09 (d, *J* = 1.6 Hz, 1H), 5.97 (dd, *J* = 3.5, 2.1 Hz, 1H), 6.31 (s, 1H), 6.36 (t, *J* = 3.1 Hz, 1H), 10.26 (s, 1H), 10.76 (s, 1H) ppm. These data are consistent with the ones for the structurally similar undecyl analog **12** reported in the literature.[4]

4-Methoxy-5-(5-undecyl-1*H*-pyrrol-2-yl-methylene)-1,5-dihydro-pyrrol-2-one (13)

Compound **13** was synthesized following the procedures for **12** as described in the literature.[6]

^{1}H NMR ($CDCl_3$, 400 MHz): δ = 0.88 (t, *J* = 6.8 Hz, 3H), 1.22-1.44 (m, 26H), 1.72 (p, *J* = 7.7 Hz, 2H), 2.73 (t, *J* = 7.7 Hz, 2H), 3.89 (s, 3H), 5.08 (d, *J* = 1.6 Hz, 1H), 5.97 (dd, *J* = 3.5, 2.0 Hz, 1H), 6.32 (s, 1H), 6.36 (t, *J* = 3.1 Hz, 1H), 10.31 (s, 1H), 10.82 (s, 1H) ppm. These data are consistent with the one for the structurally similar undecyl analog **12** reported in the literature.[6]

2-Trifluoromethanesulfonyloxy-4-methoxy-5-(2*H*-pyrrol-2-ylidine)-1*H*-pyrrole (14)

Compound **14** was synthesized following the procedures for **16** as described in the literature.[6]

^{1}H NMR (CDCl$_3$, 400 MHz): δ = 3.89 (s, 3H), 5.43 (s, 1H) 6.29 (ddd, *J* = 3.7, 2.5, 2.5 Hz, 1H), 6.70 (ddd, *J* = 3.7, 2.2, 1.3 Hz, 1H) 7.08 (s, 1H), 7.16 (m, 1H), 10.90 (s, 1H) ppm. These data are consistent with the ones for the structurally similar undecyl analog **16** reported in the literature.[6]

2-Trifluoromethanesulfonyloxy-4-methoxy-5-[(5-hexyl-2*H*-pyrrol-2-ylidene)methyl]-1*H*-pyrrole (15)

Compound **15** was synthesized following the procedures for **16** as described in the literature. [6]

^{1}H NMR (CDCl$_3$, 400 MHz): δ = 0.90 (t, *J* = 6.9 Hz, 3H), 1.27-1.42 (m, 6H), 1.67 (p, *J* = 7.6 Hz, 2H), 2.67 (t, *J* = 7.7 Hz, 2H), 3.87 (s, 3H), 5.40 (s, 1H), 6.04 (d, *J* = 3.8 Hz, 1H), 6.65 (d, *J* = 3.8 Hz, 1H), 7.02 (s, 1H), 10.81 (s, 1H) ppm. These data are consistent with the ones for the structurally similar undecyl analog **16** reported in the literature.[6]

2-Trifluoromethanesulfonyloxy-4-methoxy-5-[(5-hexadecyl-2*H*-pyrrol-2-ylidene)methyl]-1*H*-pyrrole (17)

Compound **17** was synthesized following the procedures for **16** as described in the literature.[6]

^{1}H NMR (CDCl$_3$, 400 MHz): δ = 0.88 (t, *J* = 6.9 Hz, 3H), 1.21-1.43 (m, 26H), 1.67 (p, *J* = 7.2 Hz, 2H), 2.67 (t, *J* = 7.7 Hz, 2H), 3.87 (s, 3H), 5.40 (s, 1H), 6.04 (d, *J* = 3.9 Hz, 1H), 6.65 (d, *J* = 3.9 Hz, 1H), 7.02 (s, 1H), 10.82 (s, 1H) ppm. These data are consistent with the ones for the structurally similar undecyl analog **16** reported in the literature.[6]

Synthesis of undecylprodigiosin C11 and hexylprodigiosin C6 through Suzuki–Miyaura cross-coupling with MIDA boronate

For this procedure we used the *N*-Boc-pyrrole-2-boronic acid MIDA ester that functions as an air stable precursor of the free boronic acid as reported by Bruke et al.[10] A solution of triflate **16** (240 mg, 0.50 mmol), *N*-Boc-pyrrole-2-boronic acid MIDA ester (161 mg, 0.50 mmol), SPhos (21 mg, 10 mol%) and palladium(**II**) acetate (6 mg, 5 mol%) in degassed 1,4-dioxane (7 mL) was prepared under argon atmosphere. The mixture was stirred at r.t. for 10 min., 1.4 mL of a degassed aqueous solution of tripotassium phosphate (791 mg, 3.75 mmol) was added, and the resulting solution was stirred at 60 °C for 6 h. The mixture was cooled to r.t. and treated with 1 M sodium hydroxide (10 mL). After extraction with diethyl ether, the combined organic layers were washed with brine, dried over sodium sulfate, and evaporated to dryness. The crude product was purified by column chromatography over aluminum oxide eluting with a hexane:ethyl acetate 4:1 mixture. The fraction containing the title compound was dissolved in 1–2 mL of ethanol and treated with three drops of HCl (25% in water), to yield the hydrochloric salt of **C11** as a red solid (90 mg, 14%).

Following the same procedure as for **C11**, although using **15** as a precursor and tetrakis(triphenylphosphine)palladium(0) (5 mol%) as catalyst, **C6** was obtained as a red solid (24%).

Synthesis of undecylprodigiosin C11 through Suzuki–Miyaura cross-coupling with the isolated N-Boc-pyrrole-2-boronic acid

A solution of the triflate **16** (418 mg, 0.877 mmol) in abs. degassed THF (30 mL) under argon atmosphere was treated in sequence with *N*-Boc-pyrrole-2-boronic acid (740 mg, 3.51 mmol), tetrakis(triphenylphosphine)palladium(0) (50 mg, 0.044 mmol), and potassium carbonate (969 mg, 7.02 mmol) and stirred for 6 h at 90 °C. After extraction with ethyl acetate (3 x 50 mL), the combined organic layers were washed with water (30 mL) and brine (50 mL), dried over sodium sulfate and concentrated under reduced pressure. The crude product was purified by column chromatography on basic aluminum oxide, eluting with a hexane:ethyl acetate 4:1 mixture to yield **C11** as a red solid (100 mg, 29%). Further purification of the title compound before the biological studies was achieved through precipitation of the hydrochloric salt from a solution in ethanol after the addition of three drops of HCl (25% in water), followed by purification through HPLC over a C18 column (mobile phase: ethanol:water 8:2).

Synthesis of prodigiosenes C0, C6, and C16 through a one-pot reaction with pyrrole boronate

A solution of 2,2,6,6-tetramethylpiperidine (0.34 mL g, 2.00 mmol) in abs. degassed THF (10 mL) was cooled to -78 °C under argon atmosphere. A 1.6 M solution of *n*-BuLi (1.25 mL, 2.00 mmol) in hexane was added dropwise so that the temperature of the mixture remained below -75 °C, the mixture was then stirred at -75 °C for 10 min and warmed to 0 °C within 30 min. The mixture was cooled again to -75 °C and a solution of *N*-Boc-pyrrole (0.30 mL, 1.80 mmol) in abs. degassed THF (10 mL) was added dropwise. The mixture was stirred at this temperature for 60 min. Trimethyl borate (0.30 mL, 2.70 mmol) was slowly added and the mixture was allowed to reach r.t. overnight. A solution of the triflate **15** (80 mg, 0.20 mmol) in abs. degassed THF (5 mL) under argon atmosphere was treated in sequence with tetrakis(triphenylphosphine)palladium(0) (50 mg, 0.044 mmol), potassium carbonate (969 mg, 7.02 mmol), and the solution of the pyrrole boronate. The mixture was stirred over night at r.t. After extraction with ethyl acetate (3 x 50 mL), the combined organic layers were washed with water (30 mL) and brine (50 mL), dried over sodium sulfate and concentrated under reduced pressure. The crude product was purified by column chromatography on basic aluminum oxide eluting with a hexane:ethyl acetate 4:1 mixture to yield **C6** as a deep red solid (45 mg, 70%). Further purification of the title compound before the biological studies was achieved through precipitation of the hydrochloric salt from a solution in ethanol after the addition of three drops of HCl (25% in water), followed by purification through HPLC over a C18 column (mobile phase: ethanol:water 8:2).

Following the one-pot procedure as for **C6**, although using triflate **17**, **C16** was obtained and purified by column chromatography on basic aluminum oxide eluting with a hexane:ethyl acetate 4:1 mixture. The

fraction containing the title compound was dissolved in 1 mL of ethanol and treated with three drops of HCl (25% in water) to yield the hydrochloric salt of **C16** as a deep red-violet solid (73%). Further purification of the title compound before the biological studies was achieved through HPLC over a C18-column (mobile phase: ethanol:water 95:5).

Following the one-pot procedure as for **C6**, although using triflate **14**, **C0** was obtained and purified by column chromatography on basic aluminum oxide eluting with a hexane:ethyl acetate 4:1 mixture to yield **C0** as a deep red solid (52%). Further purification of the title compound before the biological studies was achieved through HPLC over a C18 column (mobile phase: ethanol:water 6:4), followed by neutralization of the hydrochloric salt with aq. potassium carbonate solution, extraction with DCM, drying over sodium sulfate, and removal of the solvents under reduced pressure.

α-unsubstituted prodigiosin C0 (4-Methoxy-5-((2*H*-pyrrol-2-ylidene)methyl)-1*H*,1'*H*-2,2'-bipyrrole)

^{1}H NMR (CD_3OD, 400 MHz): δ = 3.92 (s, 3H), 6.10 (s, 1H), 6.24 (dd, *J* = 2.5, 3.7, 1H), 6.27 (dd, *J* = 2.5, 3.7, 1H), 6.62 (d, *J* = 3.5, 1H), 6.79 (s, 1H), 6.80 (m, 1H), 7.04 (dd, *J* = 1.3, 2.6, 1H), 7.14 (dd, *J* = 1.3, 2.6, 1H) ppm.

^{13}C NMR (CD_3OD, 100 MHz): δ = 59.07, 96.20, 111.4, 111.6, 114.4, 115.7, 119.0, 123.1, 124.2, 126.6, 129.1, 131.1, 169.9, 169.9 ppm.

HRMS (ESI): calcd. for $[M+H]^+$ 240.1137, found 240.1141.

Hexylprodigiosin C6 (4-Methoxy-5-((5-hexyl-2*H*-pyrrol-2-ylidene)methyl)-1*H*,1'*H*-2,2'-bipyrrole hydrochloric salt)

^{1}H NMR ($CDCl_3$, 700 MHz): δ = 0.88 (t, *J* = 6.9 Hz, 3H), 1.30-1.44 (m, 6H), 1.78 (p, *J* = 7.7 Hz, 2H), 2.95 (t, *J* = 7.7 Hz, 2H), 4.02 (s, 3H), 6.09 (d, *J* = 1.8 Hz, 1H), 6.21 (d, *J* = 3.6 Hz, 1H), 6.37 (m, 1H), 6.84 (dd, *J* = 1.8, 3.7 Hz, 1H), 6.95 (m, 1H), 7.01 (s, 1H), 7.25 (m, 1H), 12.69 (s, 1H), 12.74 (s, 1H), 12.93 (s, 1H) ppm. The ^{1}H NMR data for **C6** are reported in the literature,[14] but deviate significantly from our results.

^{13}C NMR ($CDCl_3$, 176 MHz): δ = 14.23, 22.73, 28.61, 29.19, 29.36, 31.72, 58.95, 93.17, 112.1, 112.7, 116.6, 118.0, 121.6, 122.3, 126.2, 127.7, 129.4, 149.0, 153.4, 166.4 ppm.

HRMS (ESI): calcd. for $[M+H]^+$ 324.2070, found 324.2073.

Undecylprodigiosin C11 (4-Methoxy-5-((5-undecyl-2*H*-pyrrol-2-ylidene)methyl)-1*H*,1'*H*-2,2'-bipyrrole hydrochloric salt)

^{1}H NMR ($CDCl_3$, 400 MHz): δ = 0.87 (t, 3H), 1.2-1.5 (m, 16H), 1.77 (m, *J* = 7.7, 2H), 2.94 (t, *J* = 7.7, 2H), 4.02 (s, 3H), 6.09 (d, *J* = 1.9, 1H), 6.21 (dd, *J* = 1.8, 3.9, 1H), 6.36 (dd, *J* = 2.1, 3.9, 1H), 6.84 (dd, *J* = 2.5, 3.9, 1H), 6.95 (m, 1H), 7.00 (s, 1H), 7.25 (m, 1H), 12.67 (s, 1H), 12.72 (s, 1H), 12.90 (s, 1H) ppm. These data are consistent with the ones reported in the literature.[6]

^{13}C NMR ($CDCl_3$, 100 MHz): δ = 14.31, 22.87, 28.59, 29.39, 29.49, 29.52, 29.56, 29.72, 29.78, 29.79, 32.06, 58.95, 93.17, 112.1, 112.7, 116.6, 118.0, 121.6, 122.3, 126.1, 127.7, 129.4, 148.9, 153.4, 166.4 ppm. The ^{13}C NMR data for **C11** that are reported in the literature[15] deviate marginally from our results.

HRMS (ESI): calcd. for $[M+H]^+$ 394.2853, found 394.2884; calcd. for $[2M+2H+Cl]^+$ 823.5400, found 823.5415.

Hexadecylprodigiosin C16 (4-Methoxy-5-((5-hexadecyl-2*H*-pyrrol-2-ylidene)methyl)-1*H*,1'*H*-2,2'-bipyrrole hydrochloric salt)

^{1}H NMR ($CDCl_3$, 700 MHz): δ = 0.87 (t, *J* = 7.1 Hz, 3H), 1.21-1.30 (m, 26H), 1.77 (p, *J* = 7.7 Hz, 2H), 2.94 (t, *J* = 7.7 Hz, 2H), 4.02 (s, 3H), 6.09 (s, 1H), 6.20 (dd, *J* = 1.8, 3.9 Hz, 1H), 6.36 (m, 1H), 6.84 (t, *J* = 3.2 Hz, 1H), 6.95 (m, 1H), 7.00 (s, 1H), 7.24 (m, 1H), 12.67 (s, 1H), 12.73 (s, 1H), 12.91 (s, 1H) ppm. These data are consistent with the ones reported in the literature.[14]

^{13}C NMR ($CDCl_3$, 176 MHz): δ =14.25, 22.82, 28.58, 29.37, 29.49, 29.51, 29.54, 29.71, 29.78, 29.79, 29.81, 29.83, 32.06, 58.93, 93.20, 112.1, 112.7, 116.6, 118.0, 121.6, 122.3, 126.1, 127.7, 129.4, 148.9, 153.4, 166.4 ppm.

HRMS (ESI): calcd. for $[M+H]^+$ 464.3635, found 464.3621.

S-3 Characterization of precursors, ligands and complexes

S-3.1 Spectra of precursors

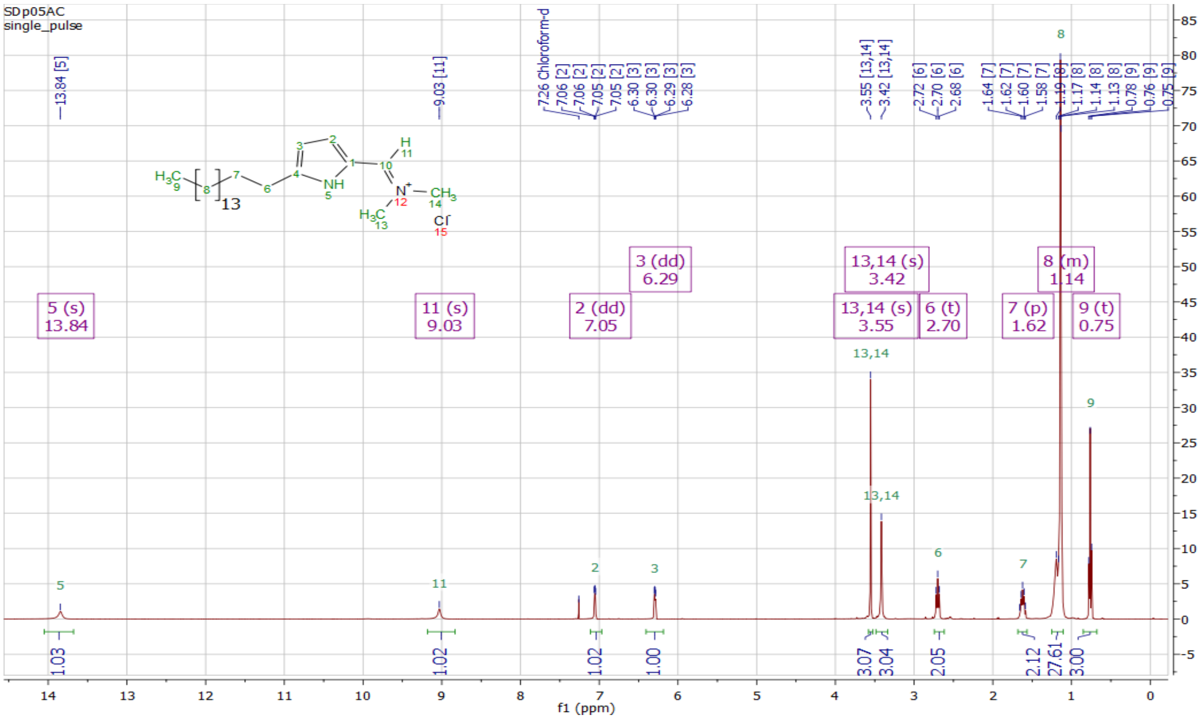

Figure S-3.1.1. ^{1}H NMR spectrum of **9b** in $CDCl_3$.

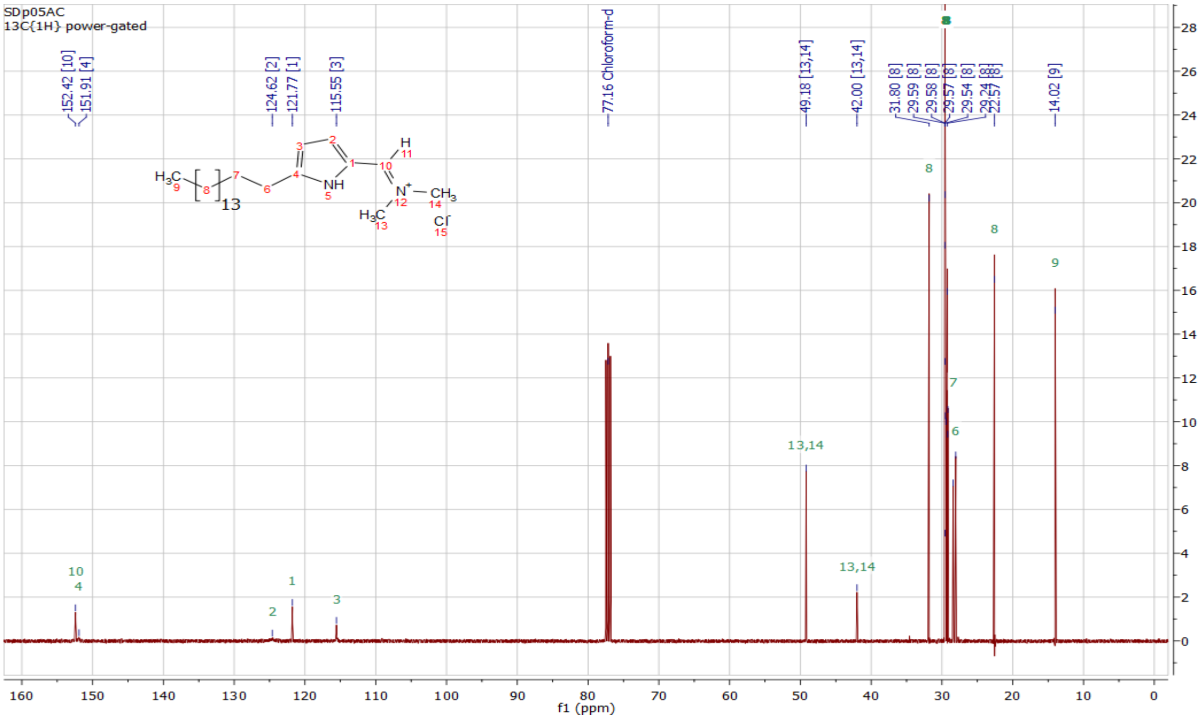

Figure S-3.1.2. ^{13}C NMR spectrum of **9b** in $CDCl_3$.

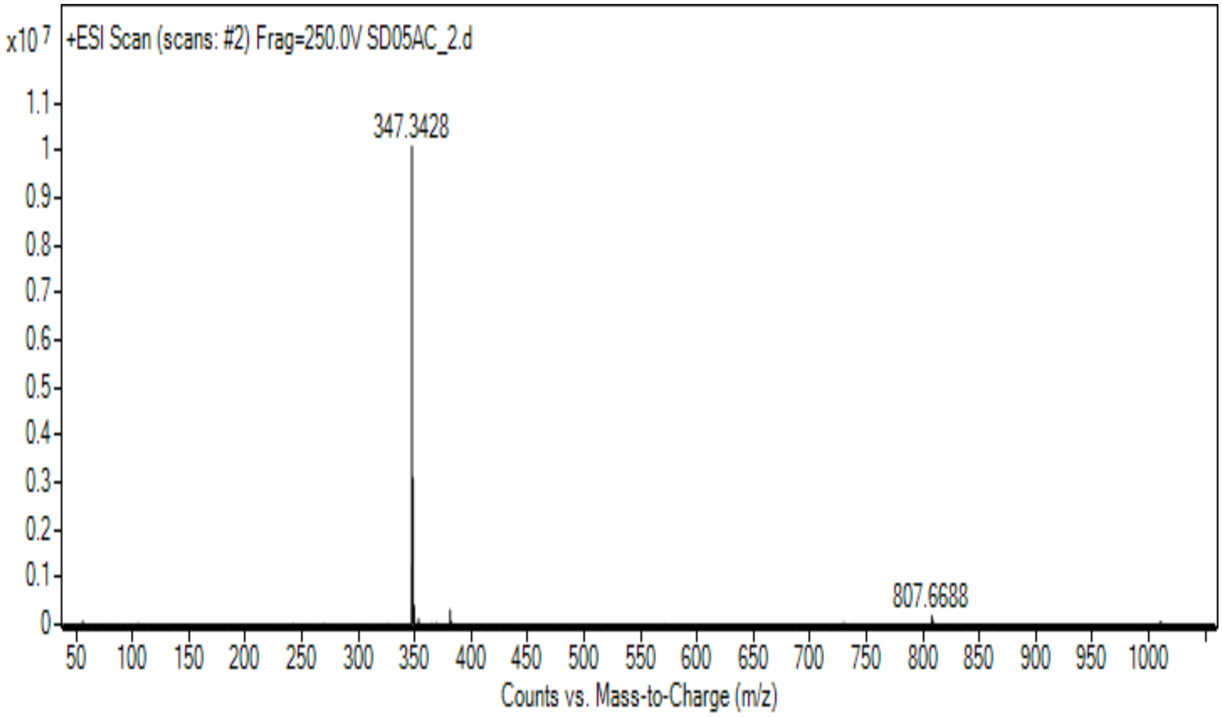

Figure S-3.1.3. ESI mass spectrum of **9b** in methanol.

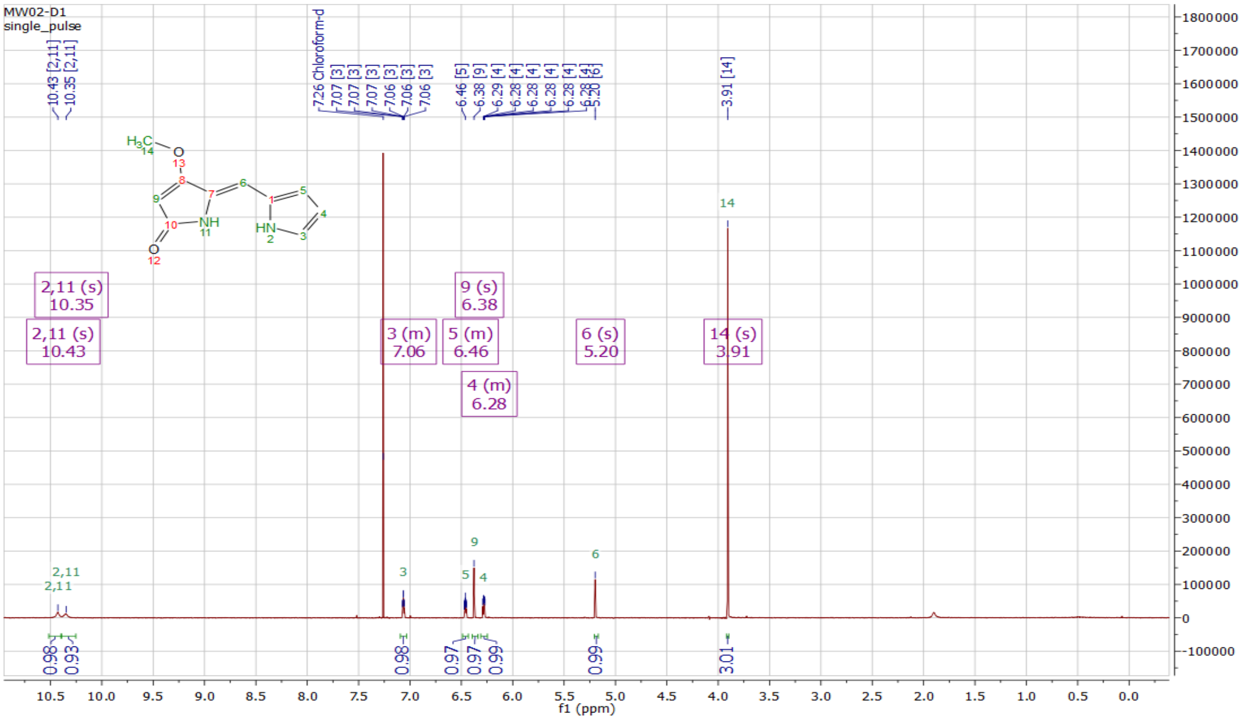

Figure S-3.1.4. ^{1}H NMR spectrum of **10** in $CDCl_3$.

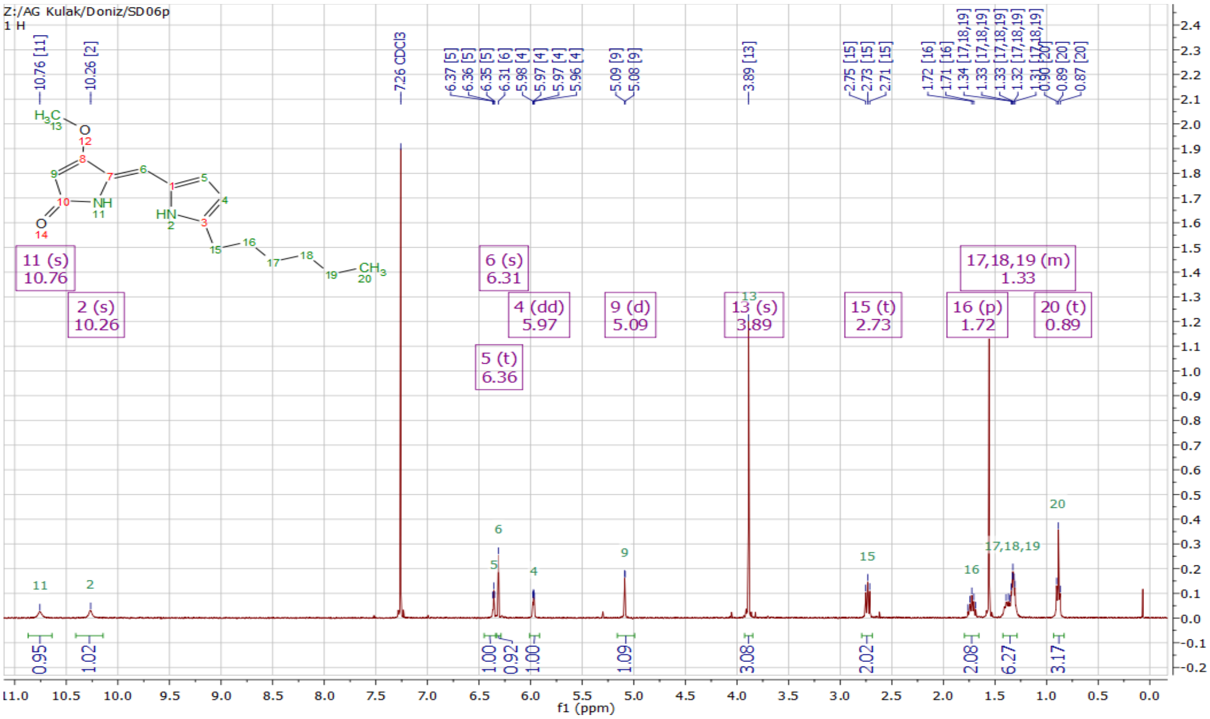

Figure S-3.1.5. ^{1}H NMR spectrum of **11** in $CDCl_3$.

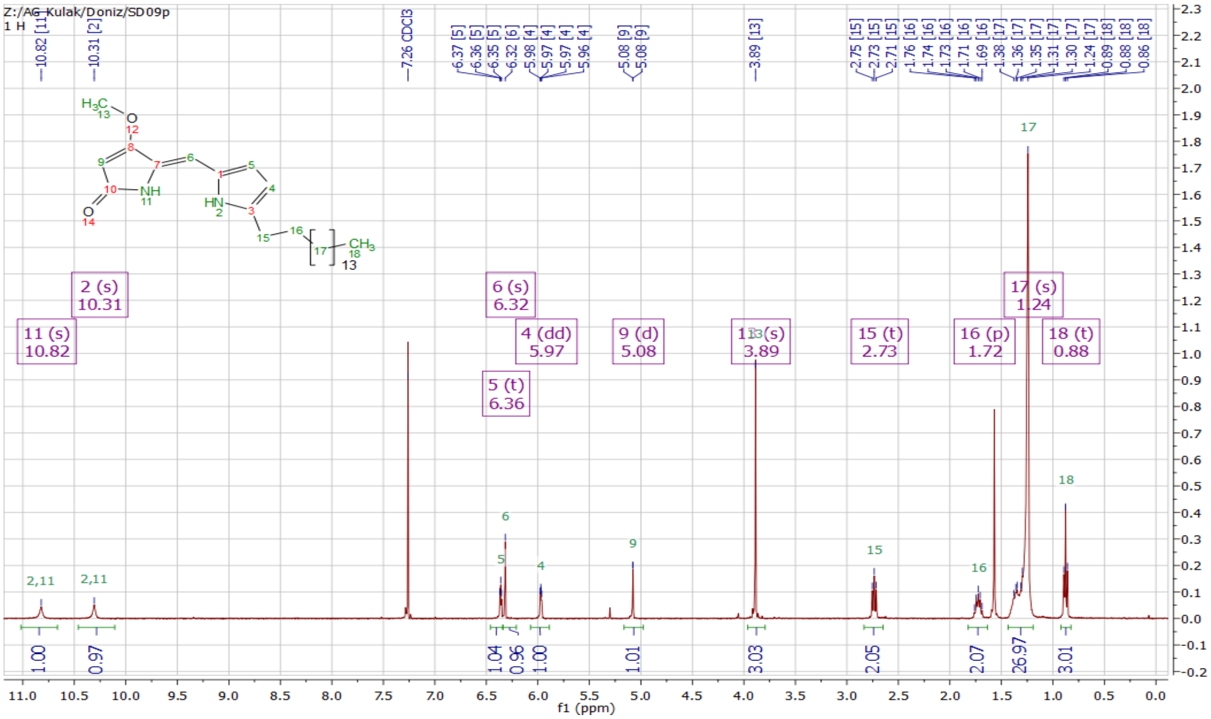

Figure S-3.1.6. ^{1}H NMR spectrum of **13** in $CDCl_3$.

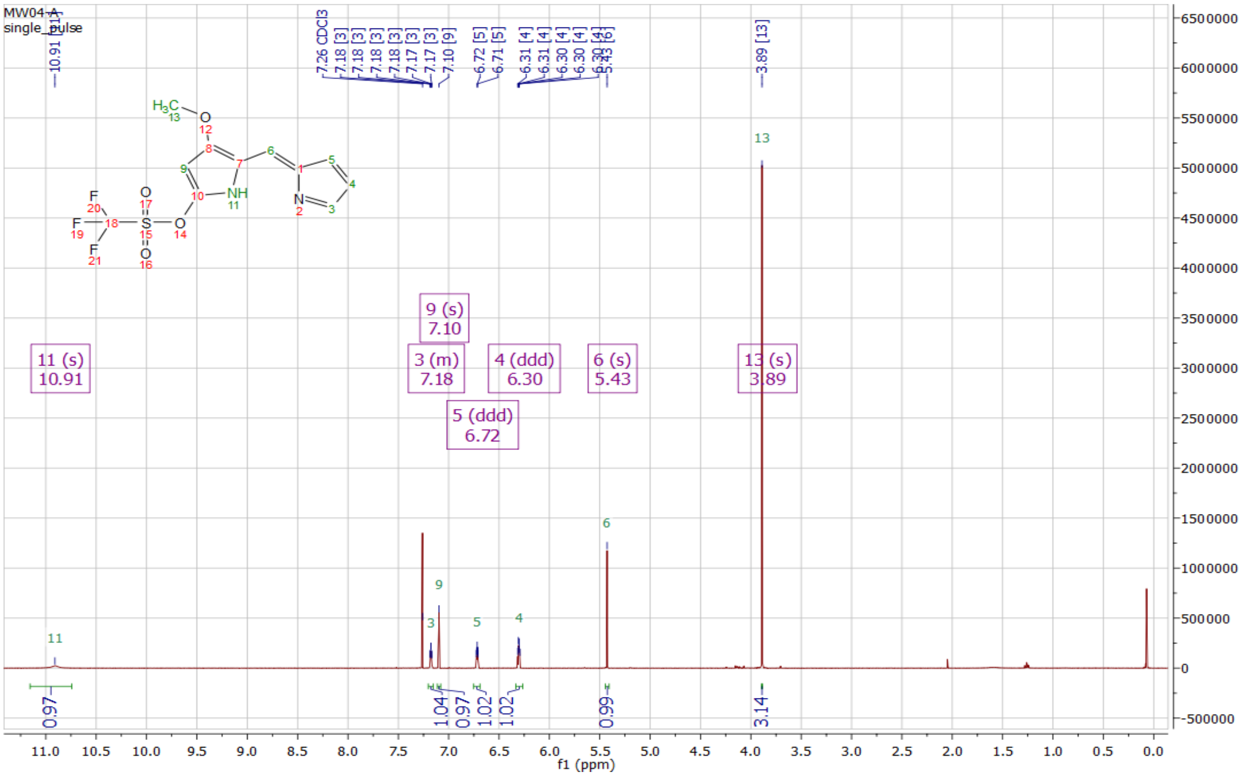

Figure S-3.1.7. ^{1}H NMR spectrum of **14** in $CDCl_3$.

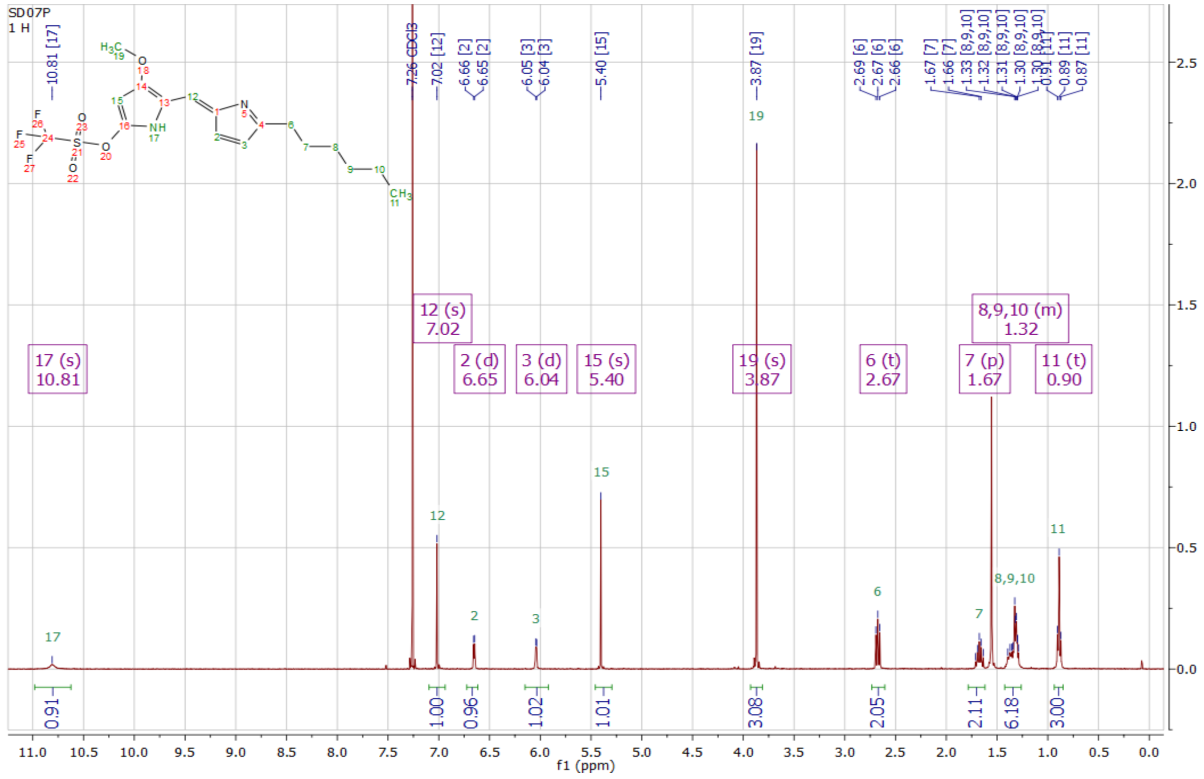

Figure S-3.1.8. ^{1}H NMR spectrum of **15** in $CDCl_3$.

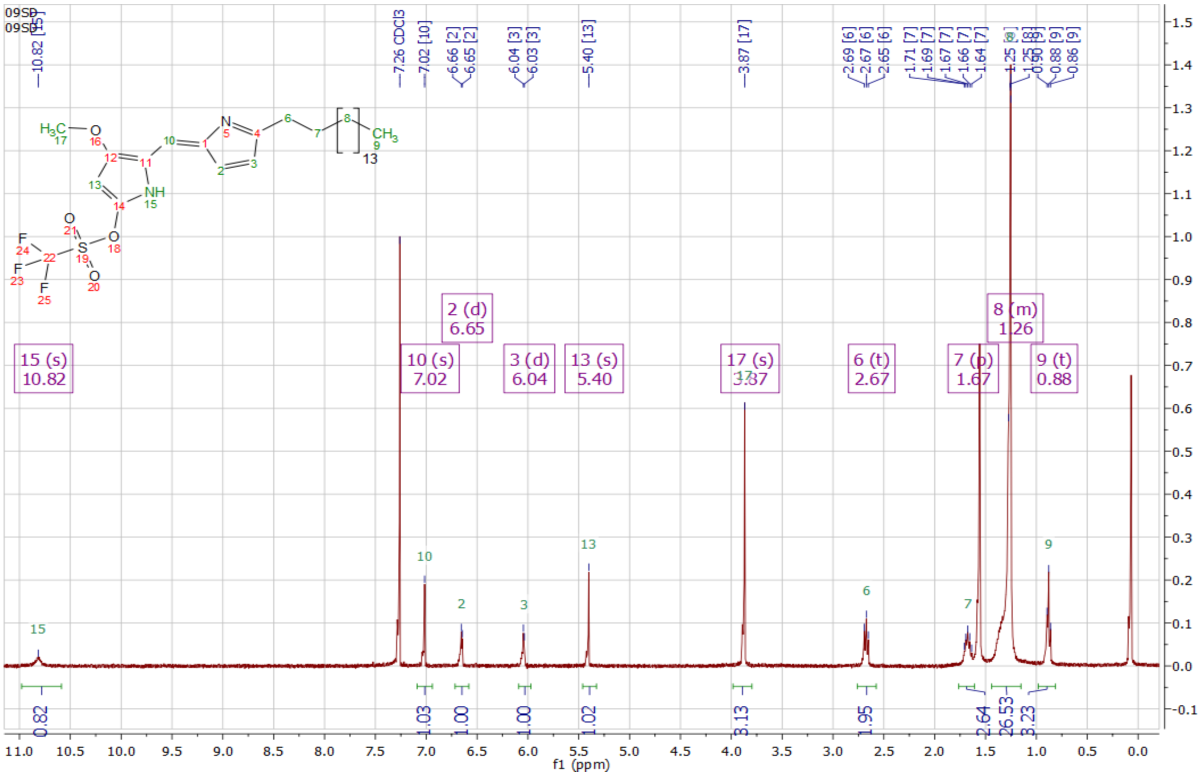

Figure S-3.1.9. ^{1}H NMR spectrum of **17** in $CDCl_3$.

S-3.2 NMR spectra of prodigiosenes

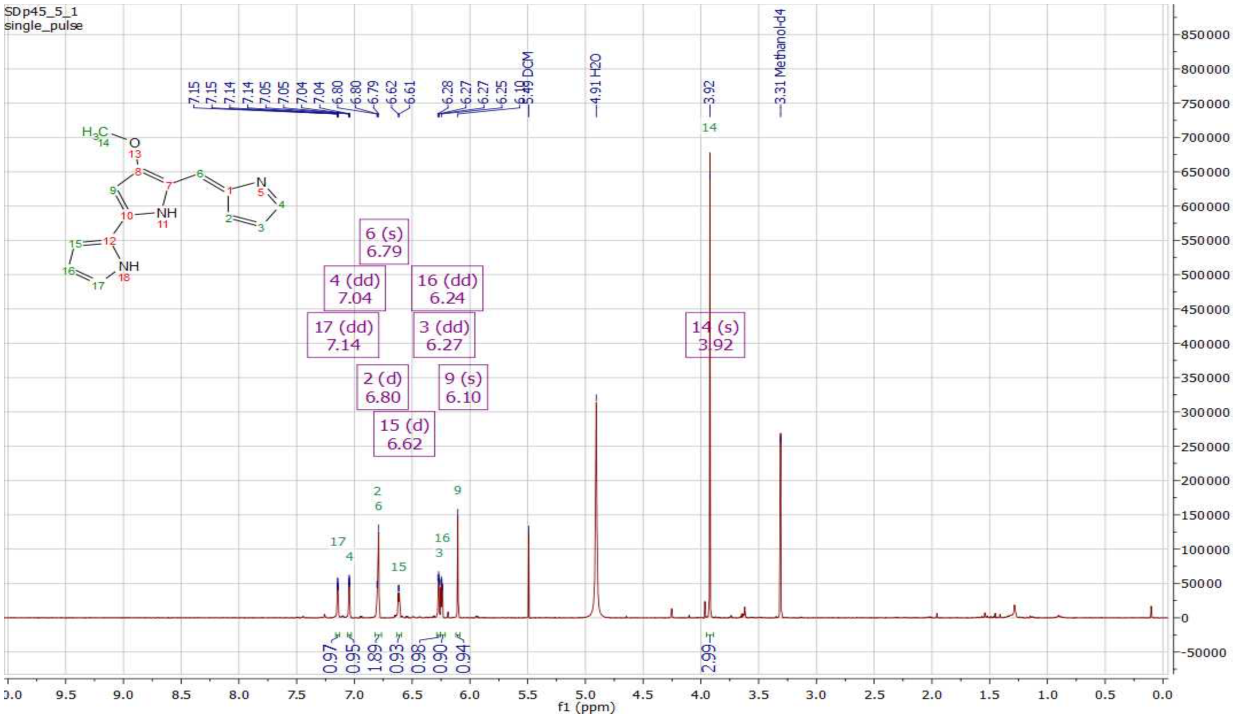

Figure S-3.2.1. ^{1}H NMR spectrum of **C0** in CD_3OD.

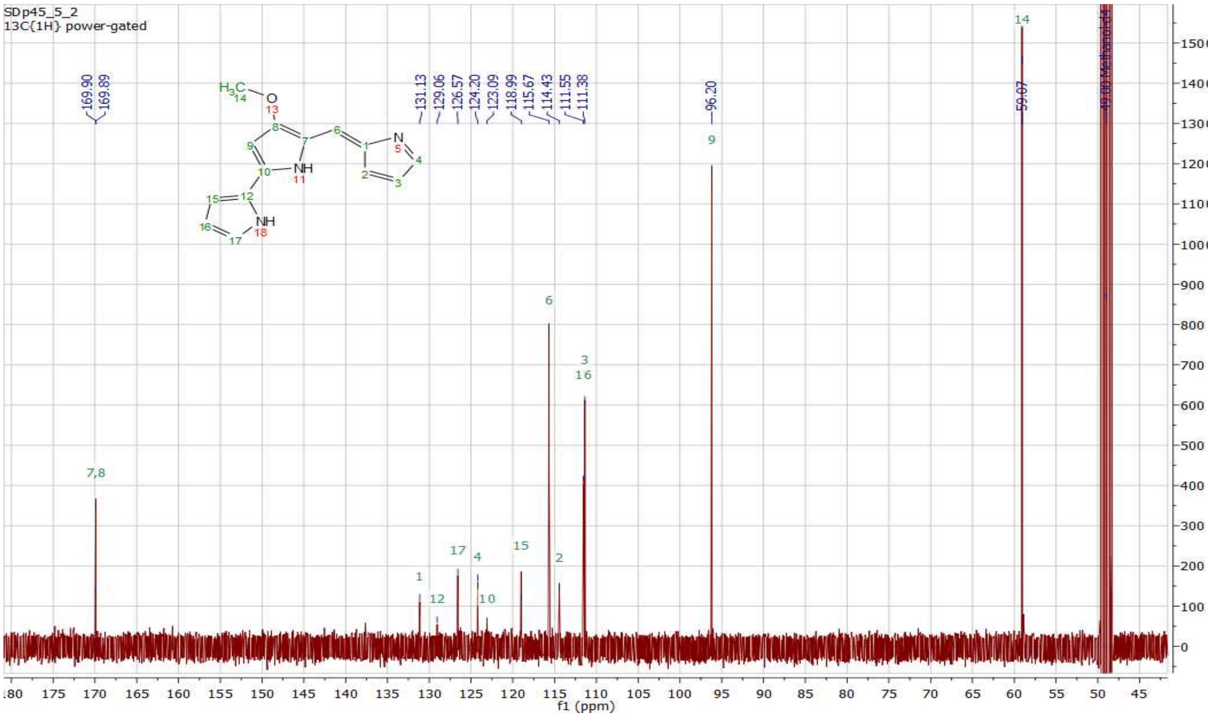

Figure S-3.2.2. ^{13}C NMR spectrum of **C0** in CD_3OD.

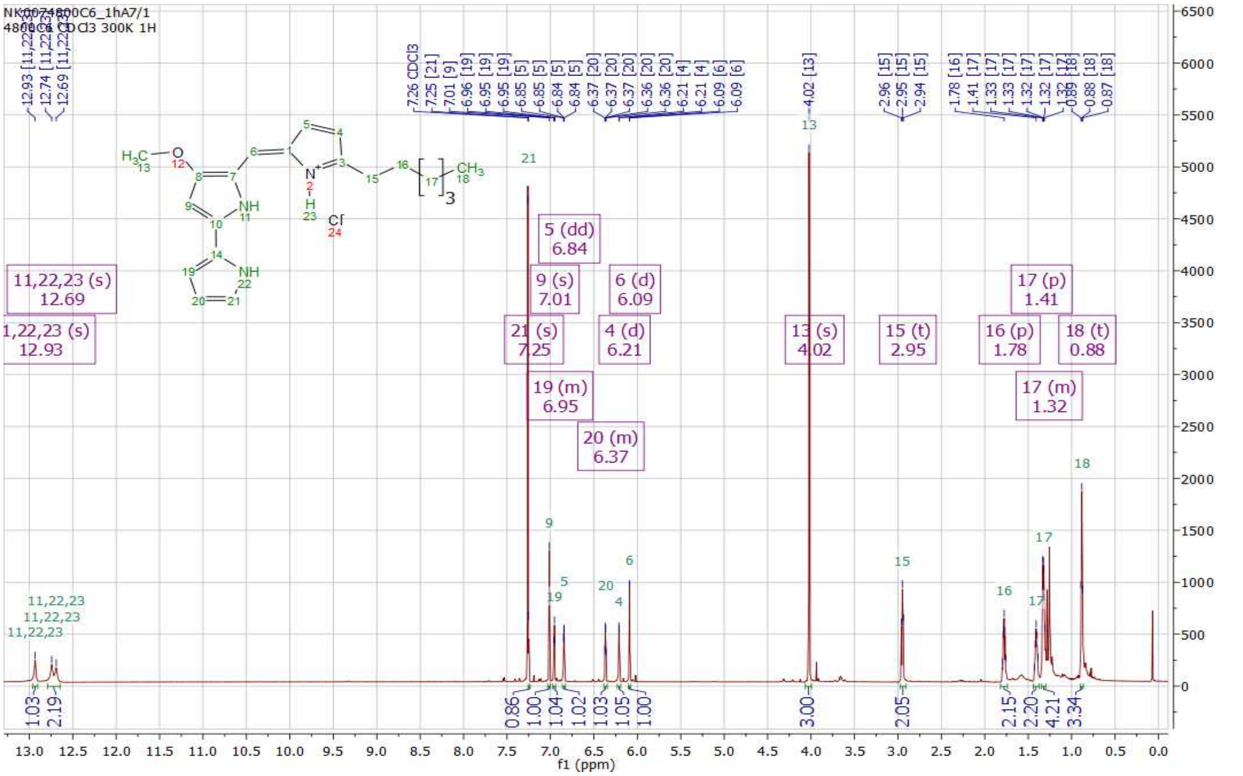

Figure S-3.2.3. ^{1}H NMR spectrum of **C6** in $CDCl_3$.

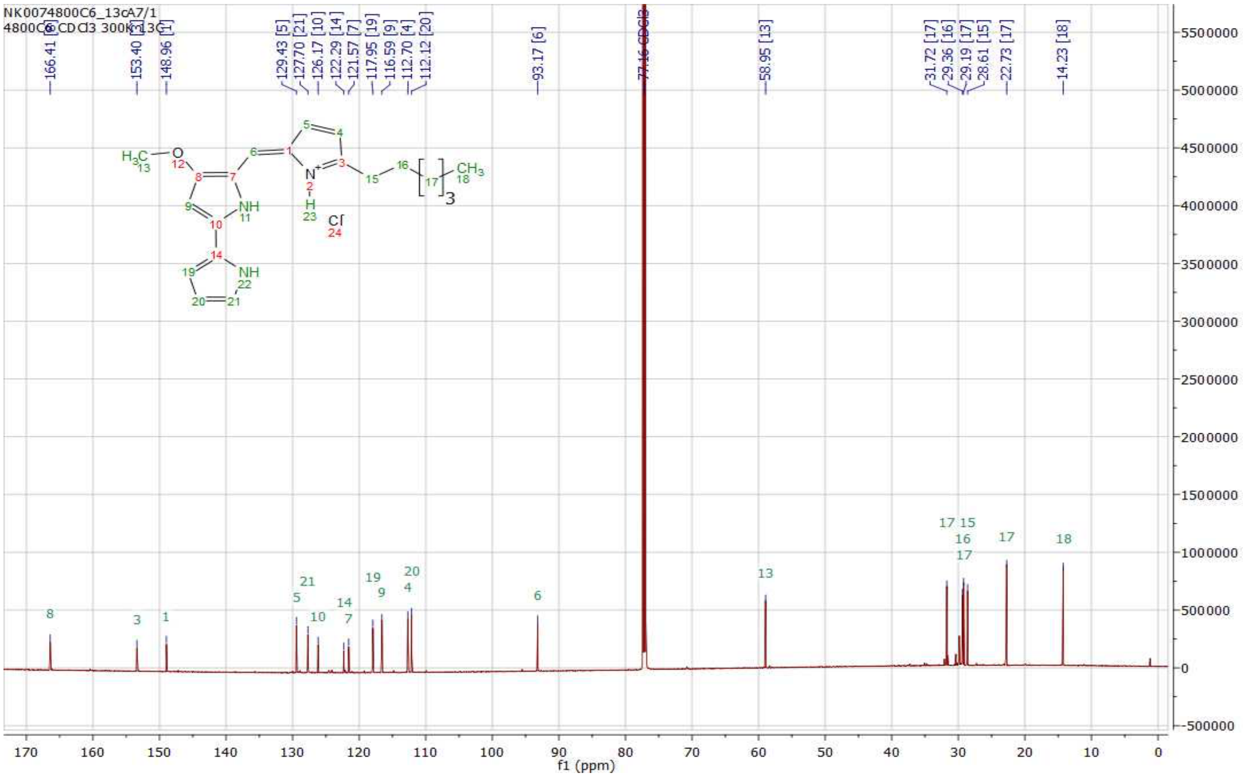

Figure S-3.2.4. ^{13}C NMR spectrum of **C6** in $CDCl_3$.

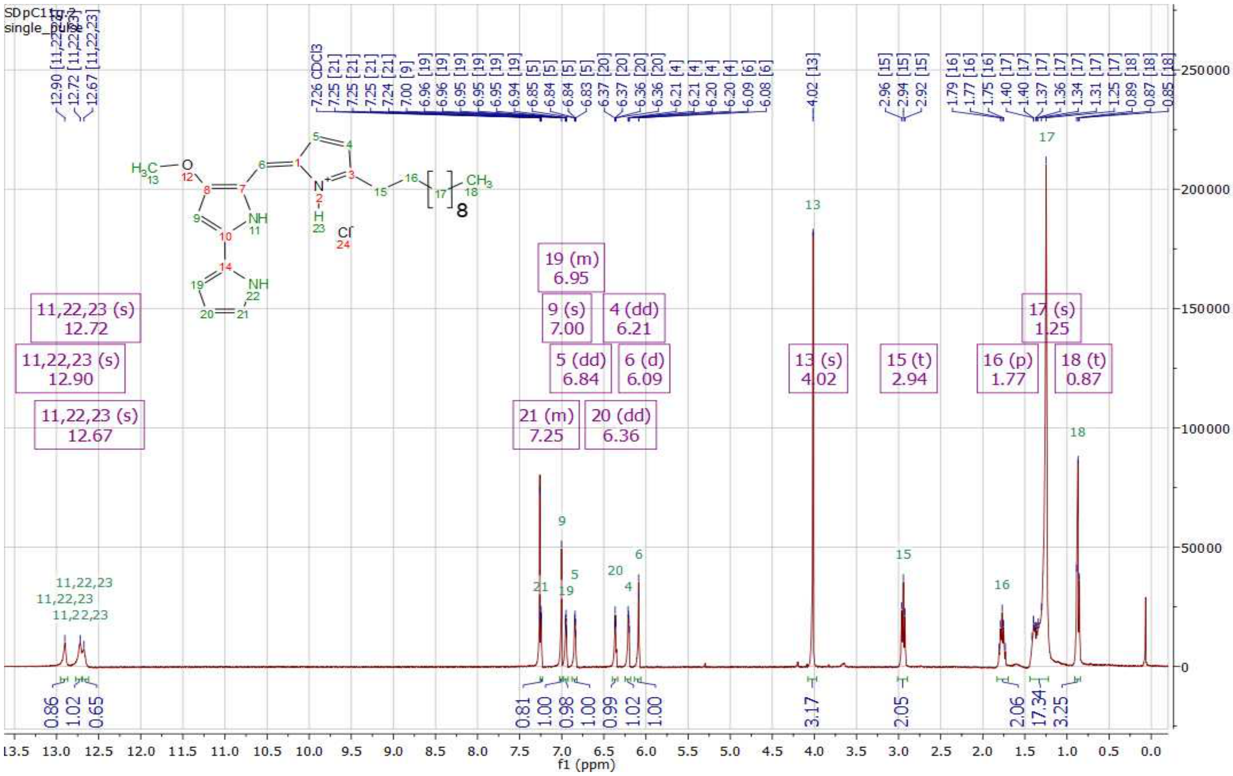

Figure S-3.2.5. ^{1}H NMR spectrum of **C11** in $CDCl_3$.

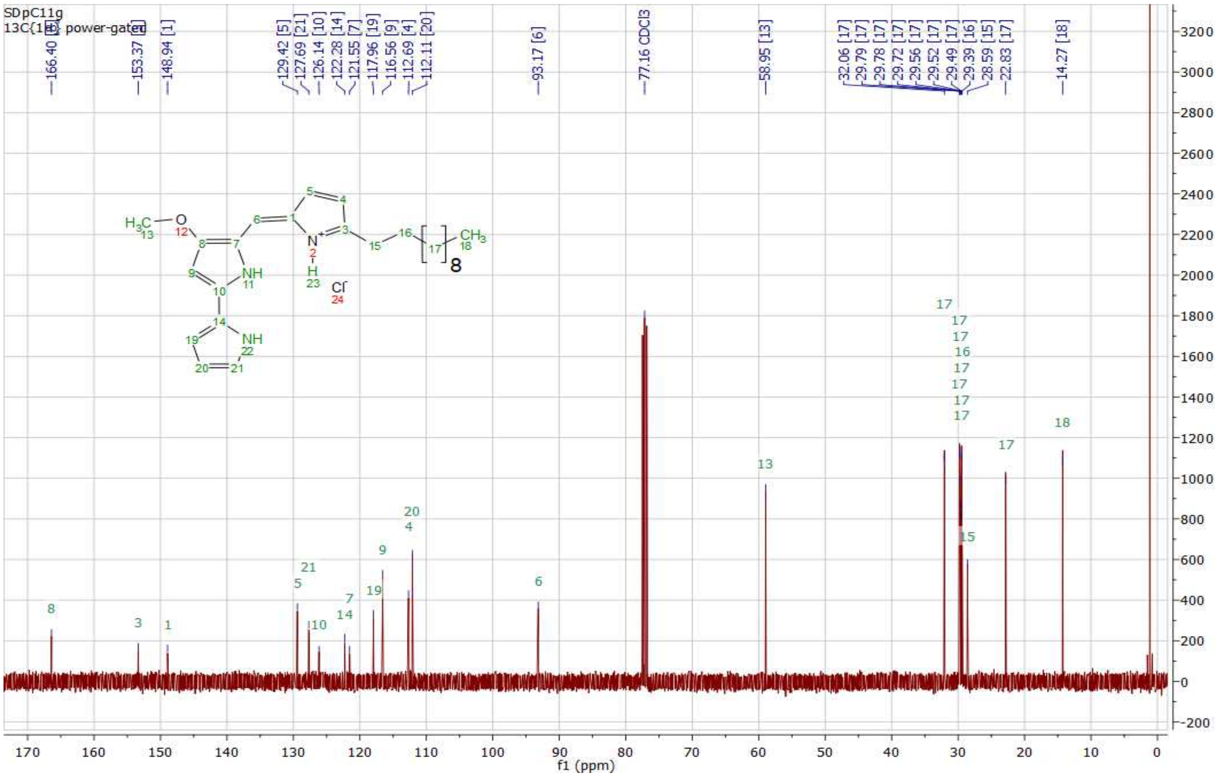

Figure S-3.2.6. ^{13}C NMR spectrum of **C11** in $CDCl_3$.

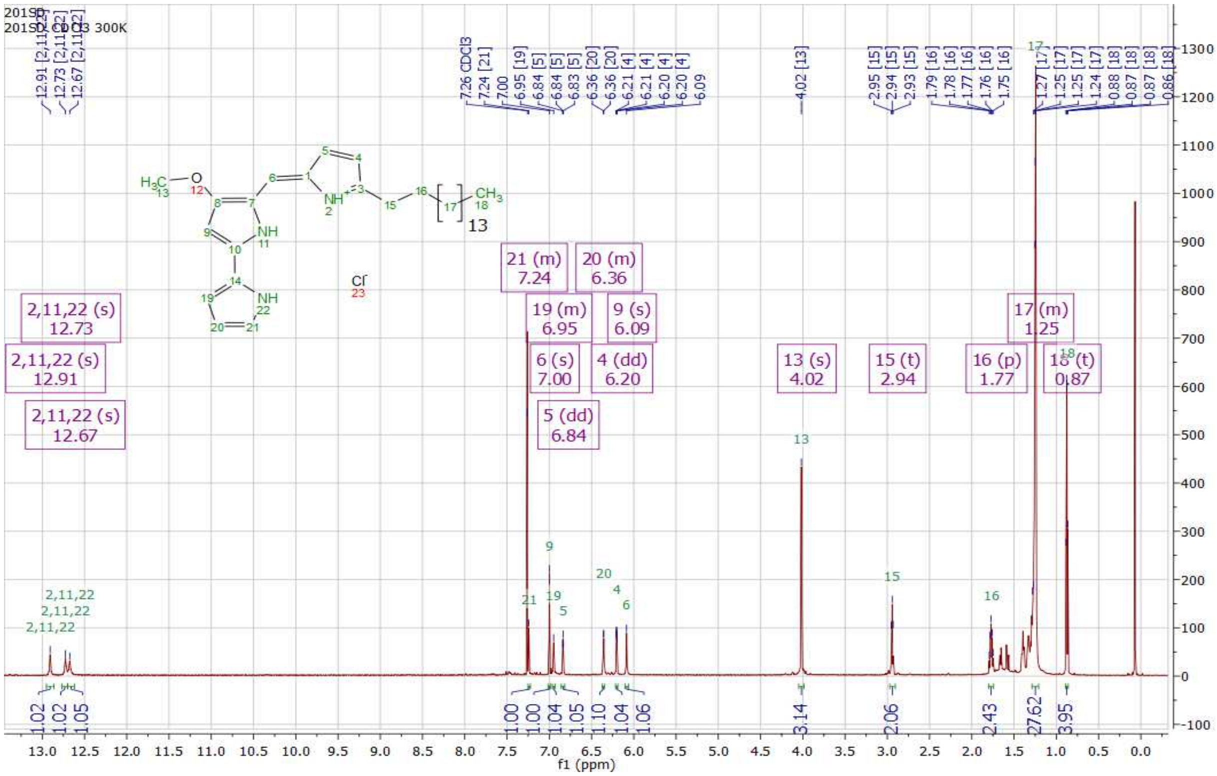

Figure S-3.2.7. ^{1}H NMR spectrum of **C16** in $CDCl_3$.

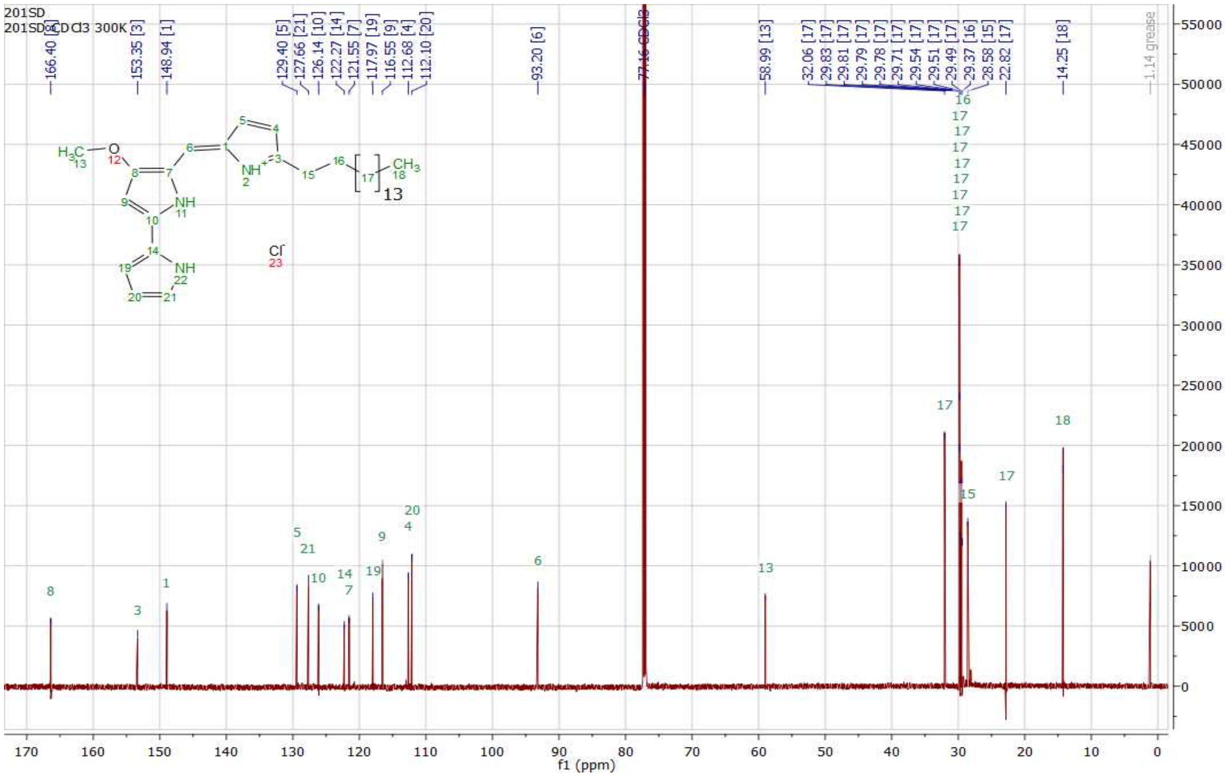

Figure S-3.2.8. ^{13}C NMR spectrum of **C16** in $CDCl_3$.

S-3.3 ESI mass spectra of prodigiosenes

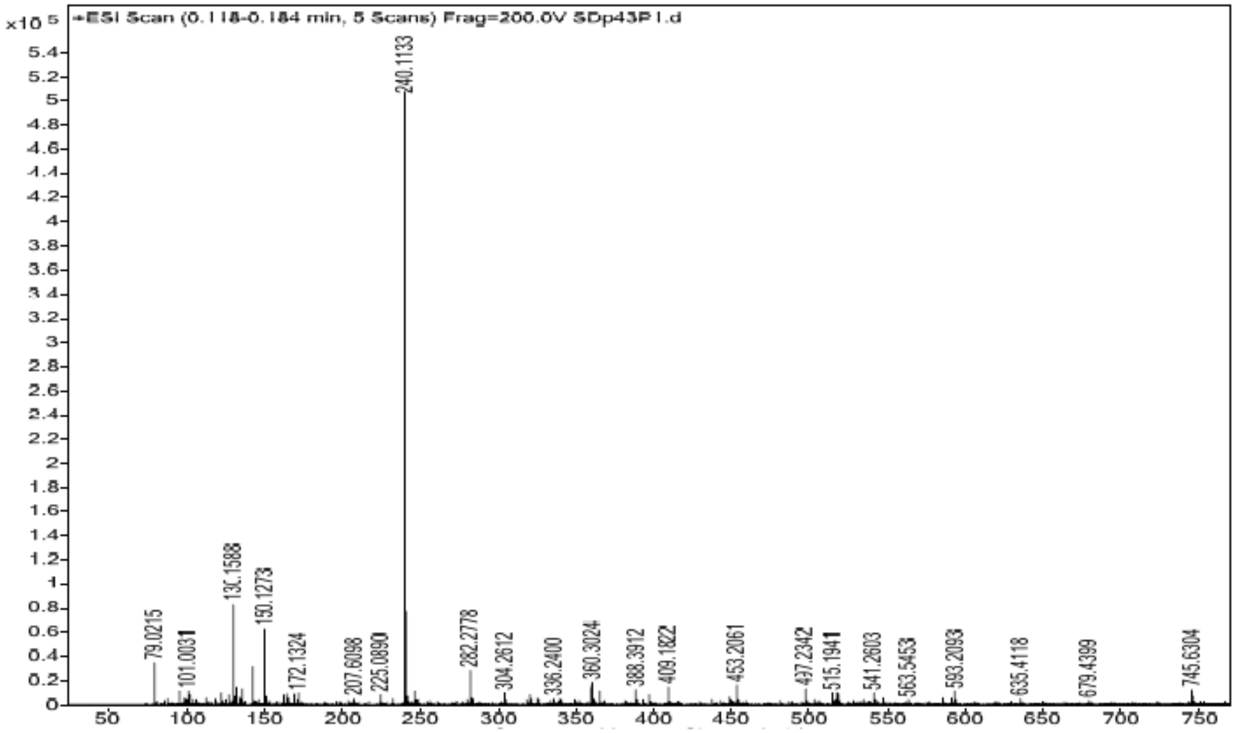

Figure S-3.3.1. ESI mass spectrum of **C0** in methanol.

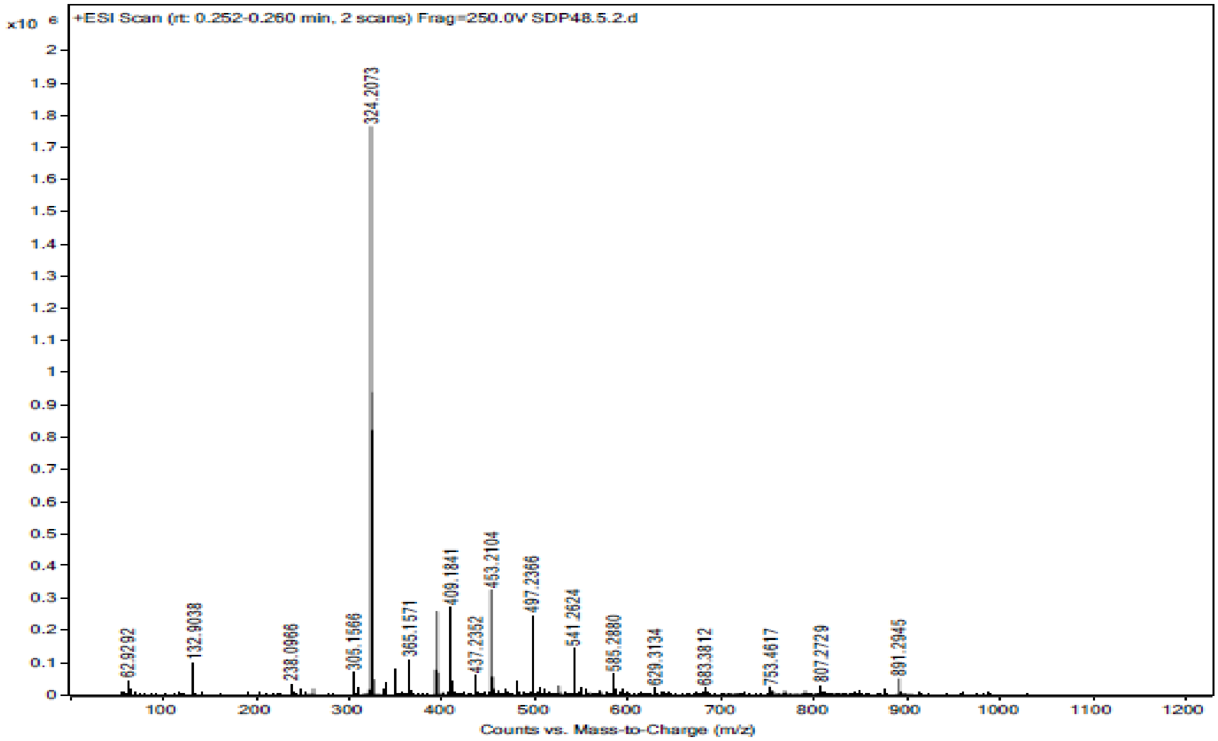

Figure S-3.3.2. ESI mass spectrum of **C6** in methanol.

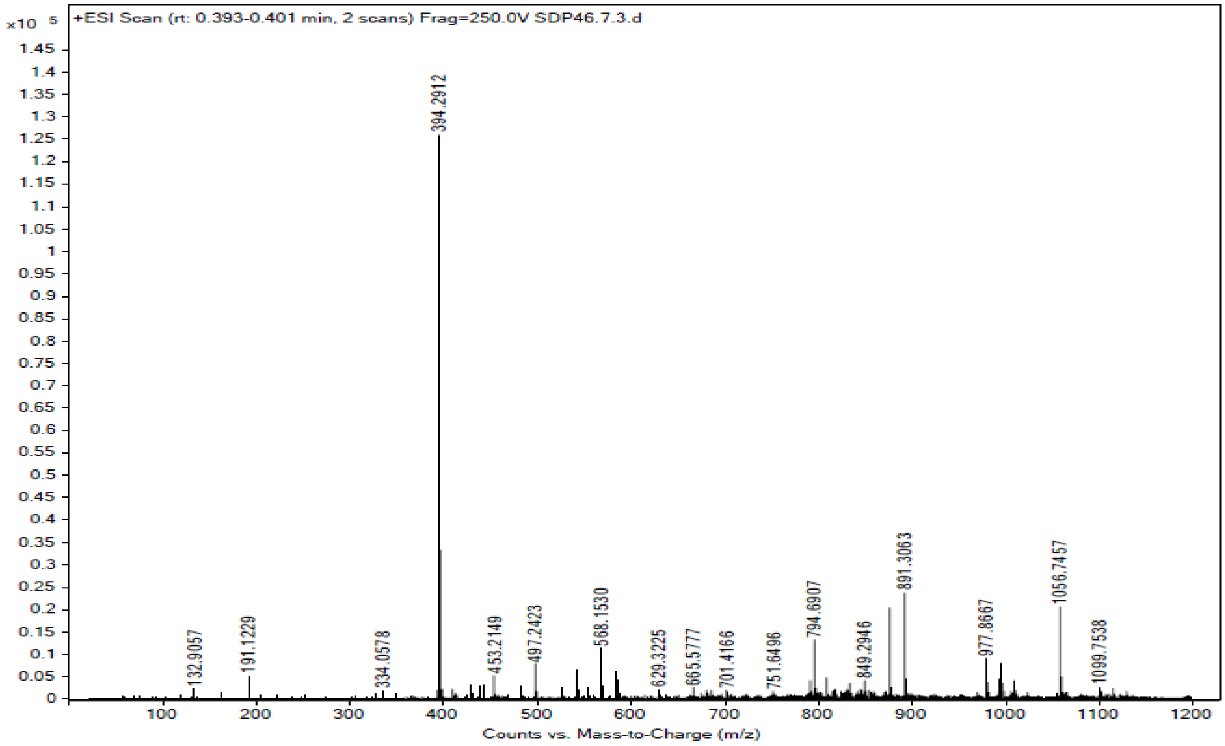

Figure S-3.3.3. ESI mass spectrum of **C11** in methanol.

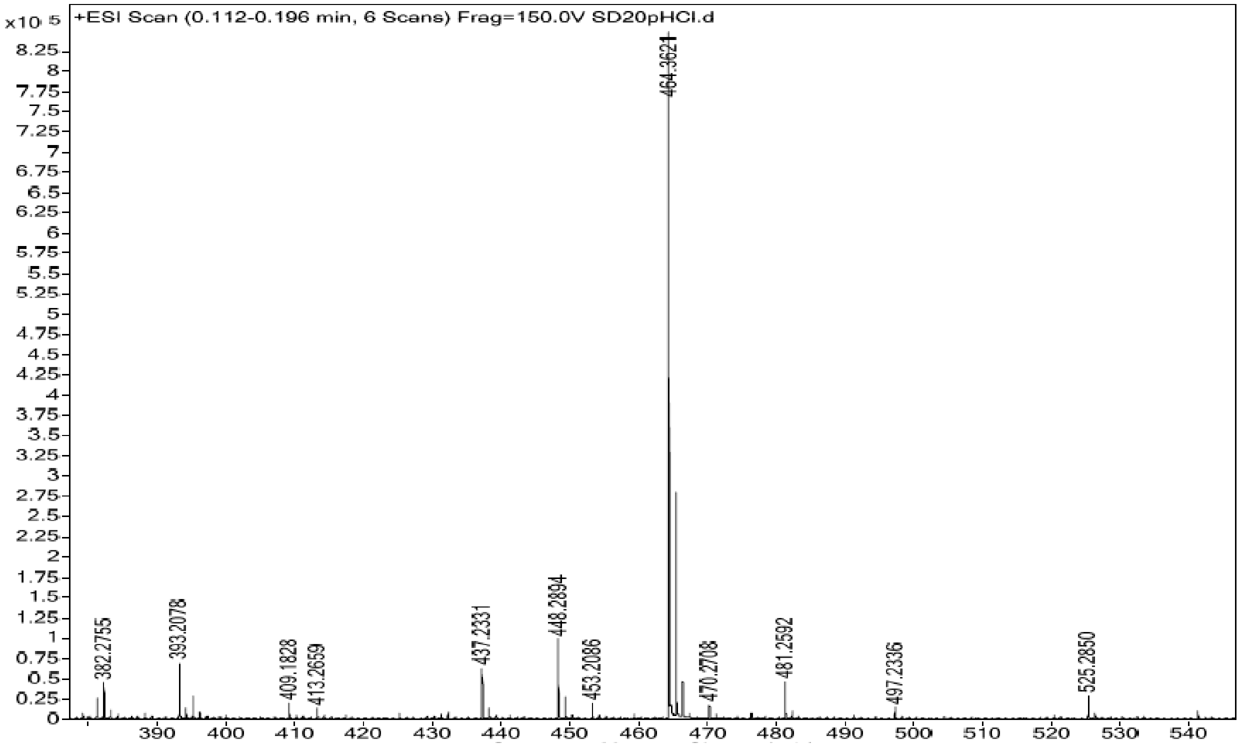

Figure S-3.3.4. ESI mass spectrum of **C16** in methanol.

S-3.4 HPLC data of prodigiosenes

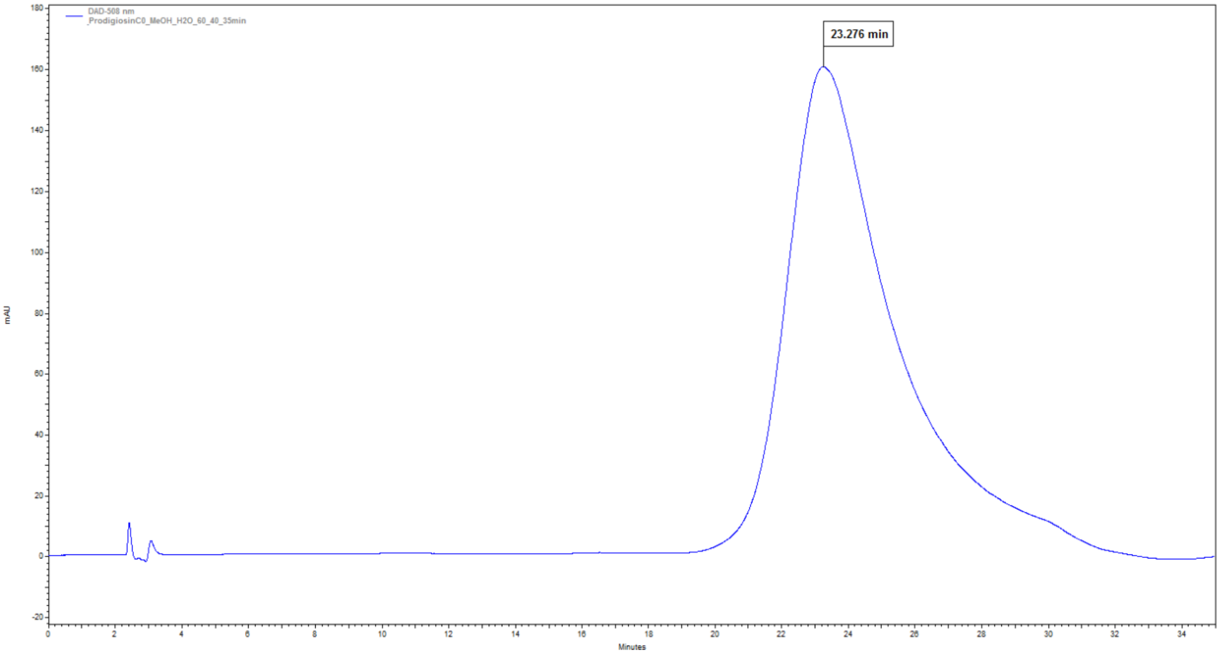

Figure S-3.4.1. Analytic HPLC trace of **C0**. Merck C18 5 µm, 3 x 250 mm. Mobile phase MeOH : Water, 6 : 4 (1 mM HCl), Flow Rate: 0.5 mL/min, λ_{max} = 508 nm.

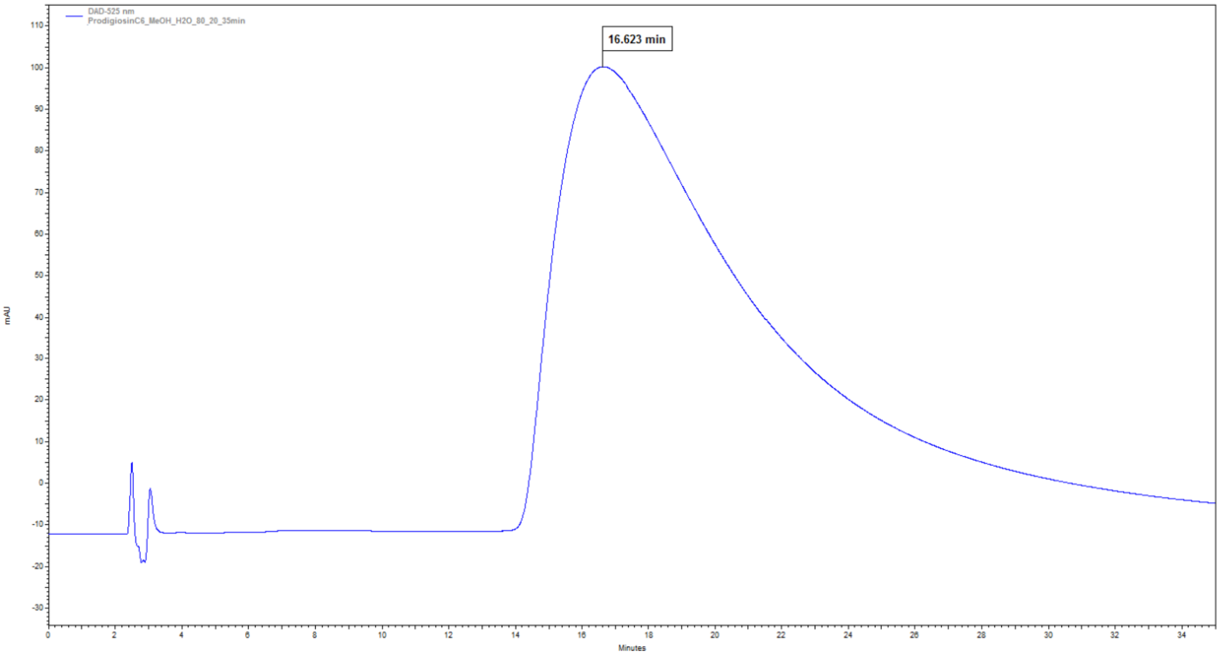

Figure S-3.4.2. Analytic HPLC trace of **C6**. Merck C18 5 µm, 3 x 250 mm. Mobile phase MeOH : Water, 8 : 2 (1 mM HCl), Flow Rate: 0.5 mL/min, λ_{max} = 525 nm.

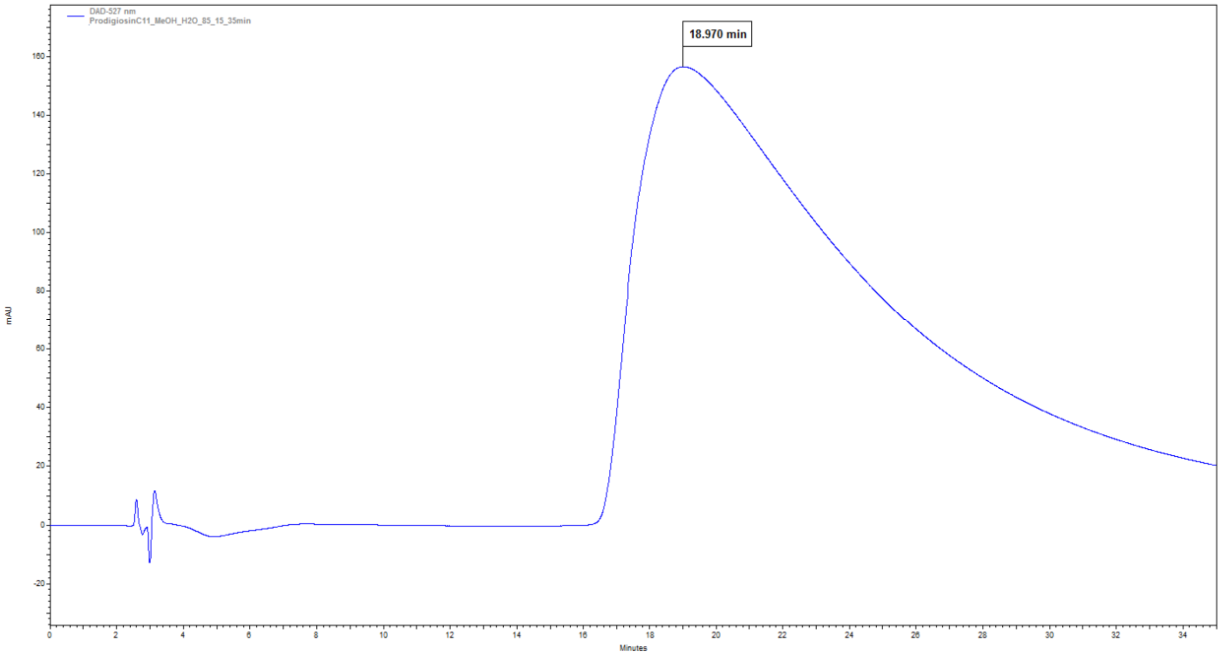

Figure S-3.4.3. Analytic HPLC trace of **C11**. Merck C18 5 µm, 3 x 250 mm. Mobile phase MeOH : Water, 85 : 15 (1 mM HCl), Flow Rate: 0.5 mL/min, λ_{max} = 527 nm.

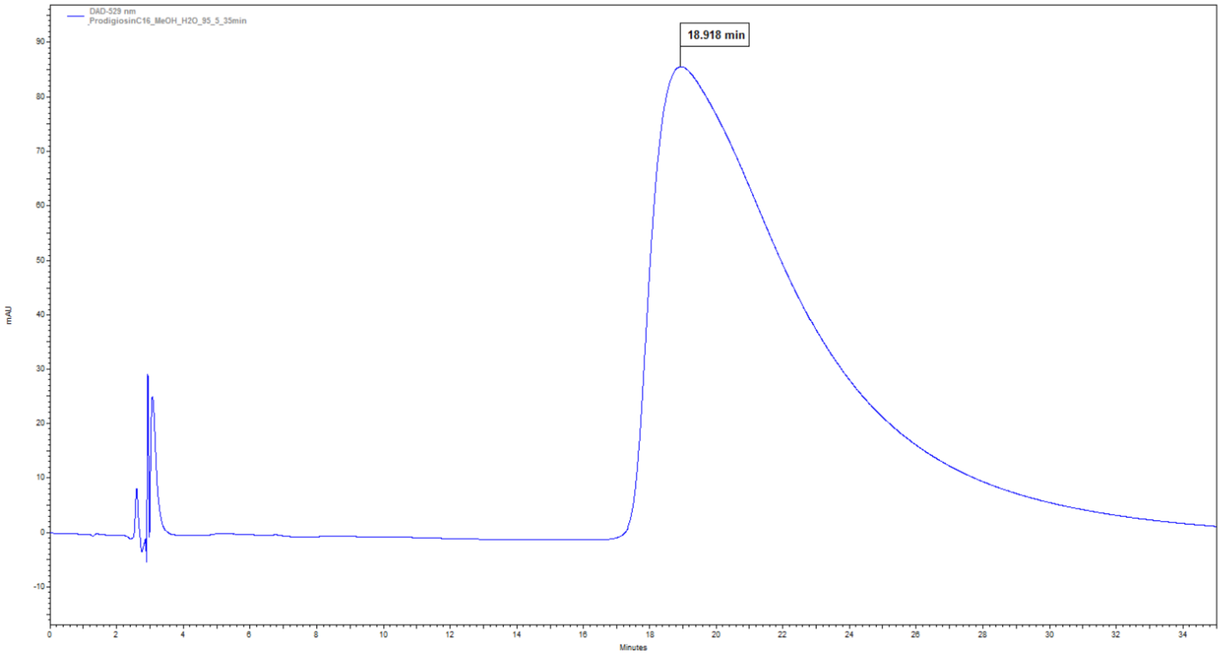

Figure S-3.4.4. Analytic HPLC trace of **C16**. Merck C18 5 µm, 3 x 250 mm. Mobile phase MeOH : Water, 95 : 5 (1 mM HCl), Flow Rate: 0.5 mL/min, λ_{max} = 529 nm.

S-3.5 UV/VIS titration experiments of complexes

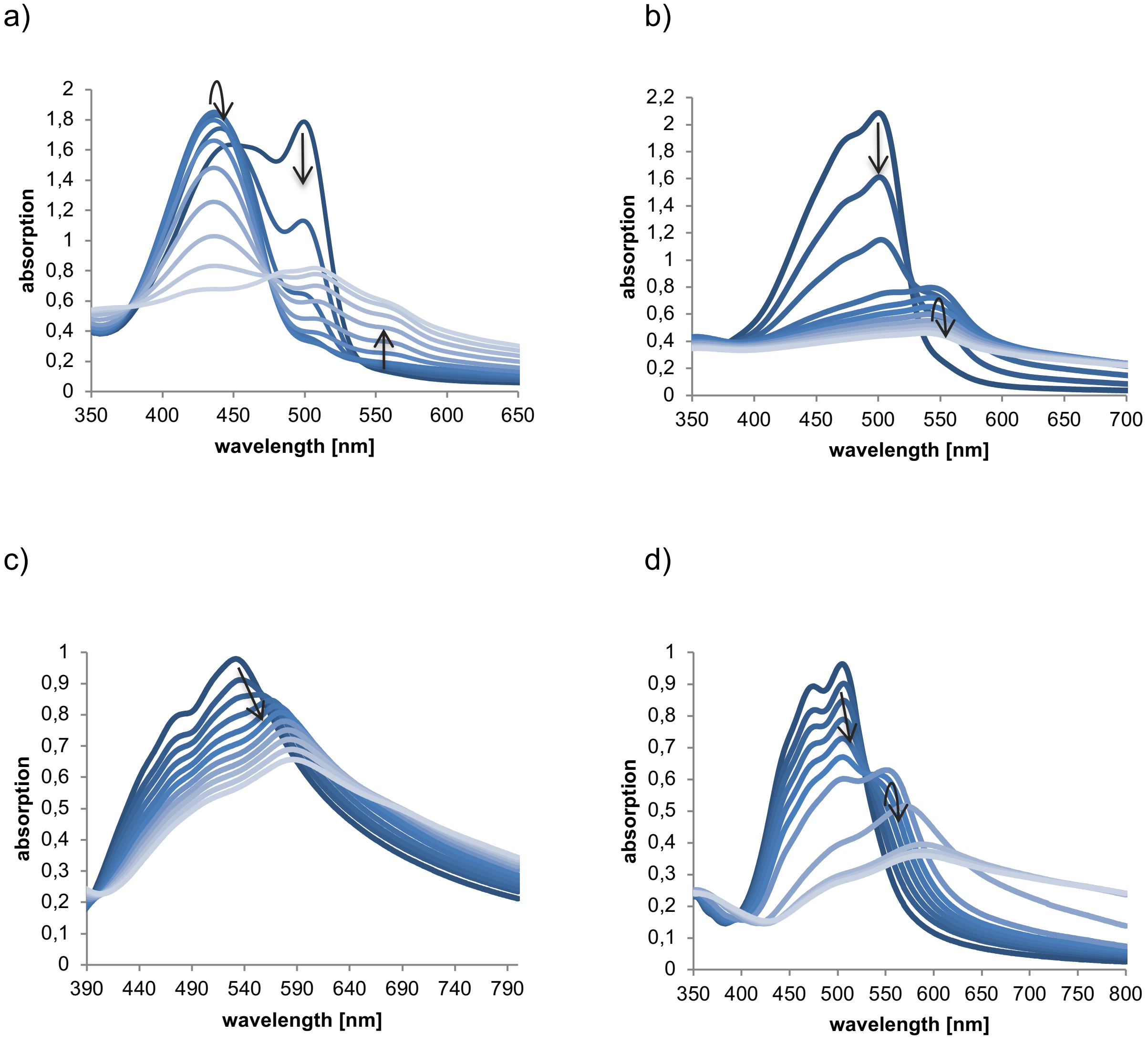

Figure 3.5.1. UV–VIS titration experiments of prodigiosenes with 0.1 eq. aliquots of $Cu(OAc)_2$: a) **C0** in acetonitrile (no buffer), b) **C0** in water at pH = 7.4 (MOPS buffer) c) **C11** in water at pH = 7.4 (MOPS buffer), d) **C16** in water at pH = 7.4 (MOPS buffer).

S-3.6 ESI mass spectra of complexes

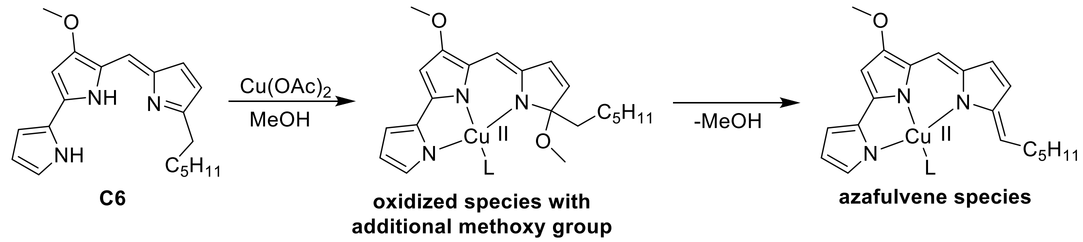

Scheme S-3.6. Formation of oxidized products observed during ESI-MS measurements in methanol, exemplarily for the Cu(**II**) complex of the alkylated prodigiosene **C6**.

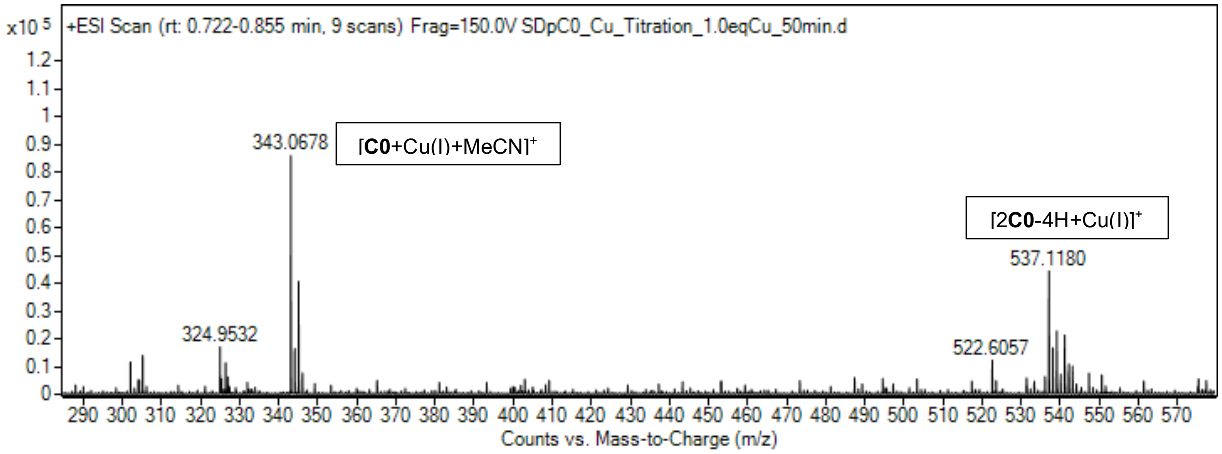

Figure S-3.6.1. ESI mass spectrum of **C0** with 1.0 eq. $Cu(OAc)_2$ measured in acetonitrile.

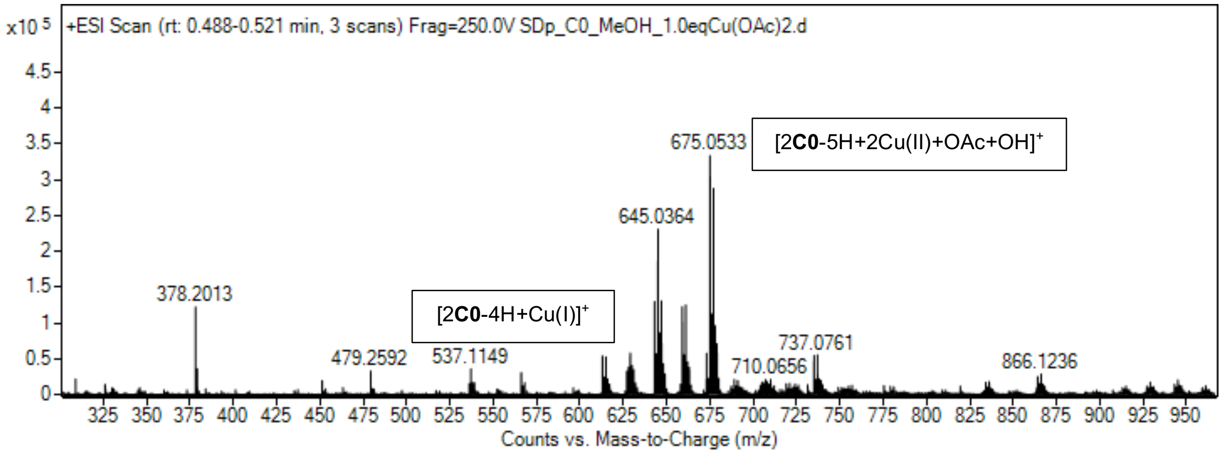

Figure S-3.6.2. ESI mass spectrum of **C0** with 1.0 eq. $Cu(OAc)_2$ measured in methanol.

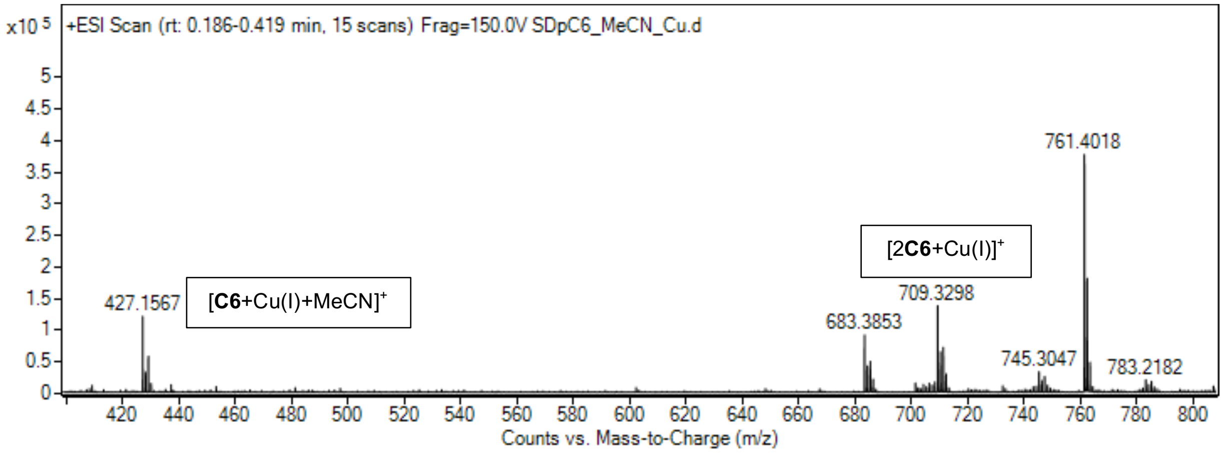

Figure S-3.6.3. ESI mass spectrum of **C6** with 1.0 eq. $Cu(OAc)_2$ measured in acetonitrile.

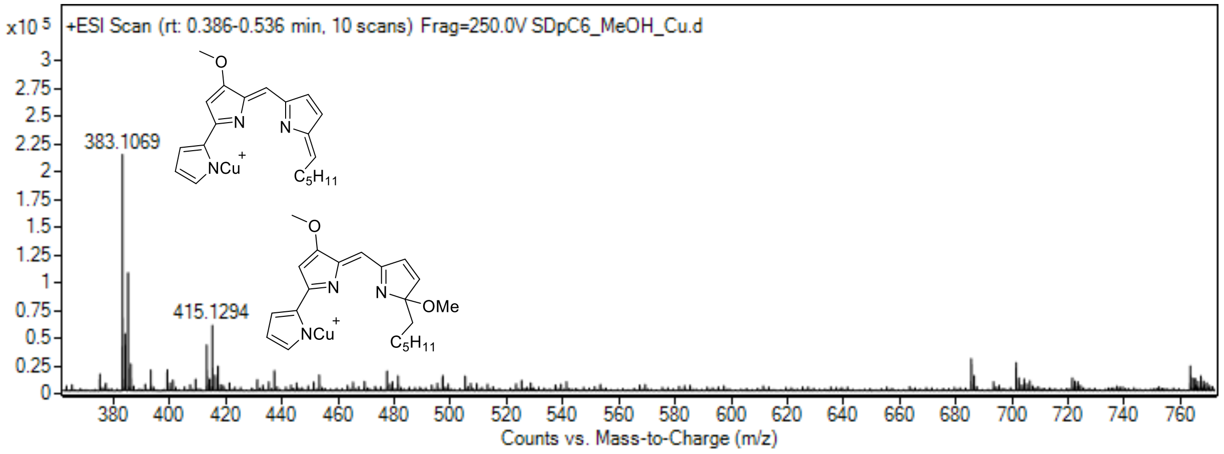

Figure S-3.6.4. ESI mass spectrum of **C6** with 1.0 eq. $Cu(OAc)_2$ measured in methanol.

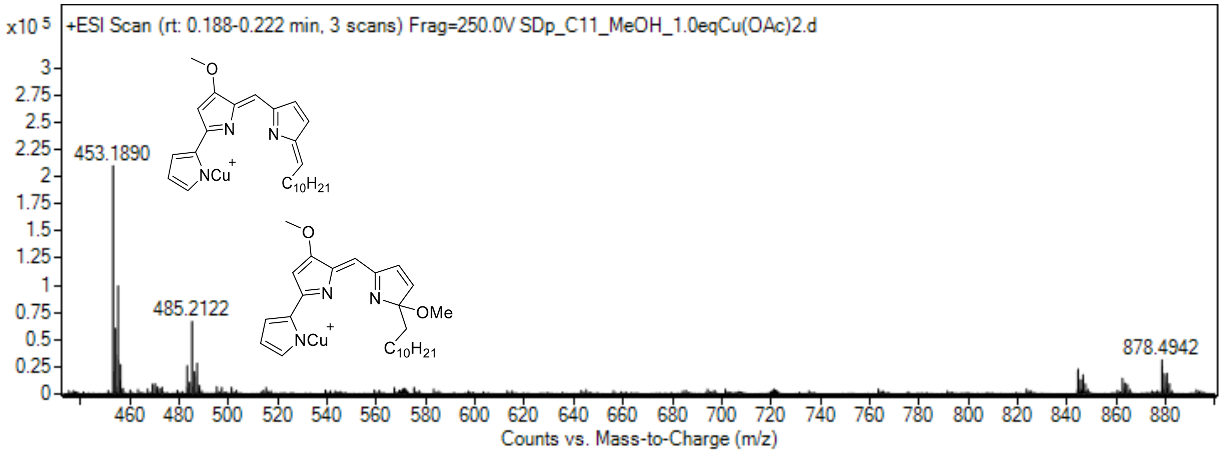

Figure S-3.6.5. ESI mass spectrum of **C11** with 1.0 eq. $Cu(OAc)_2$ measured in methanol.

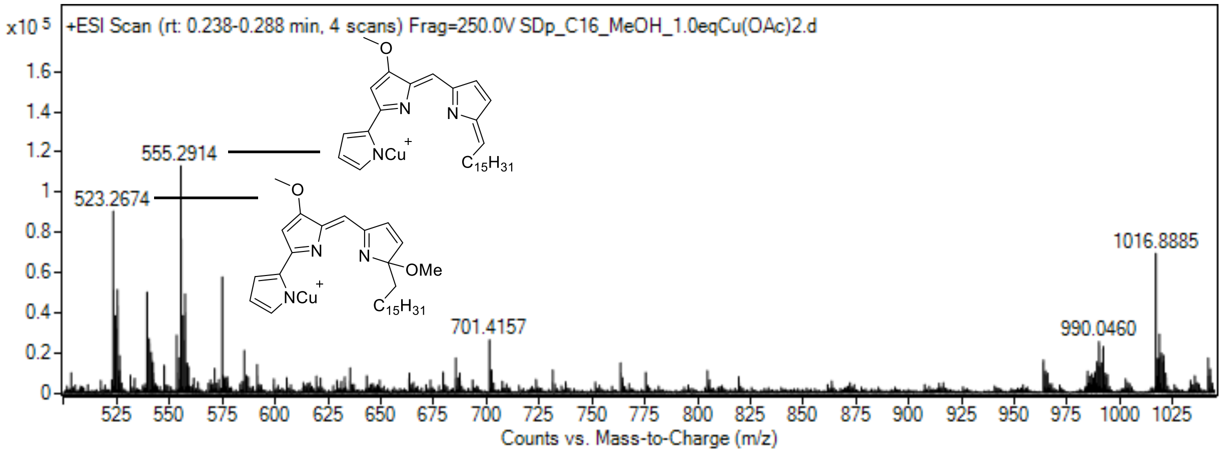

Figure S-3.6.6. ESI mass spectrum of **C16** with 1.0 eq. $Cu(OAc)_2$ measured in methanol.

S-4 Circular Dichroism (CD) spectra

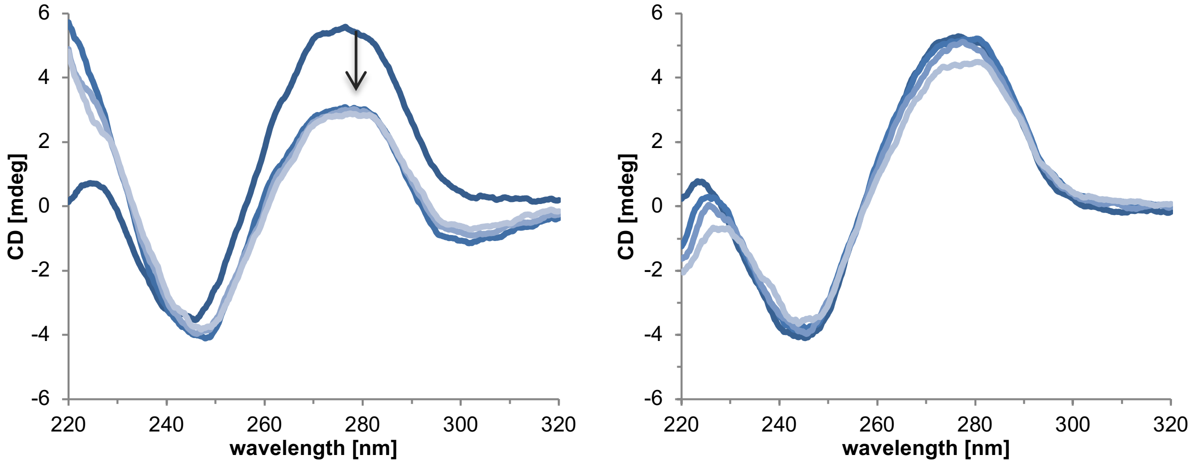

Figure S-4.1. CD spectrum of CT-DNA (100 µM) in MOPS buffer (10 mM, 100 mM NaCl, pH 7.4) with increasing concentrations of **C0** (left: 0, 5, 15, 30 µM) or **C11** (right: 0, 5, 10, 30 µM).

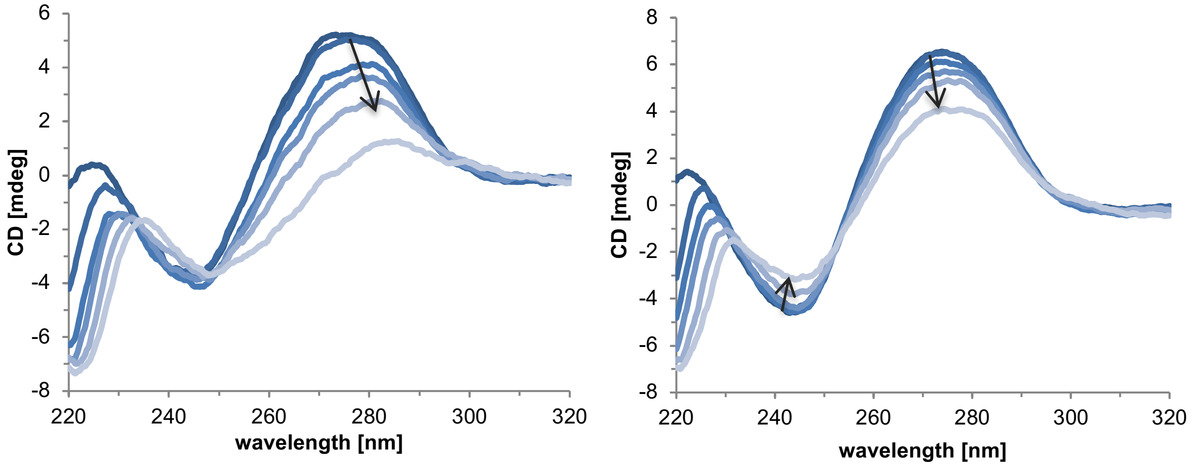

Figure S-4.2. CD spectrum of CT-DNA (100 µM) in MOPS buffer (10 mM, 100 mM NaCl, pH 7.4) with increasing concentrations of *in situ* formed **CuC0** (left: 0, 5, 10, 15, 30, 50 µM) or *in situ* formed **CuC11** (right: 0, 5, 10, 15, 30, 50 µM).

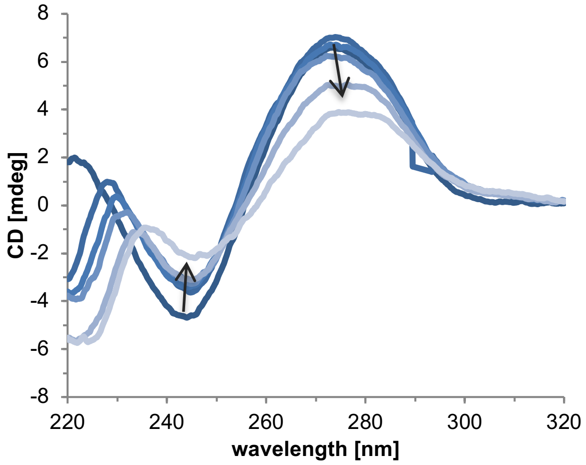

Figure S-4.3. CD spectrum of CT-DNA (100 µM) in MOPS buffer (10 mM, 100 mM NaCl, pH 7.4) with increasing concentrations of *in situ* formed **CuC16** (0, 5, 10, 15, 30, 50 µM).

S-5 DNA melting curves

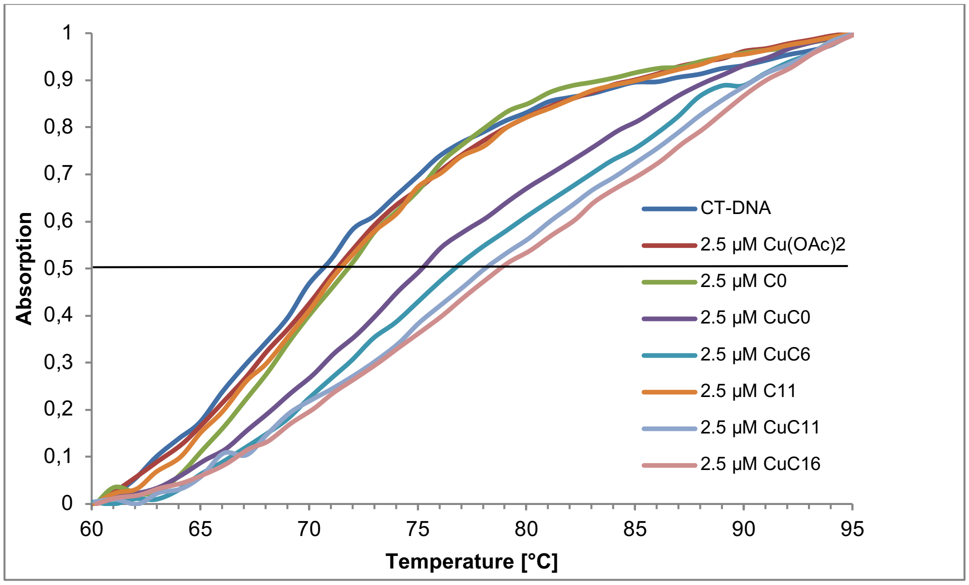

Figure S-5. Normalized melting curves of CT-DNA (50 µM) in MOPS buffer (10 mM, 0.05% DMSO, pH 7.4) and 1% DMSO under addition of ligands **C0**, **C6**, **C11** and **C16** (2.5 µM) in absence and presence of equimolar concentrations of copper(II) acetate.

S-6 Ethidium bromide displacement studies

The ethidium bromide (EB) displacement data were evaluated by using the Stern-Volmer equation (1) where I_0 is the fluorescence emission in absence and I is the fluorescence emission at a defined concentration of the competitive molecule [Q]. The Stern-Volmer constant K_{SV} was hereby determined from the slope of the linear regression of a plot of [Q] against I_0/I. The apparent binding constant K_{app} can then be determined from equation (2), using the binding constant K_{EB} of EB towards DNA (10^7 M^{-1})[16], the used concentration [EB] (1.3 µM), and the concentration of the competitor $[Q]_{50}$, at which 50% of the fluorescence emission of EB has been quenched.

$$\frac{I_0}{I} = 1 + K_{SV}[\mathrm{Q}] \qquad (1)$$

$$K_{EB}[\mathrm{EB}] = K_{app}[\mathrm{Q}]_{50} \qquad (2)$$

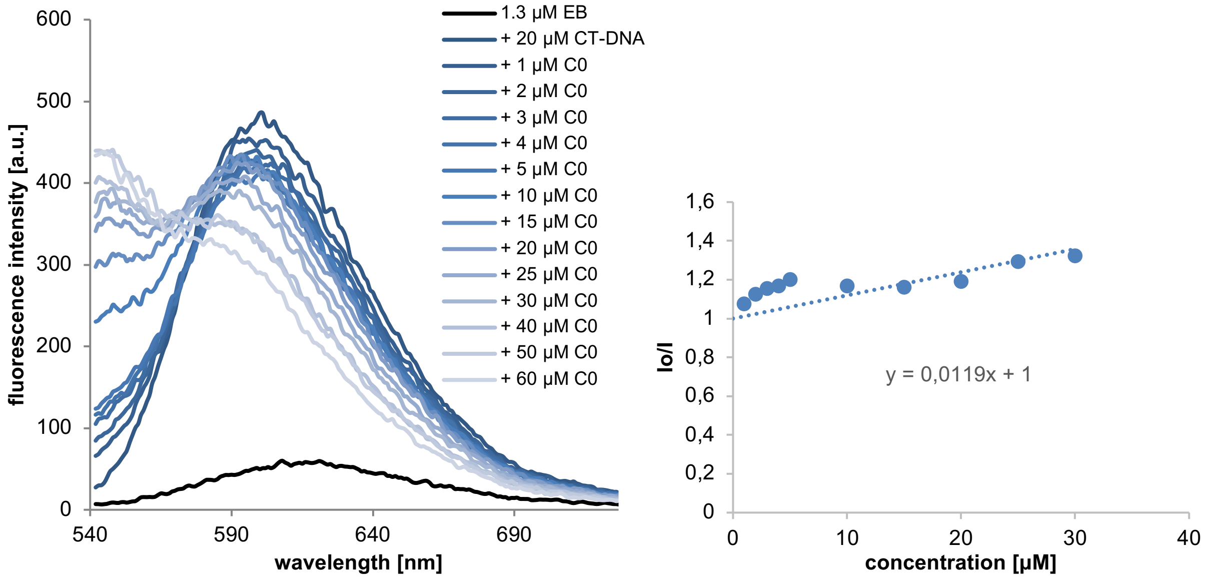

Figure S-6.1. (left) EB displacement studies (1.3 µM EB, 20 µM CT-DNA) in MOPS buffer (10 mM, 100 mM NaCl, pH 7.4) with increasing concentrations of **C0**. (right) Stern-Volmer plot used to determine the Stern-Volmer constant K_{sv} and subsequently the apparent binding constant K_{app} for **C0**. λ_{ex} = 520 nm.

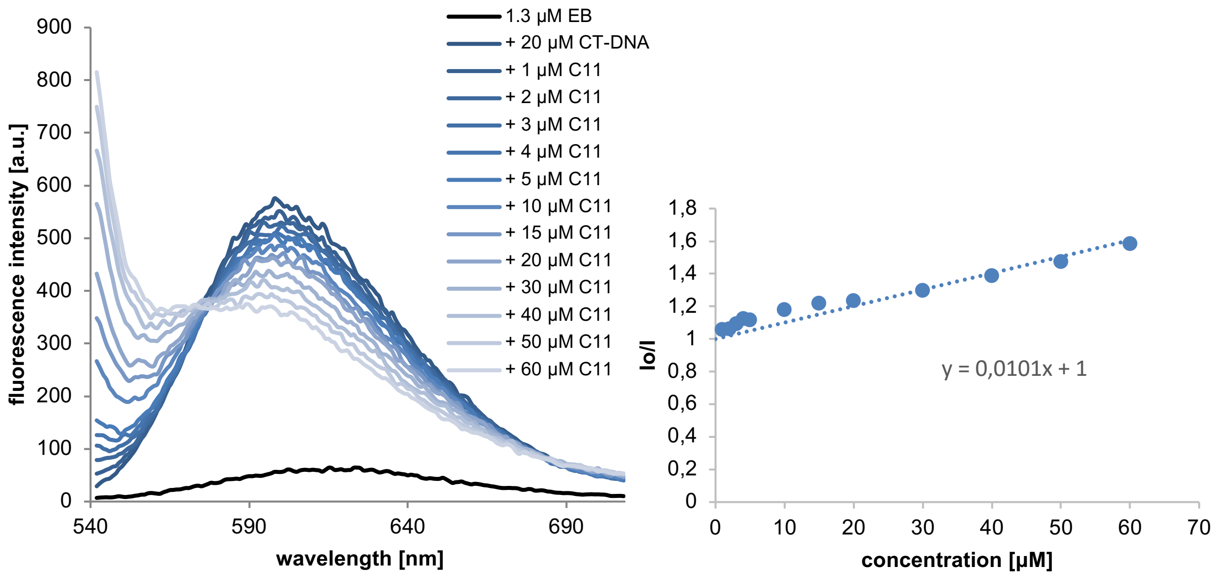

Figure S-6.2. (left) EB displacement studies (1.3 µM EB, 20 µM CT-DNA) in MOPS buffer (10 mM, 100 mM NaCl, pH 7.4) with increasing concentrations of **C11**. (right) Stern-Volmer plot used to determine the Stern-Volmer constant K_{sv} and subsequently the apparent binding constant K_{app} for **C11**. λ_{ex} = 520 nm.

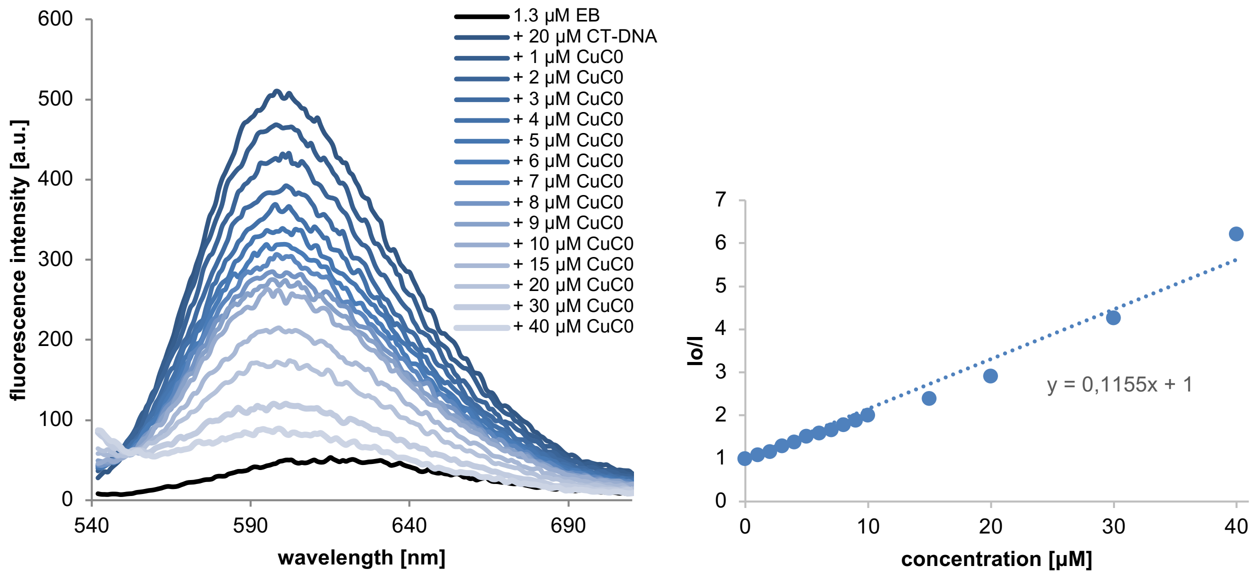

Figure S-6.3. (left) EB displacement studies (1.3 µM EB, 20 µM CT-DNA) in MOPS buffer (10 mM, 100 mM NaCl, pH 7.4) with increasing concentrations of **CuC0**. (right) Stern-Volmer plot used to determine the Stern-Volmer constant K_{sv} and subsequently the apparent binding constant K_{app} for **CuC0**. λ_{ex} = 520 nm.

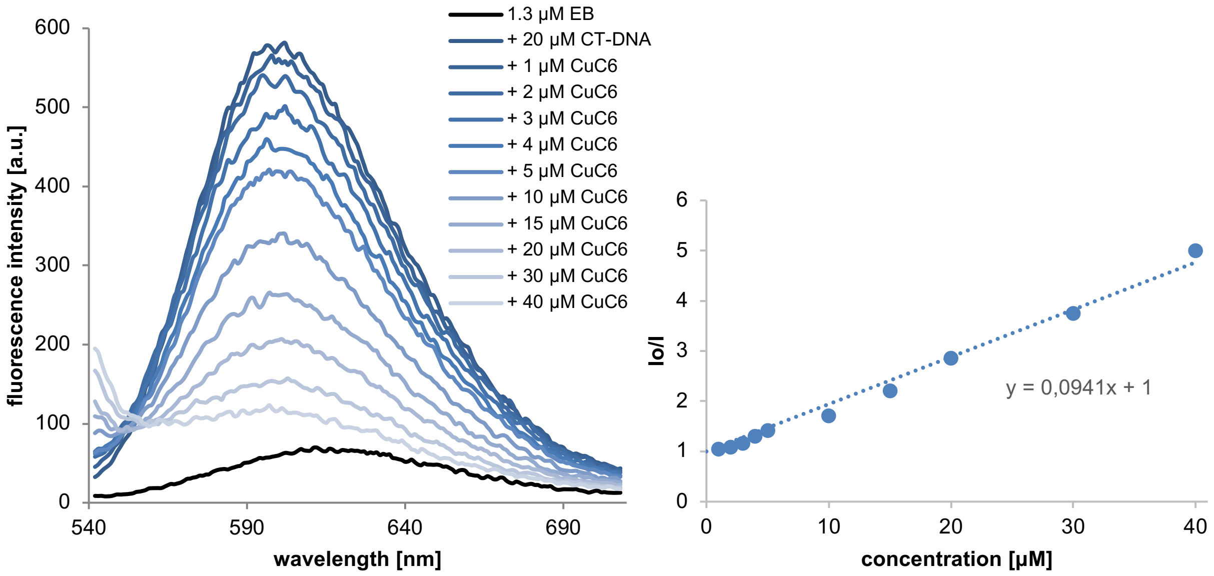

Figure S-6.4. (left) EB displacement studies (1.3 µM EB, 20 µM CT-DNA) in MOPS buffer (10 mM, 100 mM NaCl, pH 7.4) with increasing concentrations of **CuC6**. (right) Stern-Volmer plot used to determine the Stern-Volmer constant K_{sv} and subsequently the apparent binding constant K_{app} for **CuC6**. λ_{ex} = 520 nm.

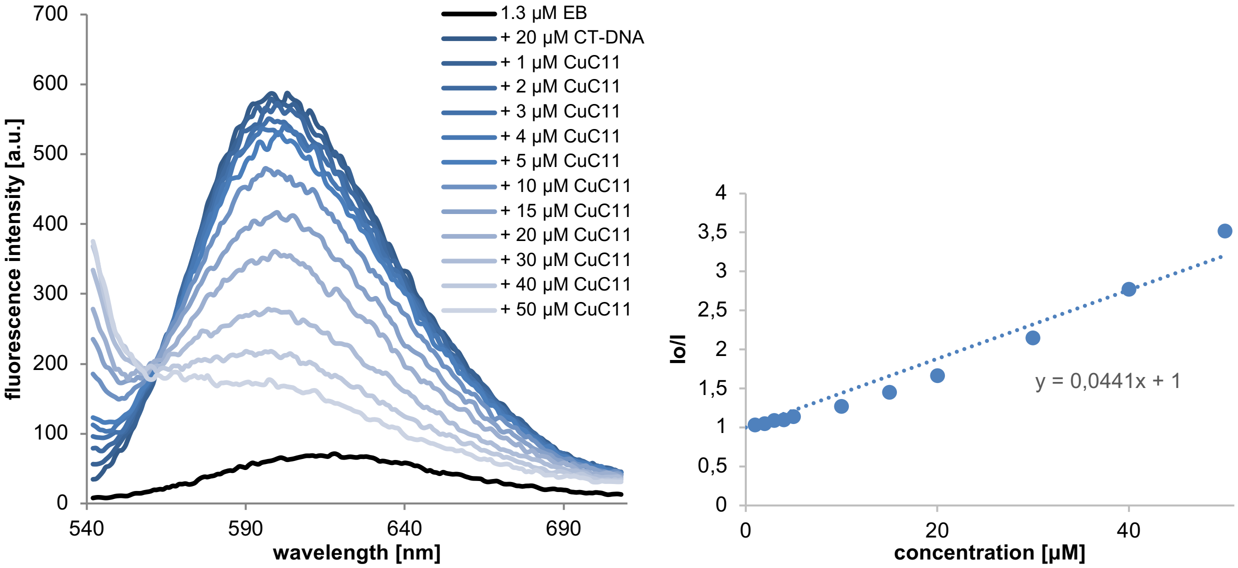

Figure S-6.5. (left) EB displacement studies (1.3 µM EB, 20 µM CT-DNA) in MOPS buffer (10 mM, 100 mM NaCl, pH 7.4) with increasing concentrations of **CuC11**. (right) Stern-Volmer plot used to determine the Stern-Volmer constant K_{sv} and subsequently the apparent binding constant K_{app} for **CuC11**. λ_{ex} = 520 nm.

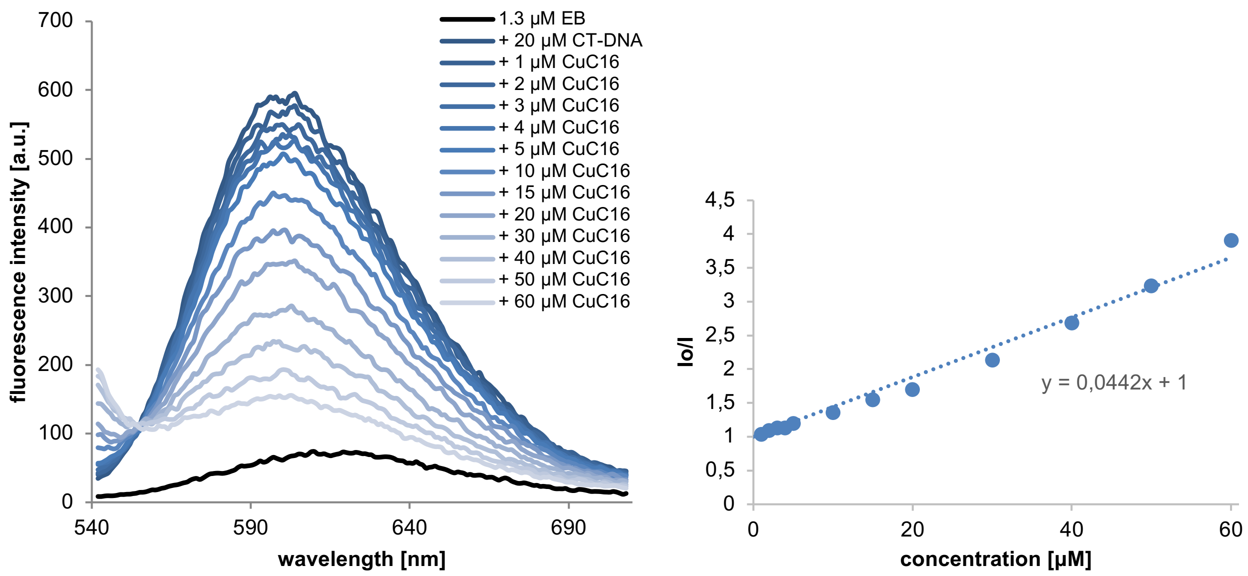

Figure S-6.6. (left) EB displacement studies (1.3 µM EB, 20 µM CT-DNA) in MOPS buffer (10 mM, 100 mM NaCl, pH 7.4) with increasing concentrations of **CuC16**. (right) Stern-Volmer plot used to determine the Stern-Volmer constant K_{sv} and subsequently the apparent binding constant K_{app} for **CuC16**. λ_{ex} = 520 nm.

S-7 Fluorescence titration experiments

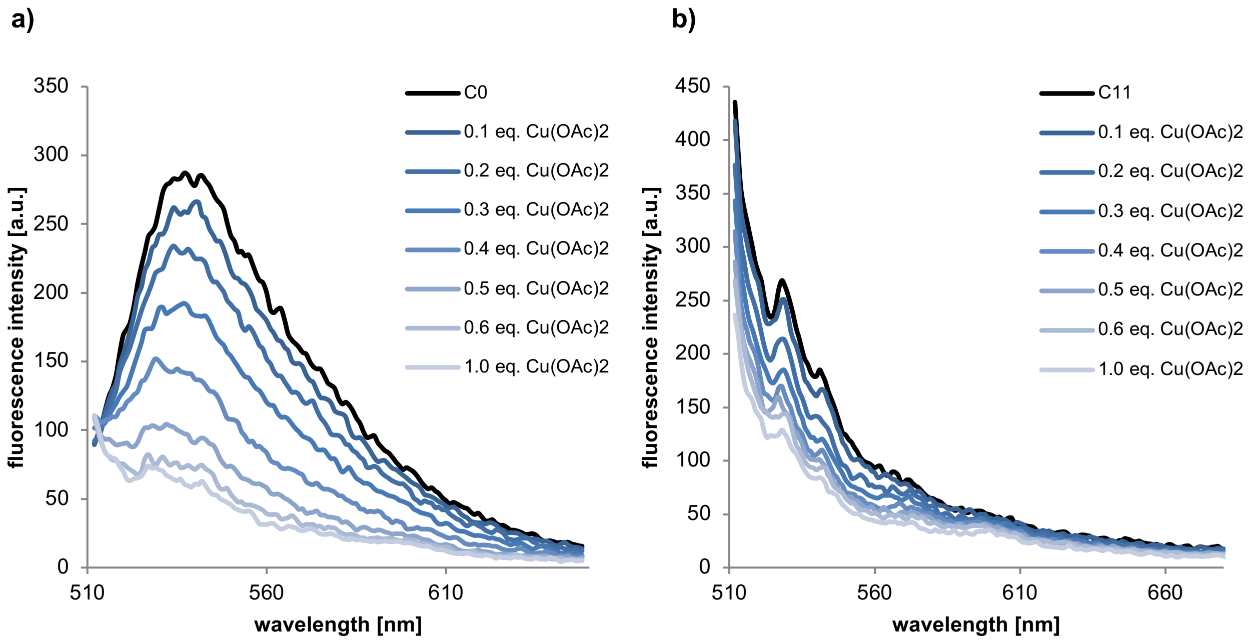

Figure S-7. Fluorescence titration experiments of **C0** (a) and **C11** (b) (40 µM) in MOPS buffer (10 mM, 100 mM NaCl,10% DMSO, pH 7.4) with increasing concentrations of $Cu(OAc)_2$. λ_{ex} = 500 nm.

S-8 DNA cleavage

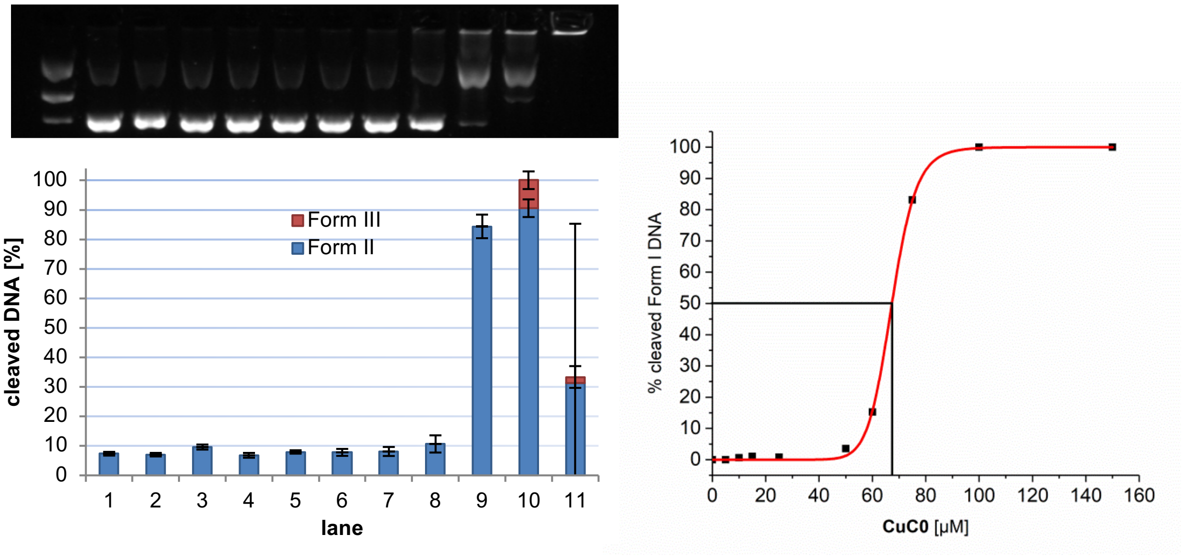

Figure S-8.1. (Left) Agarose gel and visualization of the cleavage of plasmid DNA pBR322 (0.2 µg) with increasing concentrations of *in situ* formed complex **CuC0** in MOPS buffer (10 mM, 100 mM NaCl, pH 7.4) after incubation for 1 h at 37 °C. Lane 0: DNA ladder, lane 1: DNA reference, lane 2: **C0** (150 µM), lane 3: $Cu(OAc)_2$ (150 µM), lane 4: **CuC0** (5 µM), lane 5: **CuC0** (10 µM), lane 6: **CuC0** (15 µM), lane 7: **CuC0** (25 µM), lane 8: **CuC0** (50 µM), lane 9: **CuC0** (75 µM), lane 10: **CuC0** (100 µM), lane 11: **CuC0** (150 µM). (right) Graphical visualization of the calculated EC_{50} value for **CuC0**.

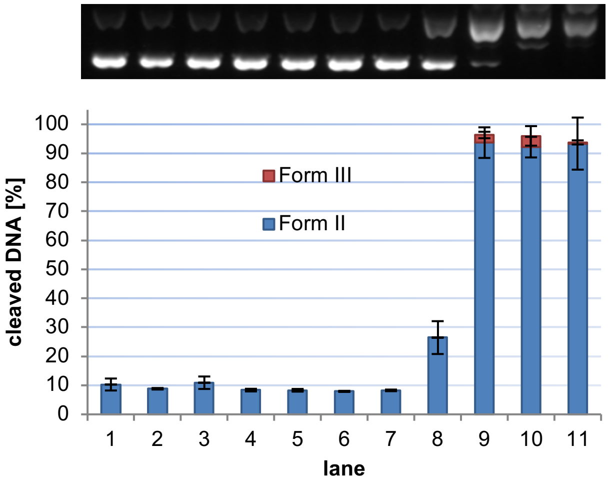

Figure S-8.2. Agarose gel and visualization of the cleavage of plasmid DNA pBR322 (0.2 µg) with increasing concentrations of *in situ* formed complex **CuC6** in MOPS buffer (10 mM, 100 mM NaCl, pH 7.4) after incubation for 1 h at 37 °C. Lane 1: DNA reference, lane 2: **C6** (150 µM), lane 3: $Cu(OAc)_2$ (150 µM), lane 4: **CuC6** (5 µM), lane 5: **CuC6** (10 µM), lane 6: **CuC6** (15 µM), lane 7: **CuC6** (25 µM), lane 8: **CuC6** (50 µM), lane 9: **CuC6** (75 µM), lane 10: **CuC6** (100 µM), lane 11: **CuC6** (150 µM).

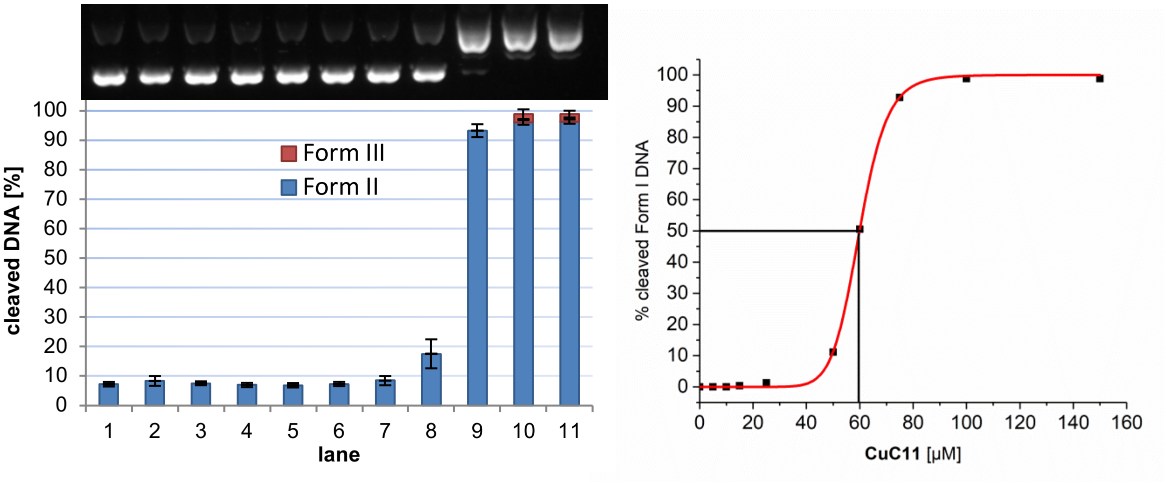

Figure S-8.3. (Left) Agarose gel and visualization of the cleavage of plasmid DNA pBR322 (0.2 µg) with increasing concentrations of *in situ* formed complex **CuC11** in MOPS buffer (10 mM, 100 mM NaCl, pH 7.4) after incubation for 1 h at 37 °C. Lane 1: DNA reference, lane 2: **C11** (150 µM), lane 3: $Cu(OAc)_2$ (150 µM), lane 4: **CuC11** (5 µM), lane 5: **CuC11** (10 µM), lane 6: **CuC11** (15 µM), lane 7: **CuC11** (25 µM), lane 8: **CuC11** (50 µM), lane 9: **CuC11** (75 µM), lane 10: **CuC11** (100 µM), lane 11: **CuC11** (150 µM). (right) Graphical visualization of the calculated EC_{50} value for **CuC11**.

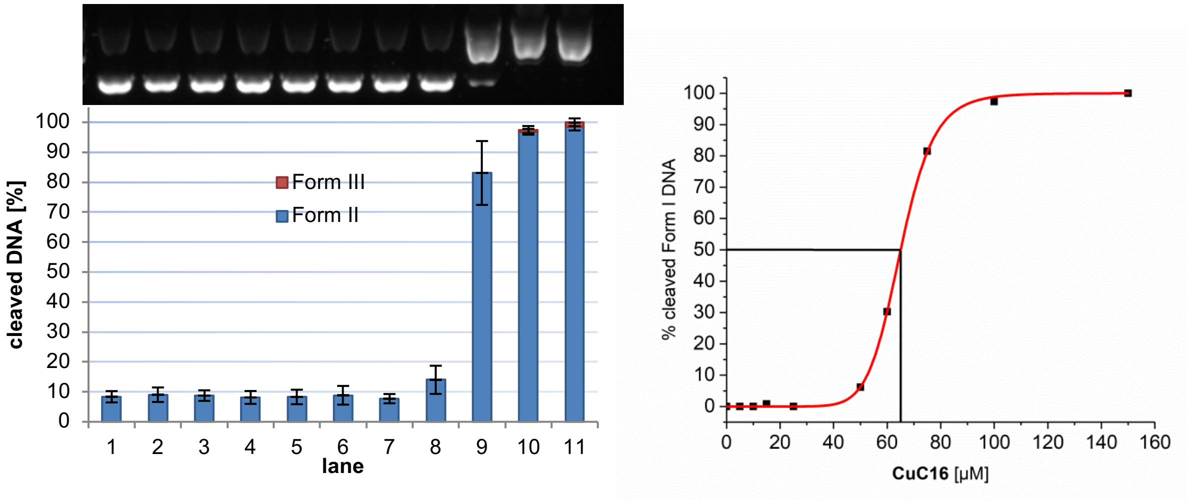

Figure S-8.4. (Left) Agarose gel and visualization of the cleavage of plasmid DNA pBR322 (0.2 µg) with increasing concentrations of *in situ* formed complex **CuC6** in MOPS buffer (10 mM, 100 mM NaCl, pH 7.4) after incubation for 1 h at 37 °C. Lane 1: DNA reference, lane 2: **C16** (150 µM), lane 3: $Cu(OAc)_2$ (150 µM), lane 4: **CuC16** (5 µM), lane 5: **CuC16** (10 µM), lane 6: **CuC16** (15 µM), lane 7: **CuC16** (25 µM), lane 8: **CuC16** (50 µM), lane 9: **CuC16** (75 µM), lane 10: **CuC16** (100 µM), lane 11: **CuC16** (150 µM). (right) Graphical visualization of the calculated EC_{50} value for **CuC16**.

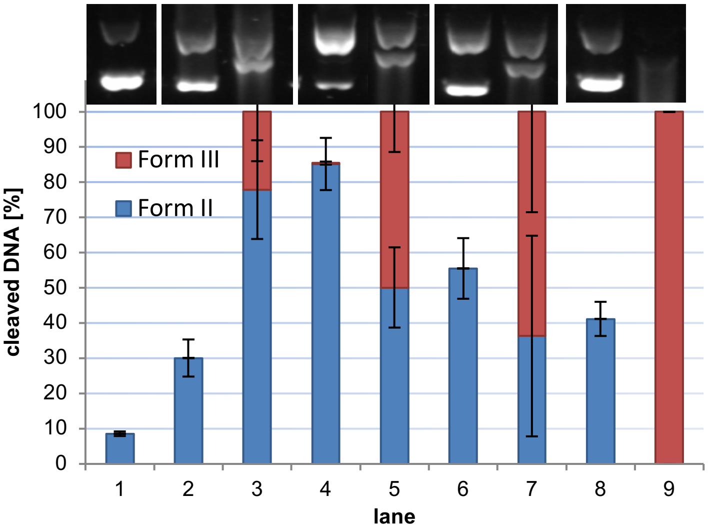

Figure S-8.5. Agarose gel and visualization of the cleavage of plasmid DNA pBR322 (0.2 µg) by the *in situ* formed complexes in presence and absence of ascorbate (250 µM) in MOPS buffer (10 mM, 100 mM NaCl, pH 7.4) after incubation for 1 h at 37 °C. Lane 1: DNA reference, lane 2: **CuC0** (60 µM), lane 3: **CuC0** (60 µM) + ascorbate, lane 4: **CuC6** (60 µM), lane 5: **CuC6** (60 µM) + ascorbate, lane 6: **CuC11** (60 µM), lane 7: **CuC11** (60 µM) + ascorbate, lane 8: **CuC16** (60 µM), lane 9: **CuC16** (60 µM) + ascorbate.

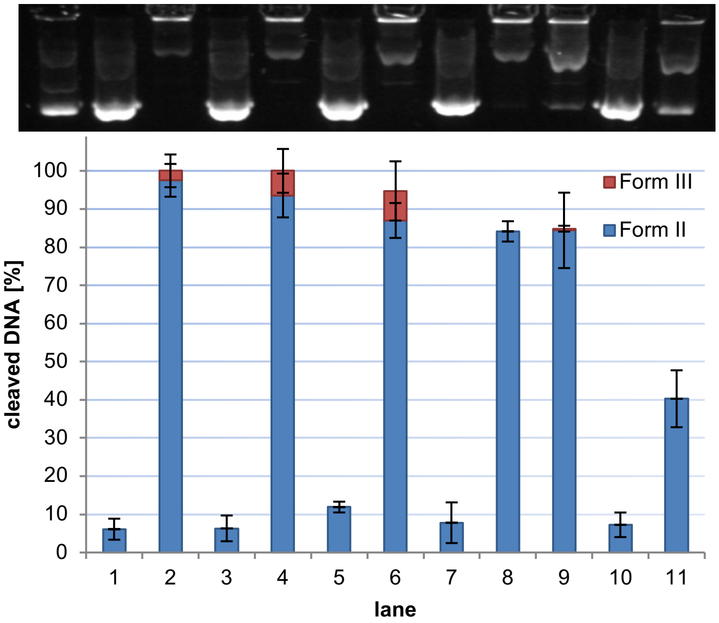

Figure S-8.6. Agarose gel and visualization of the cleavage of plasmid DNApBR322 (0.2 µg) by **CuC0** (70 µM) in MOPS buffer (10 mM, pH 7.4) after incubation for 1 h at 37 °C in the absence and presence of corresponding ROS scavengers. Lane 0: DNA ladder, lane 1: DNA reference, lane 2: **CuC0**, lane 3: DMSO (400 mM), lane 4: **CuC0** + DMSO (400 mM), lane 5: SOD (625 U/mL), lane 6: **CuC0** + SOD (625 U/mL), lane 7: pyruvate (2.5 mM), lane 8: **CuC0** + pyruvate (2.5 mM), lane 9: **CuC0** + pyruvate (2.5 mM) + SOD (625 U/mL), lane 10: NaN_3 (10 mM), lane 11: **CuC0** + NaN_3 (10 mM).

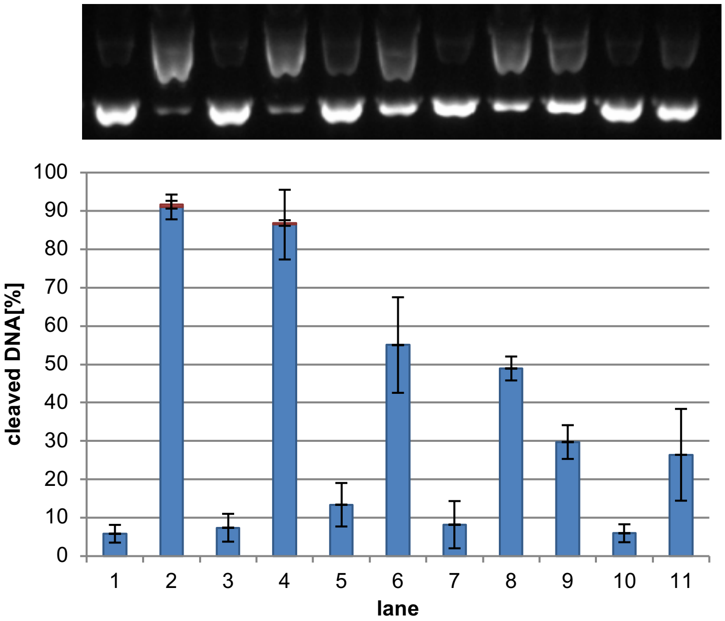

Figure S-8.7. Agarose gel and visualization of the cleavage of plasmid DNA pBR322 (0.2 µg) by **CuC11** (70 µM) in MOPS buffer (10 mM, pH 7.4, 100 mM NaCl) after incubation for 1 h at 37 °C in the absence and presence of corresponding ROS scavengers. Lane 1: DNA reference, lane 2: **CuC11**, lane 3: DMSO (400 mM), lane 4: **CuC11** + DMSO (400 mM), lane 5: SOD (625 u/mL), lane 6: **CuC11** + SOD (625 u/mL), lane 7: pyruvate (2.5 mM), lane 8: **CuC11** + pyruvate (2.5 mM), lane 9: **CuC11** + pyruvate (2.5 mM) + SOD (625 U/mL), lane 10: NaN_3 (10 mM), lane 11: **CuC11** + NaN_3 (10 mM).

S-9 Cytotoxicity

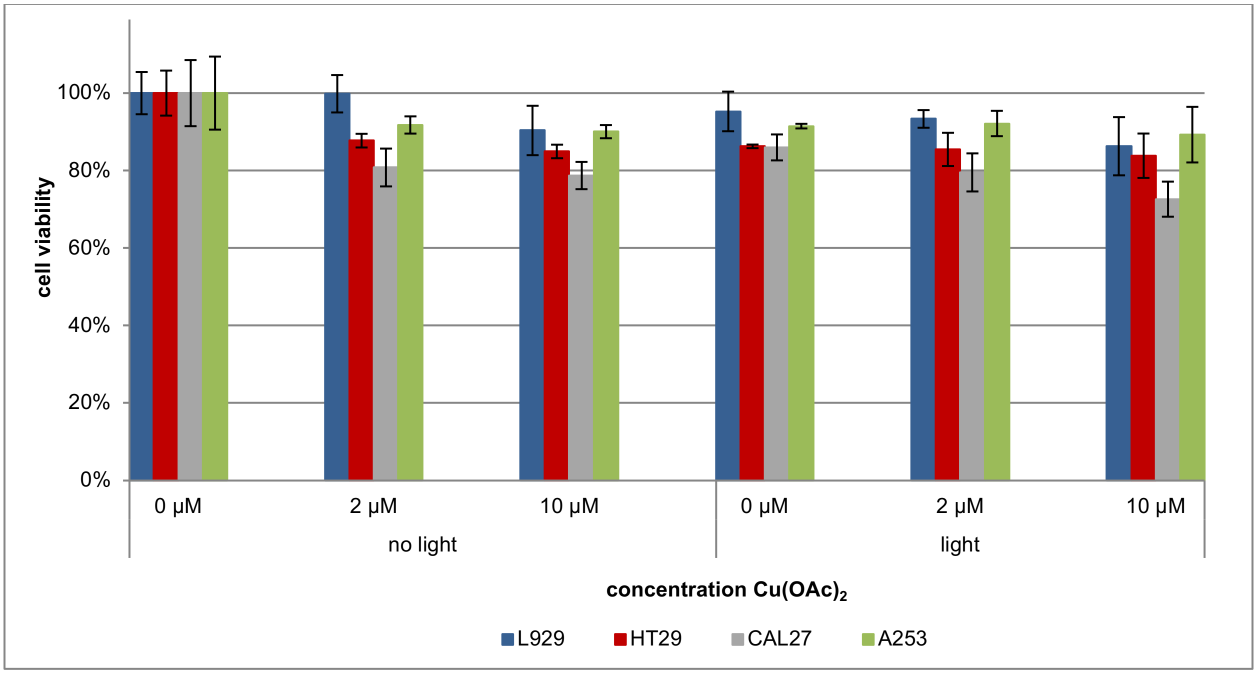

Figure S-9.1. Testing of cell viability of L929, HT29, CAL-27, and A253 cells in the presence of $Cu(OAc)_2$ (24 h incubation, white light source).

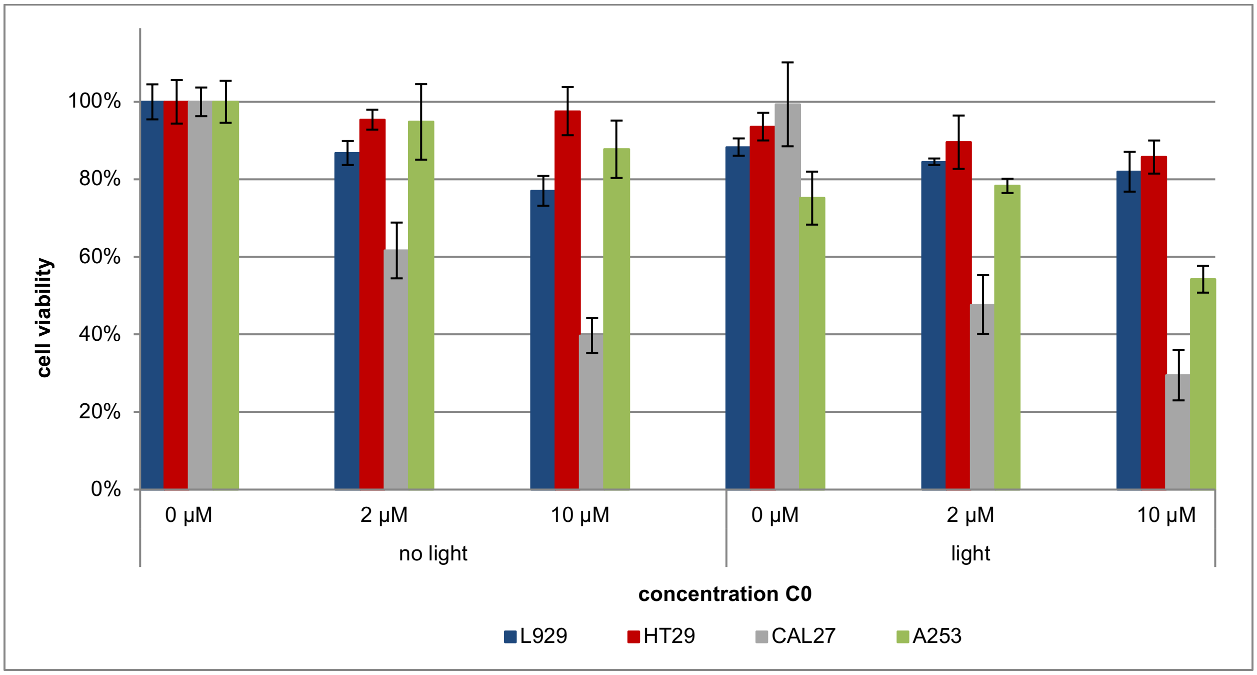

Figure S-9.2. Testing of cell viability of L929, HT29, CAL-27, and A253 cells in the presence of **C0** (24 h incubation, white light source).

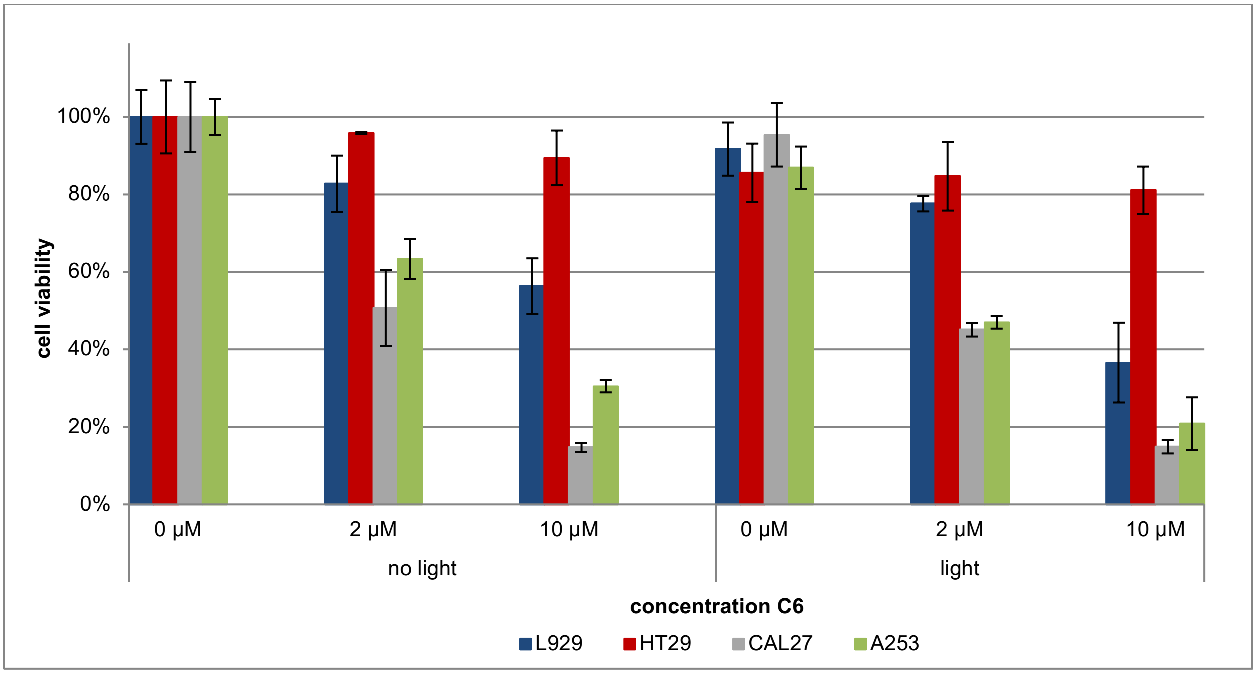

Figure S-9.3. Testing of cell viability of L929, HT29, CAL-27, and A253 cells in the presence of **C6** (24 h incubation, white light source).

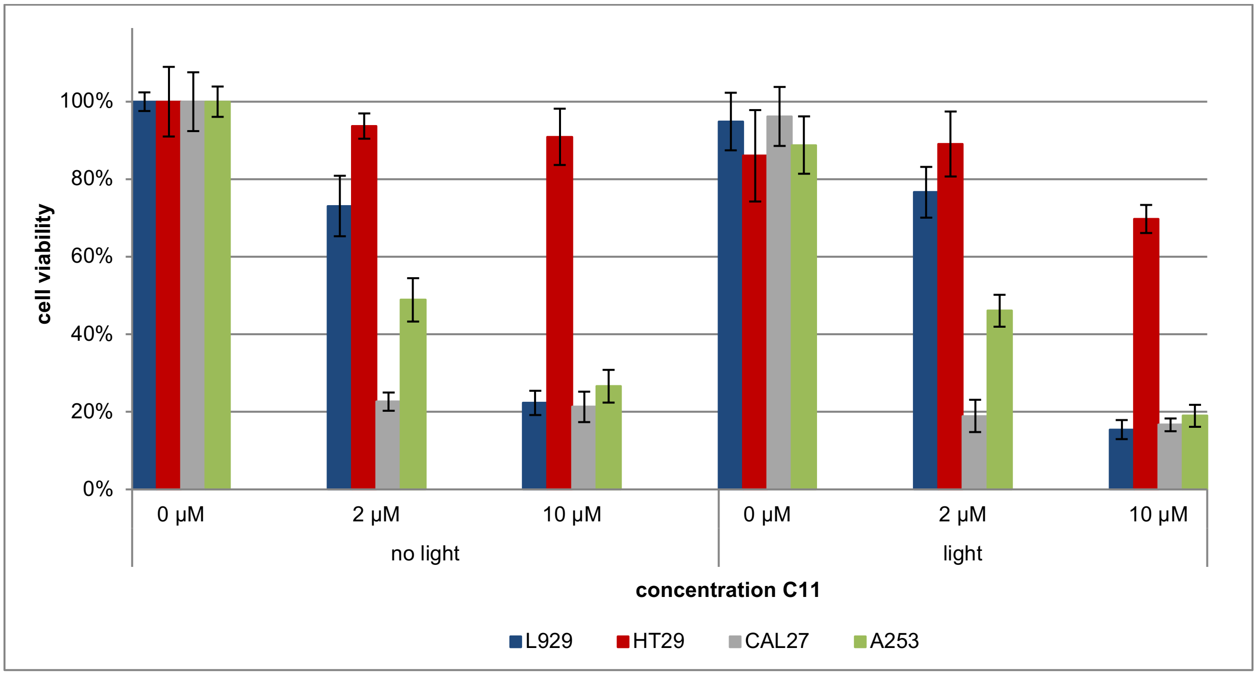

Figure S-9.4. Testing of cell viability of L929, HT29, CAL-27, and A253 cells in the presence of **C11** (24 h incubation, white light source).

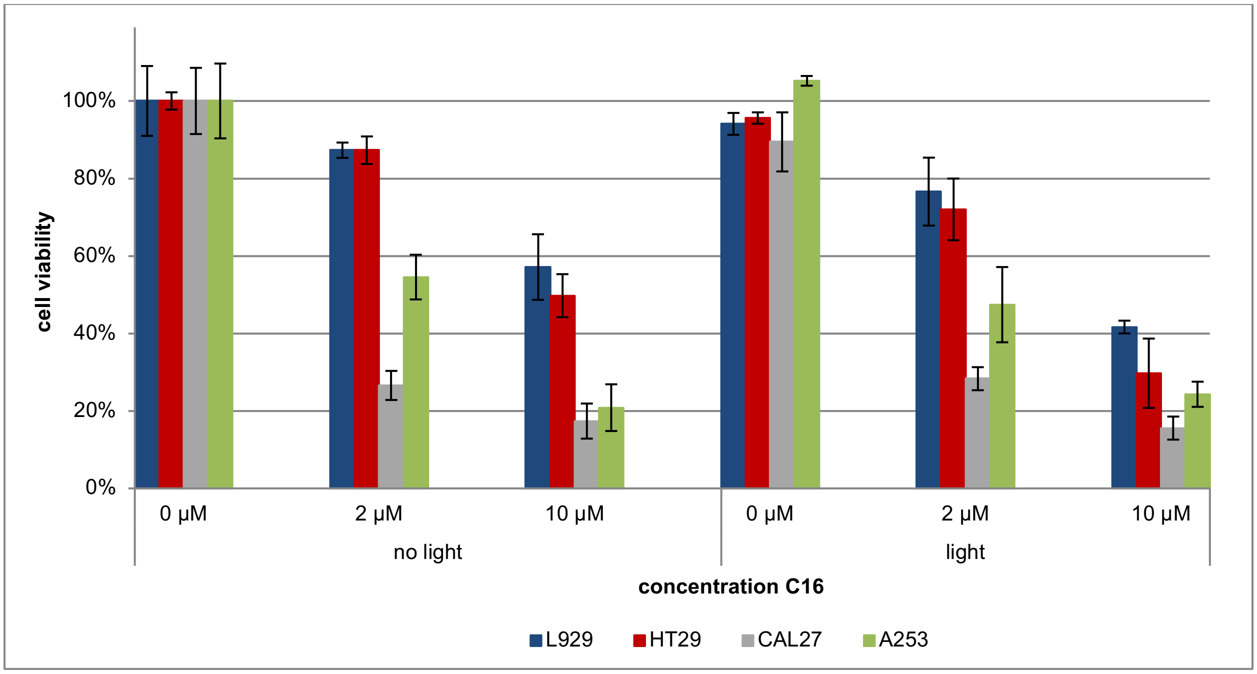

Figure S-9.5. Testing of cell viability of L929, HT29, CAL-27, and A253 cells in the presence of **C16** (24 h incubation, white light source).

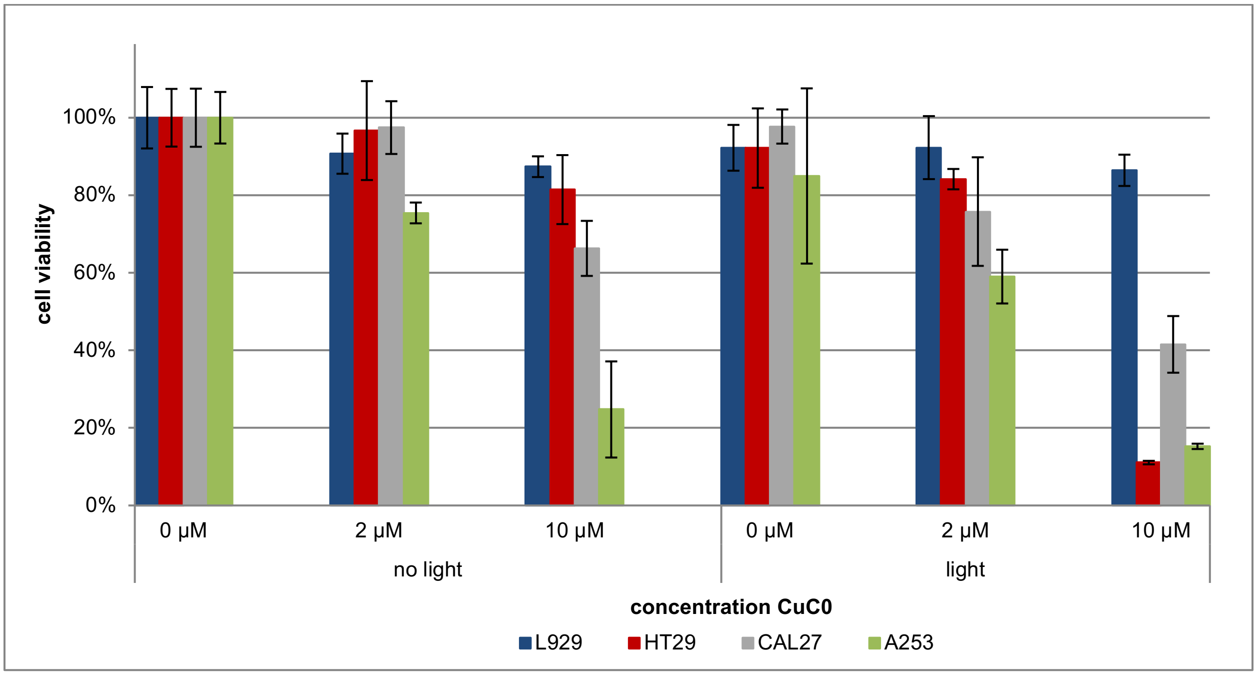

Figure S-9.6. Testing of cell viability of L929, HT29, CAL-27, and A253 cells in the presence of *in situ* formed **CuC0** (24 h incubation, white light source).

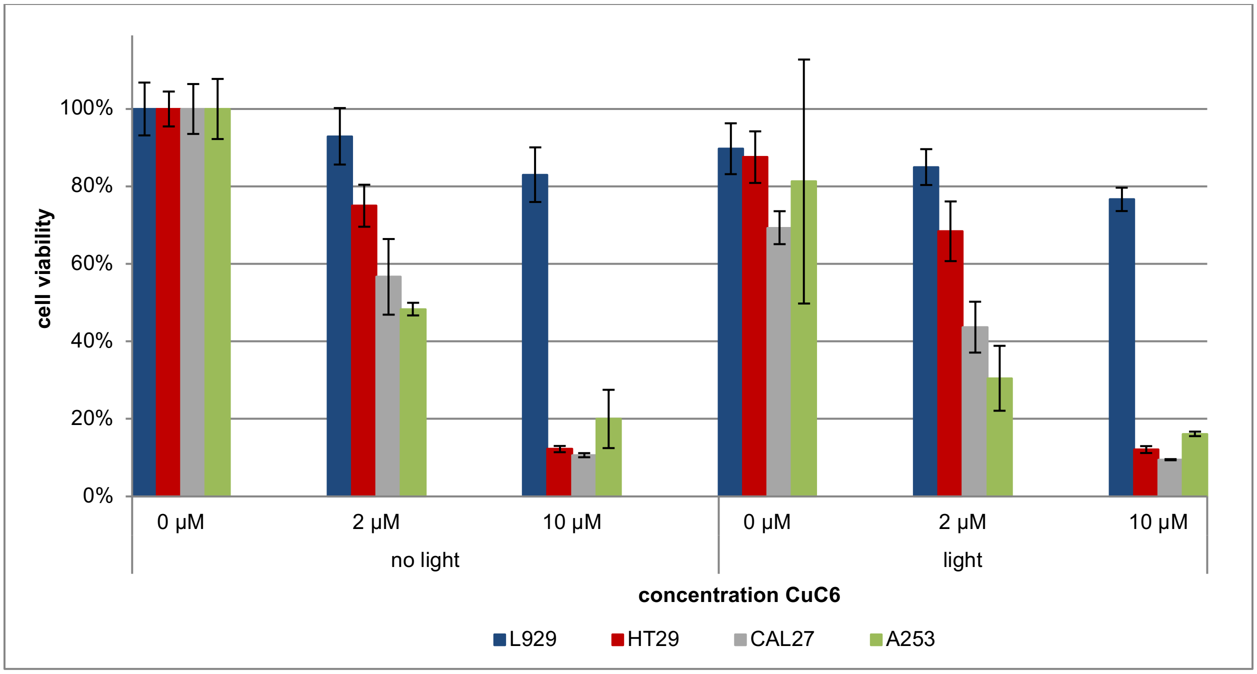

Figure S-9.7. Testing of cell viability of L929, HT29, CAL-27, and A253 cells in the presence of *in situ* formed **CuC6** (24 h incubation, white light source).

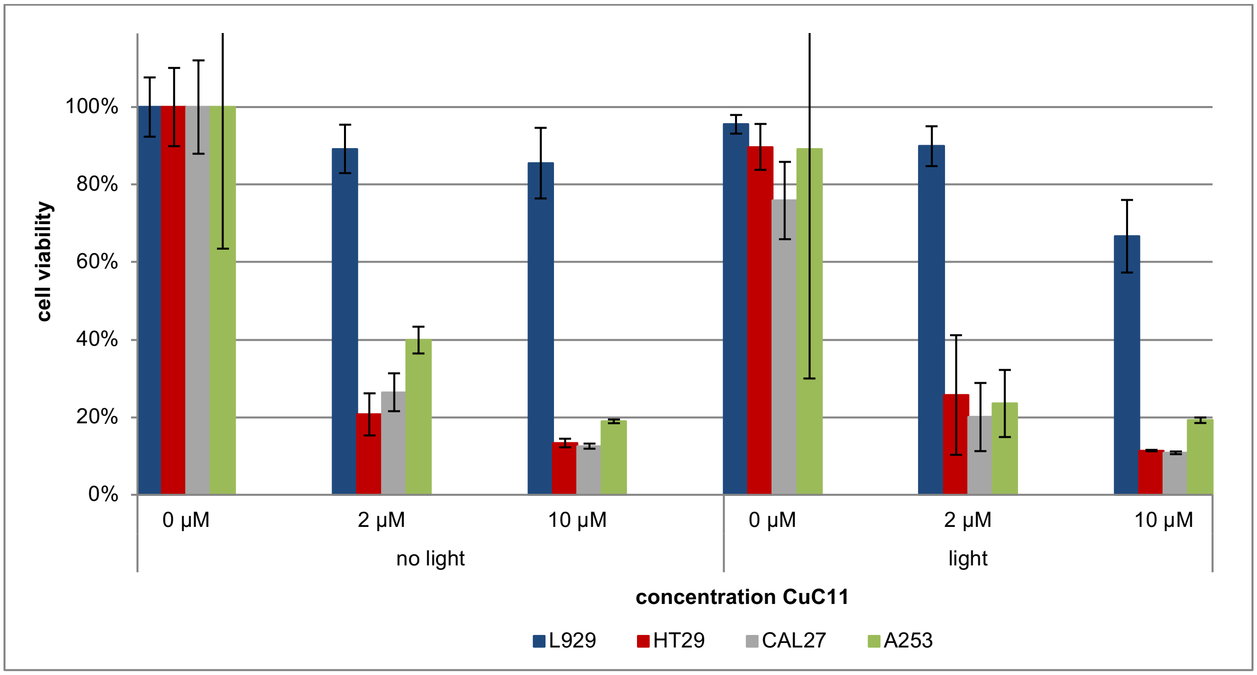

Figure S-9.8. Testing of cell viability of L929, HT29, CAL-27, and A253 cells in the presence of *in situ* formed **CuC11** (24 h incubation, white light source).

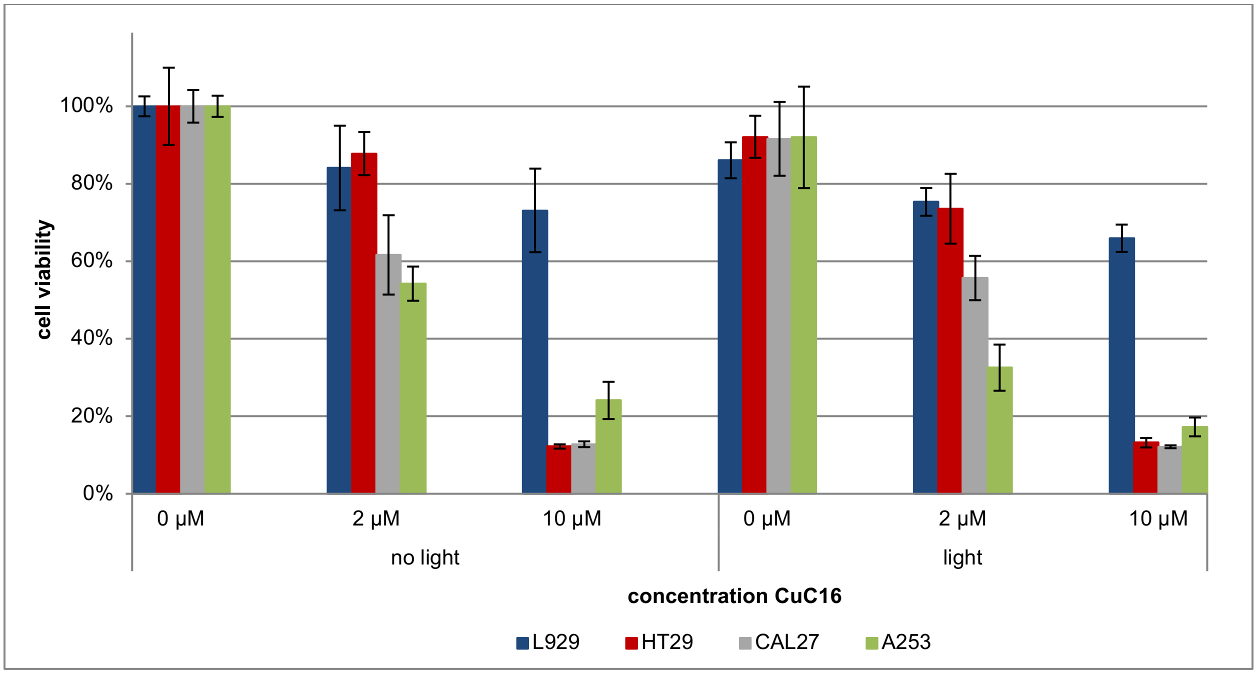

Figure S-9.9. Testing of cell viability of L929, HT29, CAL-27, and A253 cells in the presence of *in situ* formed **CuC16** (24 h incubation, white light source).

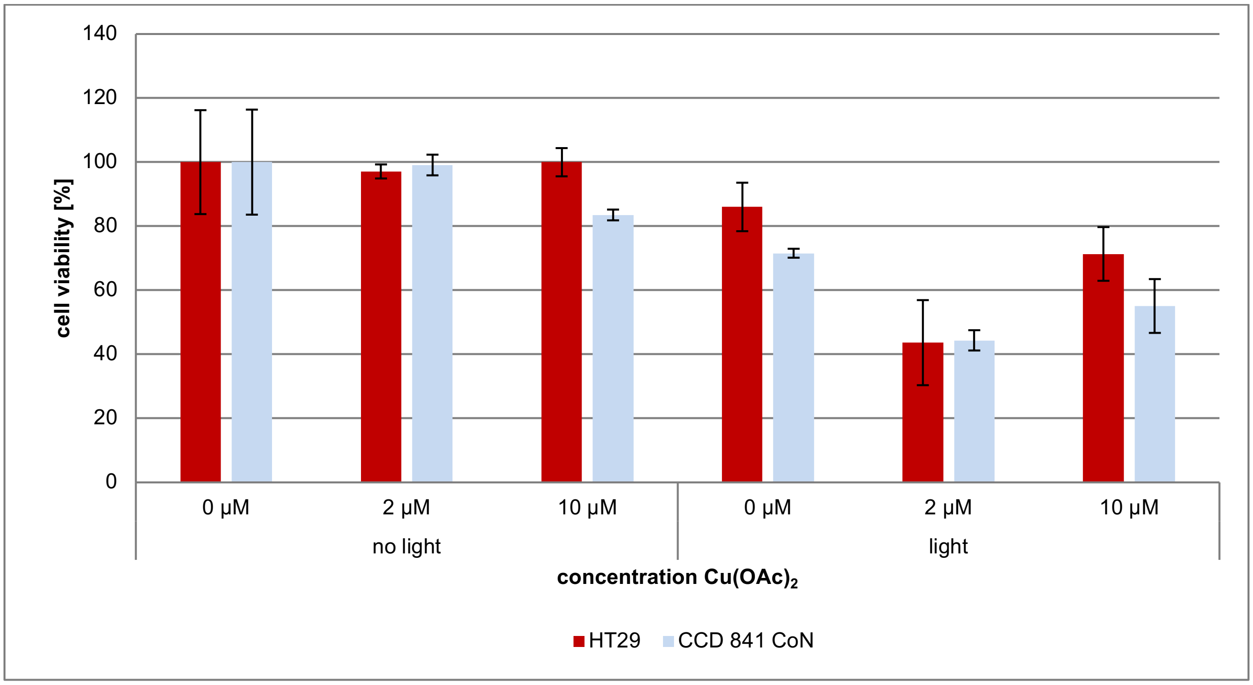

Figure S-9.10. Testing of cell viability of HT29 and CCD 841 CoN cells in the presence of $Cu(OAc)_2$ (24 h incubation, LED white light source).

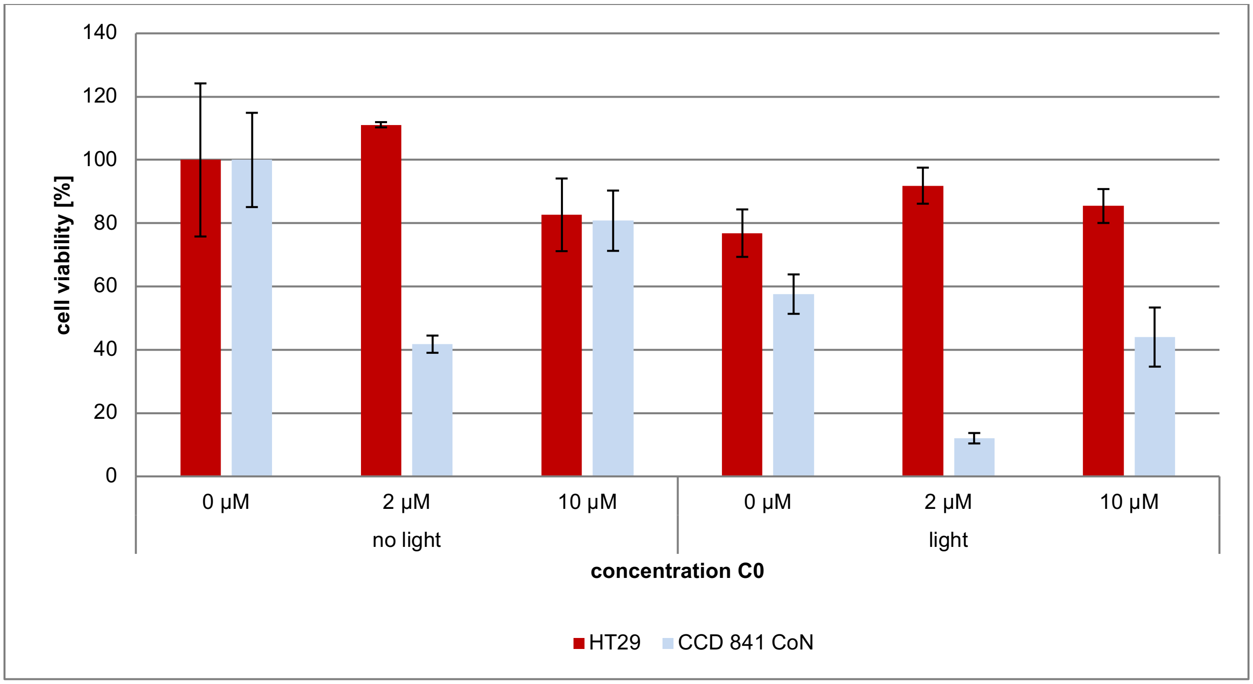

Figure S-9.11. Testing of cell viability of HT29 and CCD 841 CoN cells in the presence of **C0** (24 h incubation, LED white light source).

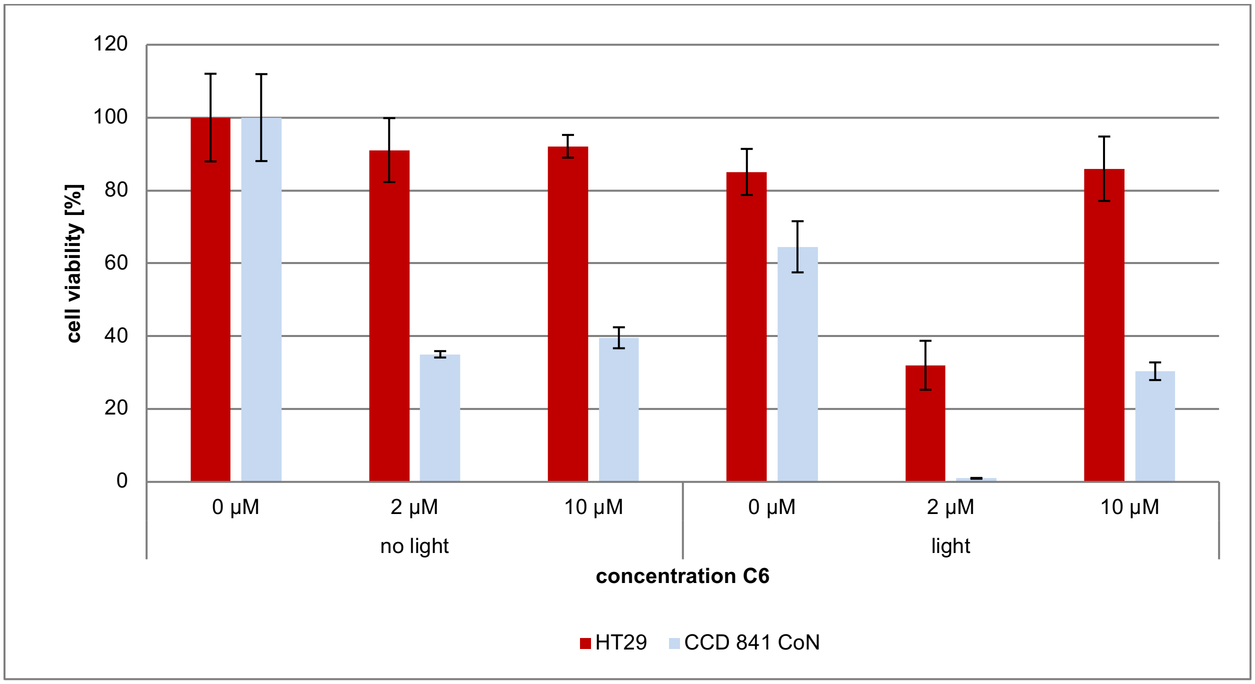

Figure S-9.12. Testing of cell viability of HT29 and CCD 841 CoN cells in the presence of **C6** (24 h incubation, LED white light source).

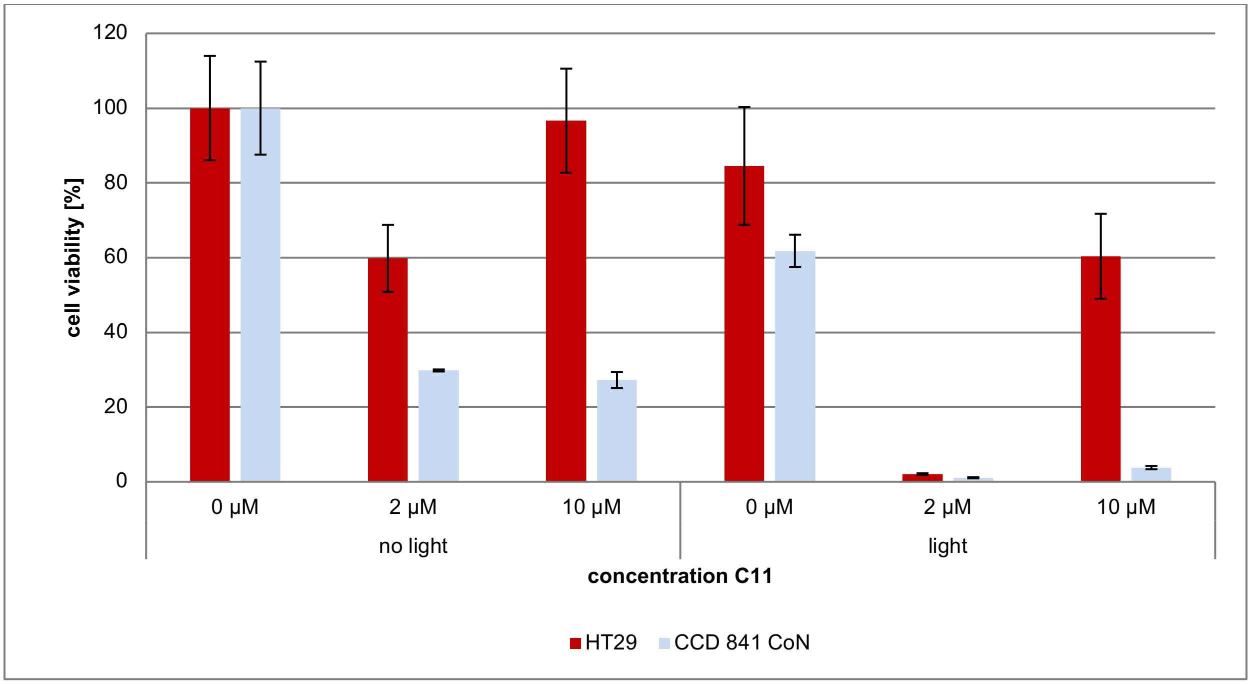

Figure S-9.13. Testing of cell viability of HT29 and CCD 841 CoN cells in the presence of **C11** (24 h incubation, LED white light source).

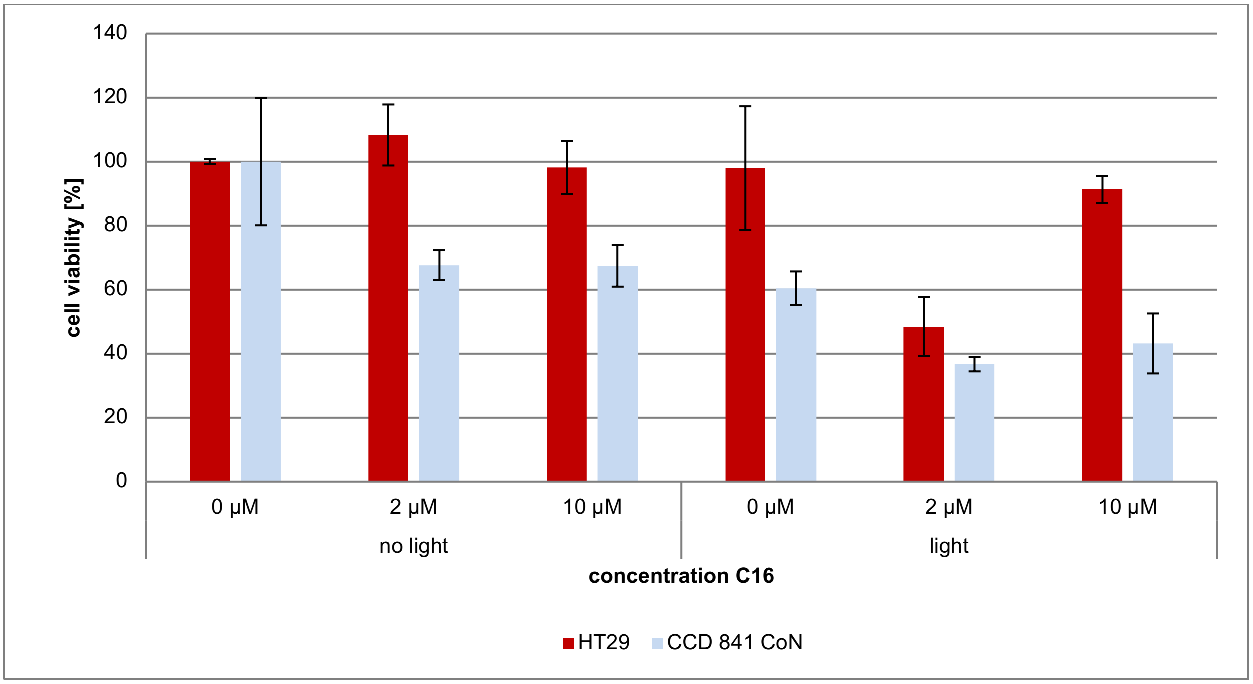

Figure S-9.14. Testing of cell viability of HT29 and CCD 841 CoN cells in the presence of **C16** (24 h incubation, LED white light source).

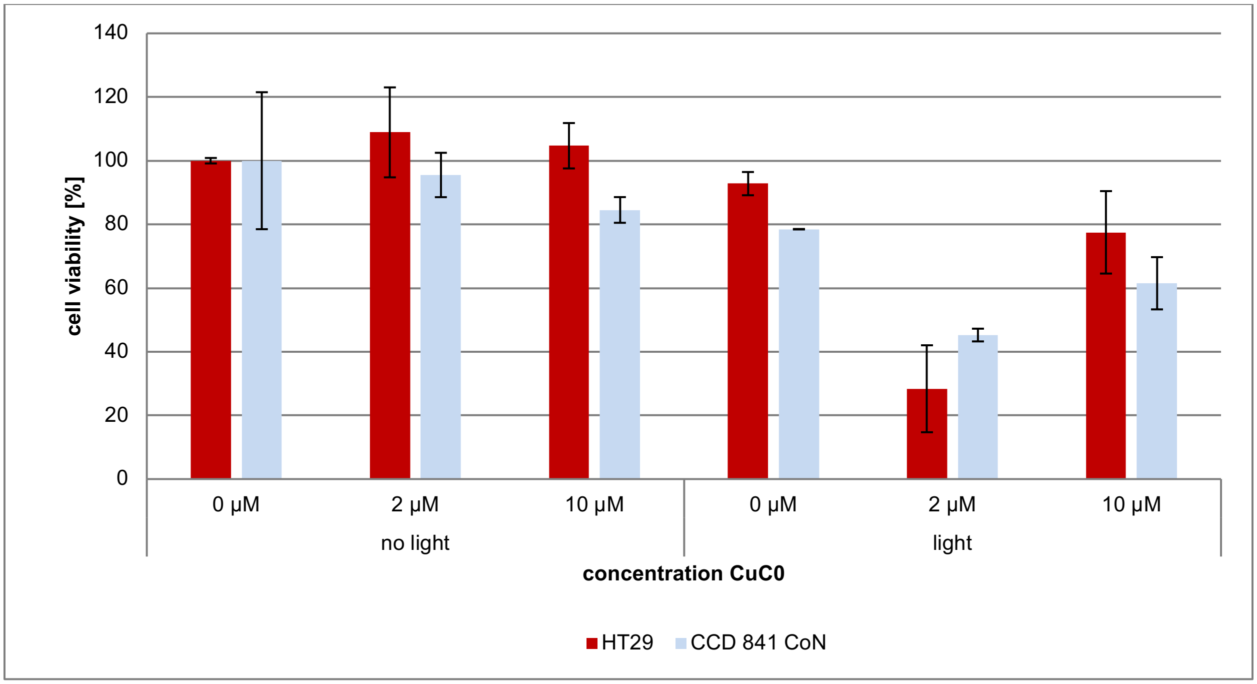

Figure S-9.15. Testing of cell viability of HT29 and CCD 841 CoN cells in the presence of *in situ* formed **CuC0** (24 h incubation, LED white light source).

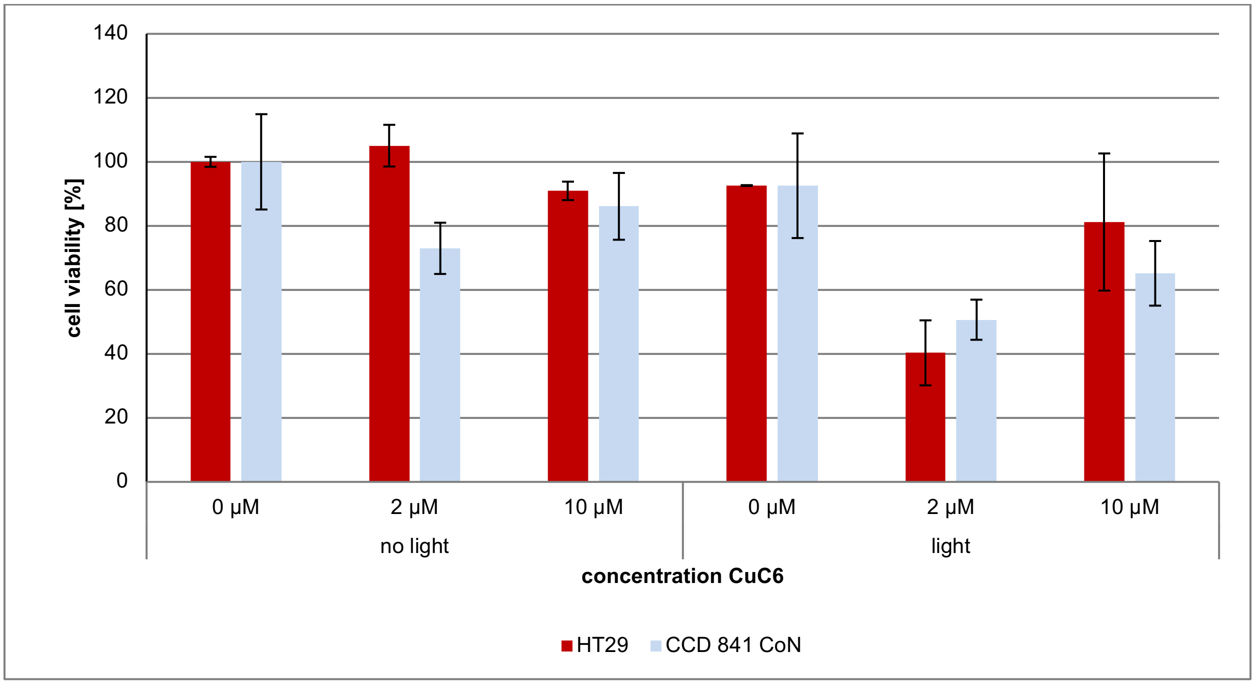

Figure S-9.16. Testing of cell viability of HT29 and CCD 841 CoN cells in the presence of *in situ* formed **CuC6** (24 h incubation, LED white light source).

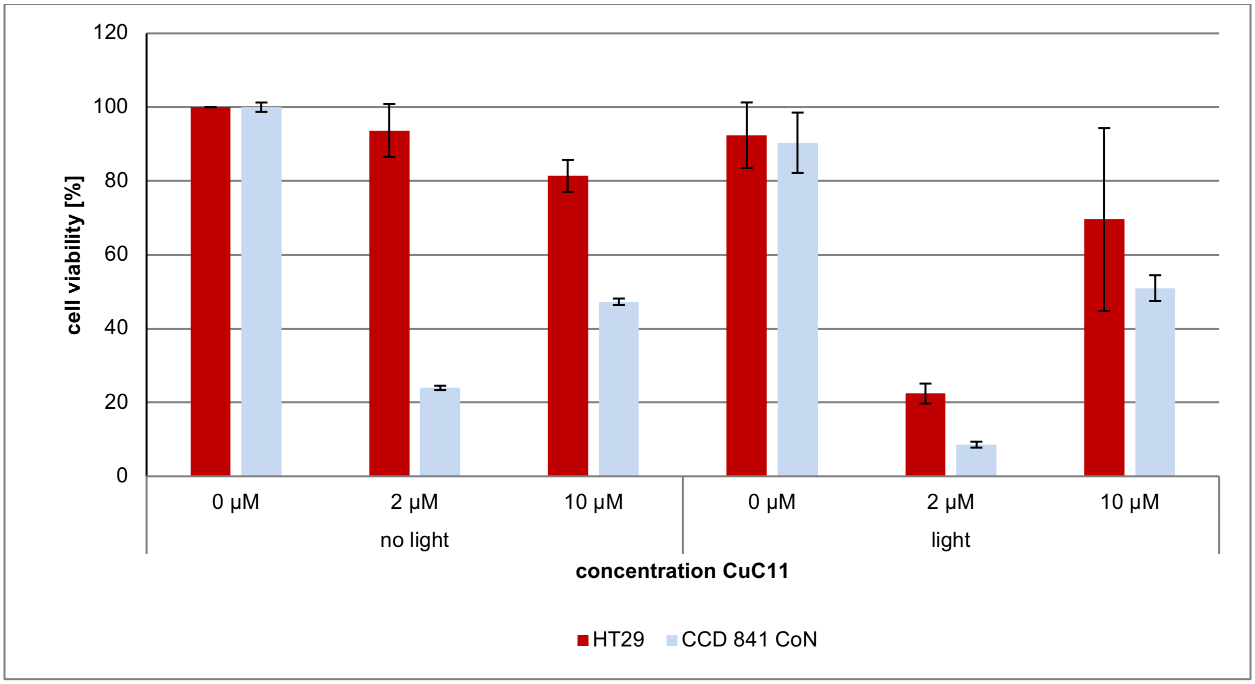

Figure S-9.17. Testing of cell viability of HT29 and CCD 841 CoN cells in the presence of *in situ* formed **CuC11** (24 h incubation, LED white light source).

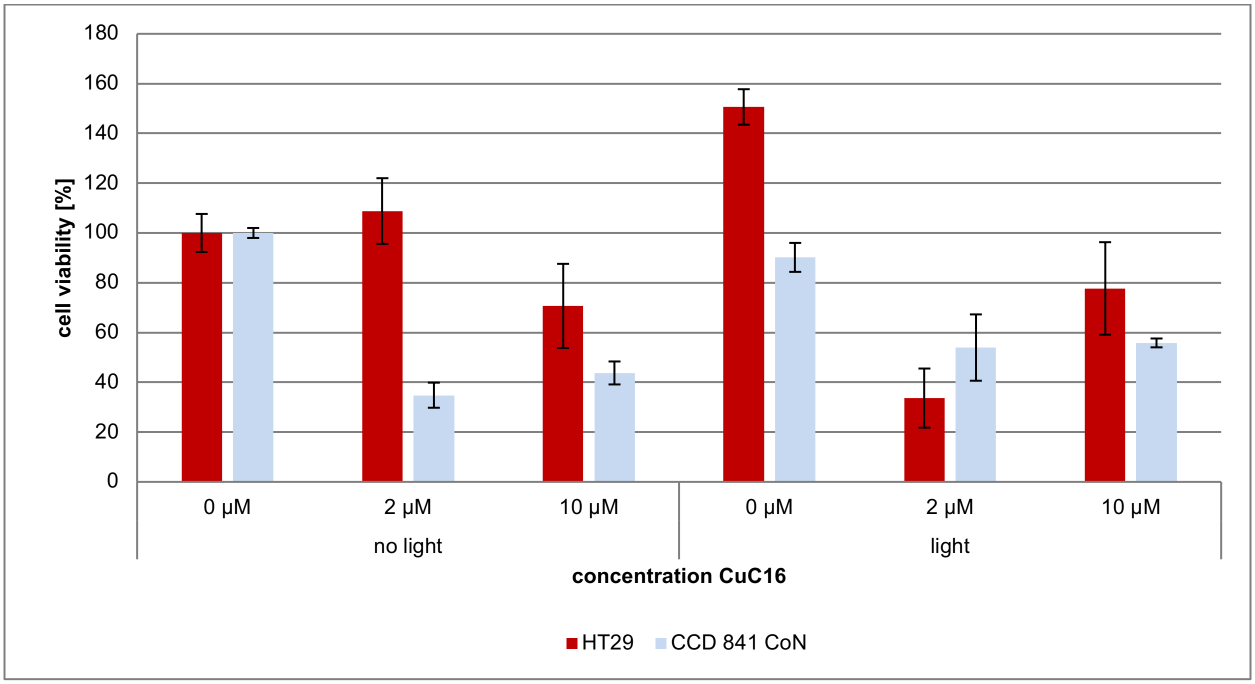

Figure S-9.18. Testing of cell viability of HT29 and CCD 841 CoN cells in the presence of *in situ* formed **CuC16** (24 h incubation, LED white light source).

S-10 ROS assay in cells

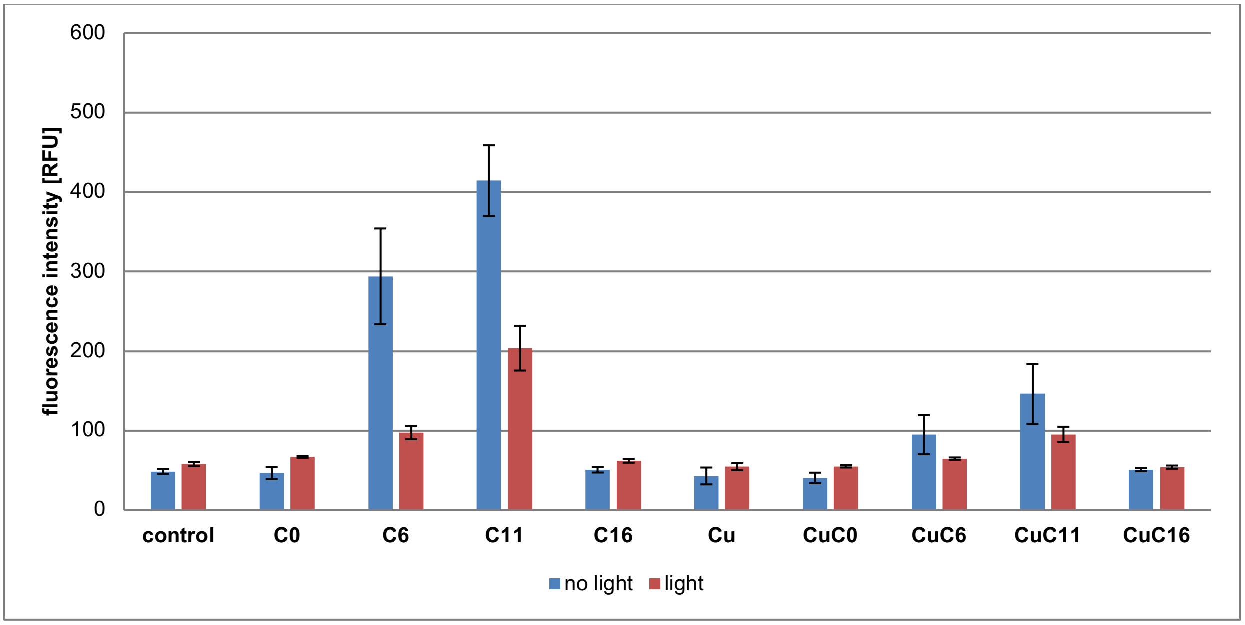

Figure S-9.19. Fluorescence intensity of CellROX® Orange measured by flow cytometry after 30 min incubation with HT29 cells having been treated with indicated prodigiosenes and *in situ* formed Cu(II) complexes as well as $Cu(OAc)_2$ (10μM, 1 h incubation, LED white light source).

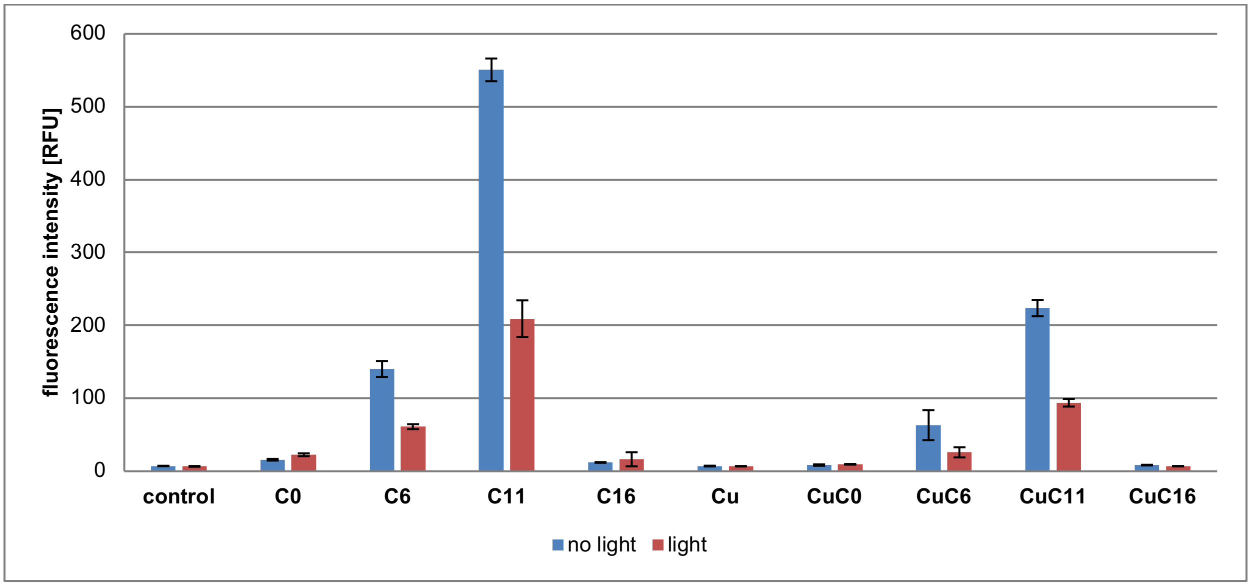

Figure S-9.20. Fluorescence intensity of CellROX® Orange measured by flow cytometry after 30 min incubation with CCD 841 CoN cells having been treated with indicated prodigiosenes and *in situ* formed Cu(II) complexes as well as $Cu(OAc)_2$ (10 μM, 1 h incubation, LED white light source).

S-11 Antimicrobial susceptibility testing

Table S-11. Overview of the field isolates tested

Isolate ID	Host animal species	Bacterial species	Methicillin resistance
IMT 40938	cat	*Staphylococcus aureus*	resistant
IMT 41387	cat	*Staphylococcus pseudintermedius*	resistant
IMT 41475	dog	*Staphylococcus pseudintermedius*	susceptible
IMT 43229	cat	*Staphylococcus aureus*	susceptible
IMT 43255	dog	*Staphylococcus pseudintermedius*	susceptible
Rd 6	cattle	*Staphylococcus aureus*	resistant
Rd 10R	cattle	*Staphylococcus aureus*	resistant
Rd 10S	cattle	*Staphylococcus aureus*	resistant
Rd 11	cattle	*Staphylococcus aureus*	resistant
Rd 42	cattle	*Staphylococcus aureus*	susceptible
Rd 58	cattle	*Staphylococcus aureus*	resistant
49496	horse	*Staphylococcus aureus*	borderline resistant
IMT 39173	horse	*Staphylococcus aureus*	borderline resistant
IMT 39637	horse	*Staphylococcus aureus*	borderline resistant
IMT 37083	horse	*Staphylococcus aureus*	borderline resistant

References

[1] G. Park, J. T. Tomlinson, M. S. Melvin, M. W. Wright, C. S. Day, R. A. Manderville, *Org. Lett.* **2003**, *5*, 113–116.

[2] R. P. Hertzberg, P. B. Dervan, *Biochemistry* **1984**, *23*, 3934–3945.

[3] M. V. Berridge, P. M. Herst, A. S. Tan, *Biotechnol. Annu. Rev.* **2005**, *11*, 127–152.

[4] Performance Standards for Antimicrobial Disk and Dilution Susceptibility Tests for Bacteria Isolated From Animals, 5th ed. CLSI standard VET01. Wayne, Clinical and Laboratory Standards Institute, Wayne, PA, USA, **2018**.

[5] Performance Standards for Antimicrobial Disk and Dilution Susceptibility Tests for Bacteria Isolated From Animals, 5th ed. CLSI supplement VET01S. Clinical and Laboratory Standards Institute, Wayne, PA, USA, **2020**.

[6] R. D'Alessio, A. Rossi, *Synlett* **1996**, *1996*, 513–514.

[7] H. H. Wasserman, G. C. Rodgers, D. D. Keith, *Tetrahedron* **1976**, *32*, 1851–1854.

[8] S. Martina, V. Enkelmann, G. Wegner, A.-D. Schlüter, *Synthesis* **1991**, *1991*, 613–615.

[9] E. Tyrrell, P. Brookes, *Synthesis* **2003**, *2003*, 469–483.

[10] D. M. Knapp, E. P. Gillis, M. D. Burke, *J. Am. Chem. Soc.* **2009**, *131*, 6961–6963.

[11] P. A. Cox, A. G. Leach, A. D. Campbell, G. C. Lloyd-Jones, *J. Am. Chem. Soc.* **2016**, *138*, 9145–9157.

[12] P. A. Cox, M. Reid, A. G. Leach, A. D. Campbell, E. J. King, G. C. Lloyd-Jones, *J. Am. Chem. Soc.* **2017**, *139*, 13156–13165.

[13] R. Greenhouse, C. Ramirez, J. M. Muchowski, *J. Org. Chem.* **1985**, *50*, 2961–2965.

[14] K. Papireddy, M. Smilkstein, J. X. Kelly, Shweta, S. M. Salem, M. Alhamadsheh, S. W. Haynes, G. L. Challis, K. A. Reynolds, *J. Med. Chem.* **2011**, *54*, 5296–5306.

[15] B. Jolicoeur, W. D. Lubell, *Can. J. Chem.* **2008**, *86*, 213–218.

[16] A. R. Morgan, J. S. Lee, D. E. Pulleyblank, N. L. Murray, D. H. Evans, *Nucleic Acids Res.* **1979**, *7*, 547–569.
